# Chemoenzymatic Synthesis of the Most Pleasant Stereoisomer of Jessemal

**DOI:** 10.1021/acs.joc.2c00427

**Published:** 2022-04-20

**Authors:** Silvia Venturi, Milos Trajkovic, Danilo Colombo, Elisabetta Brenna, Marco W. Fraaije, Francesco G. Gatti, Piero Macchi, Emilio Zamboni

**Affiliations:** † Dipartimento di Chimica, Materiali ed Ingegneria Chimica “G. Natta”, Politecnico di Milano, P.zza Leonardo da Vinci 32, 20133 Milano, Italy; ‡ Molecular Enzymology Group, University of Groningen, Nijenborgh 4, 9747 AG Groningen, The Netherlands

## Abstract

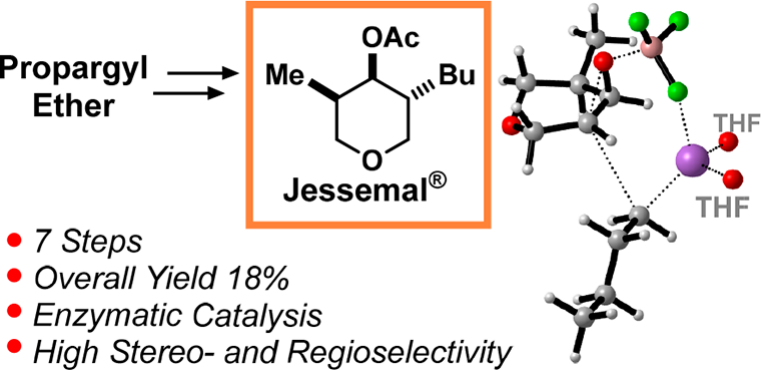

We describe the asymmetric synthesis of the most pleasant enantiomer of Jessemal fragrance. The key steps are (i) the one-pot reduction of an α-chloro-tetrasubstituted cyclohexenone to give the chlorohydrin, catalyzed by two stereoselective redox enzymes (an ene-reductase and an alcohol dehydrogenase); (ii) the regioselective epoxide ring-opening with organocuprate or organolithium nucleophiles. Density functional theory calculations together with the Curtin–Hammett principle allowed the rationalization of the regioselectivity.

The odor perception of chiral molecules depends on their stereochemical configuration.^1^ In the past two decades, the olfactory properties (odor threshold and odor profile) of many commercial fragrances and flavors have been thoroughly investigated.^1b^ Often, significant differences between the enantiomers were observed.^2^

However, in addition to the increasing interest of industry in the formulations of new perfumes selecting only the most pleasant stereoisomers of fragrances, the use of enantiomerically enriched odorants may soon become mandatory for health and environmental protection reasons. Indeed, there are many concerns about the toxicity of fragrances, especially when they are used for a prolonged time. Recent studies have shown that some musk odorants are not metabolized by our organism, and accumulate into the human tissues and organs. In this regard, the case of the Galaxolide fragrance detected in breast milk is quite alarming.^3^ Hence, only by using the most odor active stereoisomers will it be possible to decrease the amount of ingredients actually used in all formulations, and therefore limit the related risks.

This issue becomes more relevant for all synthetic fragrances with more than one stereogenic center, such as the Jessemal, i.e., **1** (Figure 1A).^4^ Nevertheless, to our knowledge, very a few chiral fragrances are commercialized as single enantiomer.

**Figure 1:**
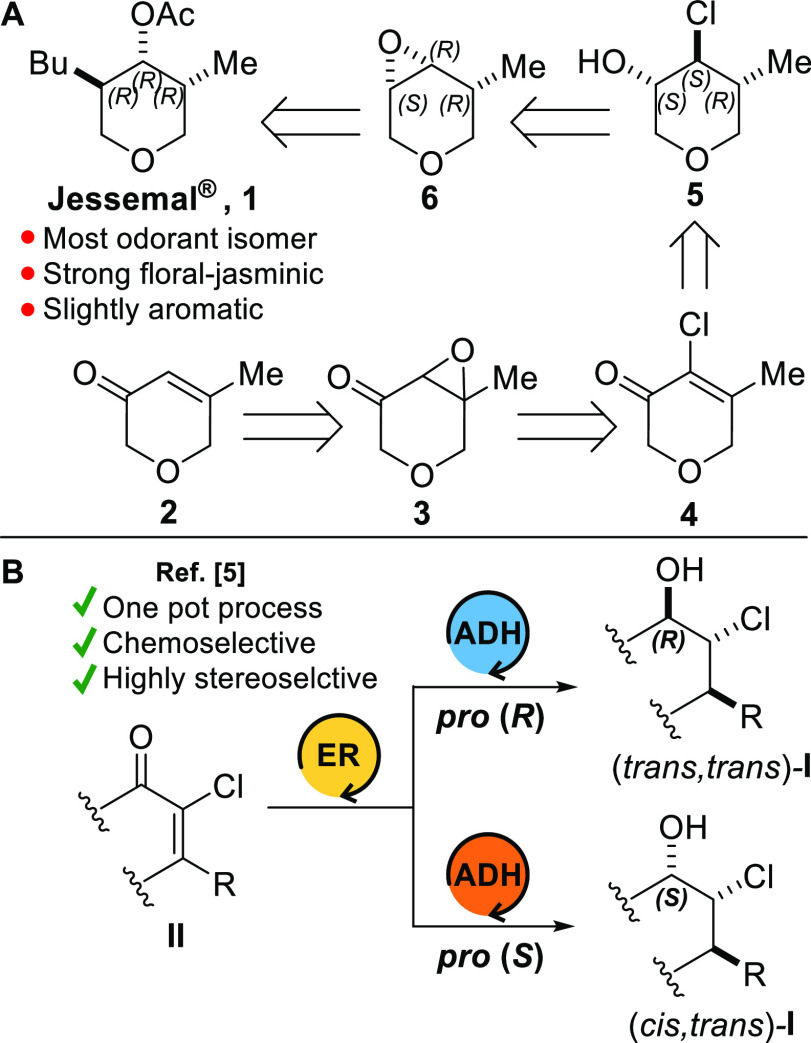
(A) Retrosynthesis of Jessemal. (B) One-pot multienzymatic stereoselective reduction of α-chloro cycloenones.

In the case of Jessemal, an organoleptic study has identified the enantiomer (3*R*,4*R*,5*R*)-**1** as the most pleasant and that with the most characterizing floral scent;^4^ thus, its stereoselective synthesis is highly desirable.

Recently, we disclosed a new route to the β-alkyl chlorohydrins **I** by reducing the cycloenones **II** (Figure 1B).^5^ The one-pot two-steps sequence was carried out combining two redox enzymes. An ene-reductase (ER) catalyzed the stereospecific reduction of the C=C double bond affording the intermediate ketone, which in turn was reduced by an alcohol dehydrogenase (ADH). The choice of a *pro* (*R*) or a *pro* (*S*) ADH allowed to control the relative stereochemistry of the chlorohydrins ((*trans*,*trans*)-**I** or (*cis*,*trans*)-**I**); both yield and optical purity were very high in most cases. Accordingly, we designed the retrosynthesis of **1** shown in Figure 1A. Regarding the reduction of prochiral C=C double bonds conjugated with EWGs, the ERs are becoming quite popular in organic synthesis,^6,7^ since they are a valuable alternative to all methodologies based on asymmetric hydrogenations catalyzed by organo–transition metal complexes. Lastly, this enzymatic activity has been proven to be compatible with that of ADHs, allowing the setup of efficient multienzymatic cascade processes.^8^

Thus, we designed a retrosynthesis of **1** relying on the new synthetic route to the *trans*,*trans* chlorohydrins **I** (Figure 1A). Indeed, *trans*,*trans*-**5** can be transformed into the epoxide **6**, and its C(3) regioselective ring-opening leads, after acetylation, to **1**. Accordingly, we prepared the substrate **4** needed for the enzymatic reduction in four steps starting from the commercially available dipropargyl ether in an overall yield of 53%, modifying a known procedure^9^ (Scheme S1 in the Supporting Information). Remarkably, the preparation of **4** is a column chromatography free procedure.

The ER reaction mechanism consists of a stepwise addition of two hydrogen atoms, which come from opposite faces of the C=C double bond affording the product with *anti* stereochemistry.^10^ Recently, deazaflavin cofactor (F420) dependent ene-reductases (FDRs) were shown to exhibit opposite stereospecificity to that of most common flavin mononucleotide (FMN) cofactor dependent ERs,^11^ such as the reductases belonging to the old yellow enzyme (OYE) family (Figure S1). The opposite stereochemical course was explained in terms of different binding of the substrate into the protein catalytic site. Historically, the binding mode of the 3-methylcyclohex-2-en-1-one affording the (*S*) enantiomer was arbitrary named “flipped” (Figure S1B, typical of OYEs), vice versa that giving the opposite enantiomer was named “normal” (Figure S1A, typical for FDRs).

Thus, with the chloro cyclohexenone **4** in our hands, we tested the enzymatic reduction [(1) ER + (2) ADH] on a screening scale. According to our previous works,^5,11^ the combination of an ER belonging to the FDR family with the commercially available ADH named EVO270 should yield the (*trans*,*trans*) chlorohydrin, having absolute stereochemical configuration suited to synthesize (3*R*,4*R*,5*R*)-**1**. Since EVO270 catalyzes the reduction of prochiral ketones with *pro* (*S*) enantioselectivity, substrates very similar to **4** are placed into the catalytic site of FDRs through a “normal” binding mode.

The reduced forms of cofactors needed for the reduction of **4**, i.e., NADPH and F_420_H_2_, were efficiently regenerated by a glucose dehydrogenase (GDH, from *Bacillus megaterium*)^12^ and a F420-dependent glucose-6-phosphate dehydrogenase (FGD, from *Rhodococcus jostii*), using an excess of glucose and glucose-6-phosphate as sacrificial substrates,^13^ respectively (Figure S1C). Conversions and diastereomeric excesses (*de*) of the screening are available in the Supporting Information (Table S1 and Table S3). The data show clearly that **4** is a substrate well accepted by the FDRs (FDR-Rha1 and FDR-Rha2 isolated from *R. jostii* and FDR-Mha from *Mycobacterium hassiacum*), since all transformations were near to being quantitative. The diastereoselectivity of the multienzymatic process (FDR-Mha+EVO270) was good. However, only by scaling up the reaction it was possible to determinate the relative stereochemistry of the product by ^1^H NMR (Figure S2). Surprisingly, instead of the expected (*trans*,*trans*) diastereoisomer, we obtained the (*cis*,*trans*) chlorohydrin, i.e., (3*R*,4*S*,5*R*)-**5** (vide infra for the absolute stereochemical configuration), in 69% yield and with a *de* of 99%, after column chromatography (Table 1).

**Table 1:**
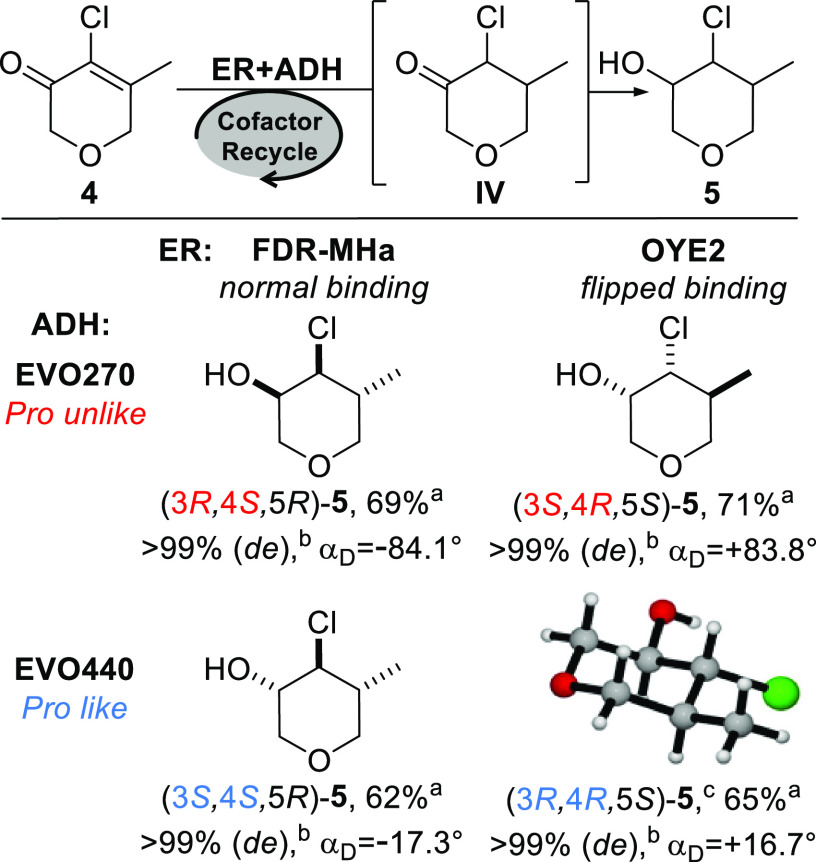
Multienzymatic Reduction of **4**^d^

a Yield after purification.

b By ^1^H NMR.

c Absolute stereochemical configuration determined from crystal X-ray diffraction.

d Reaction conditions: **4** (3.0 mmol) in buffer (50 mM), cosolvent (1% v/v) at 24–30 °C, 150 rpm. More details are given in the Supporting Information.

The *trans* stereochemical relationship between the methyl and chloride substituents confirms that the FDR catalyzed reductions proceed by formal addition of H_2_ with *anti* stereospecificity, whereas the *cis* relation between the chloride and the hydroxyl group could be explained either by a “flipped” binding mode of **4** into the catalytic site of FDR or by a reversed stereoselectivity of EVO270. For this, a commercially available *pro* (*R*) ADH (EVO440) and some NADPH-dependent ERs (OYE1–3) enantiodivergent (“flipped” binding mode) to the FDRs were tested. Analysis was first done on a screening scale (Table S2 and Table S4), and then on a preparative scale selecting the best combination of enzymes in terms of diastereoselectivity (Table 1). Even though the conversions of the biotransformations on the preparative scale were near quantitative, the isolation of products was rather difficult due to their high volatility. However, the final yields and *de*’s were satisfactory, and remarkably, each enantiomer of the (*trans*,*trans*) chlorohydrin was isolated by crystallization. In summary, the reductions carried out on a preparative scale allowed to improve the diastereomeric purity of chlorohydrines **5** by means of purification procedures.

The absolute stereochemical configuration (3*R*,4*R*,5*S*) was assigned to the chlorohydrin (*trans*,*trans*)-**5** obtained from the tandem reduction with OYE2 and EVO440, by estimation of the Flack parameter. The latter was calculated from the single crystal X-ray diffraction model (Table 1, for more details see the Supporting Information). Consequentially, it was found that the stereoisomer (3*S*,4*S*,5*R*)-**5**, needed for the synthesis of Jessemal, was formed using FDR-Mha and EVO440.

Table 1 shows that the stereoselectivity of both ADHs depends on the absolute stereochemical configuration of the intermediate **IV**. The stereochemical course of the 1,2-carbonyl reduction was better explained by adopting the Prelog-Seebach specifications^14^ (Figure S3). In summary, we have found that EVO270 exhibits a *pro* (*like*) stereospecificity, whereas EVO440 is *pro* (*unlike*). Unfortunately, further considerations on how the substrates are bound into the catalytic sites were not possible, since both identity and structure of these two commercial ADHs are not available.

Following our synthetic plan, we prepared the epoxide **6** by treatment of (3*S*,4*S*,5*R*)-**5** with *t*-BuOK (THF, 1.3 equiv, 0 °C) in a yield of 80% (Scheme 1A). Concerning the ring-opening of the tetrahydropyranyl 3,4-epoxide, Crotti et al. have shown that the addition of carbon nucleophiles such as the organocuprate or organoaluminum reagents occurs preferentially at C(4) position.^15^ The high regioselectivity was ascribed to the formation of a complex adduct between the oxygen atoms of epoxide and tetrahydropyranyl rings with the metal cation (aluminum or copper). It was proposed that the metal cation locked the tetrahydropyranyl unit into a specific conformation (chair-like), which in turn favored the nucleophilic attack on the less hindered carbon (Scheme 1B). Therefore, we attempted the ring-opening with a different carbon nucleophile. Treatment of **6** with *n*-butyl lithium (2.5 equiv) in the presence of BF_3_·Et_2_O (2.5 equiv) in THF at low temperature (−78 °C) gave mainly the C(3) type regioisomer alcohol,^16^ i.e., (3*R*,4*R*,5*R*)-**7**, (**7**/**7a**, 82:18, by GC-MS). The latter was isolated by column chromatography separation in a good yield of 72%, conserving the initial high diastereomeric excess (*de* > 99% by GC-MS) and with a good optical purity ([α]_D_ = −74.0° vs [α]_D_ = −78.7°,^4^ CHCl_3_). Then, alcohol **7** was acetylated with Ac_2_O affording the most pleasant stereoisomer of Jessemal, i.e., (3*R*,4*R*,5R)-**1**, in a yield of 95%, and with a high optical purity ([α]_D_ = −59.1° vs [α]_D_ = −62.9°,^4^ CHCl_3_).

**Scheme 1:**
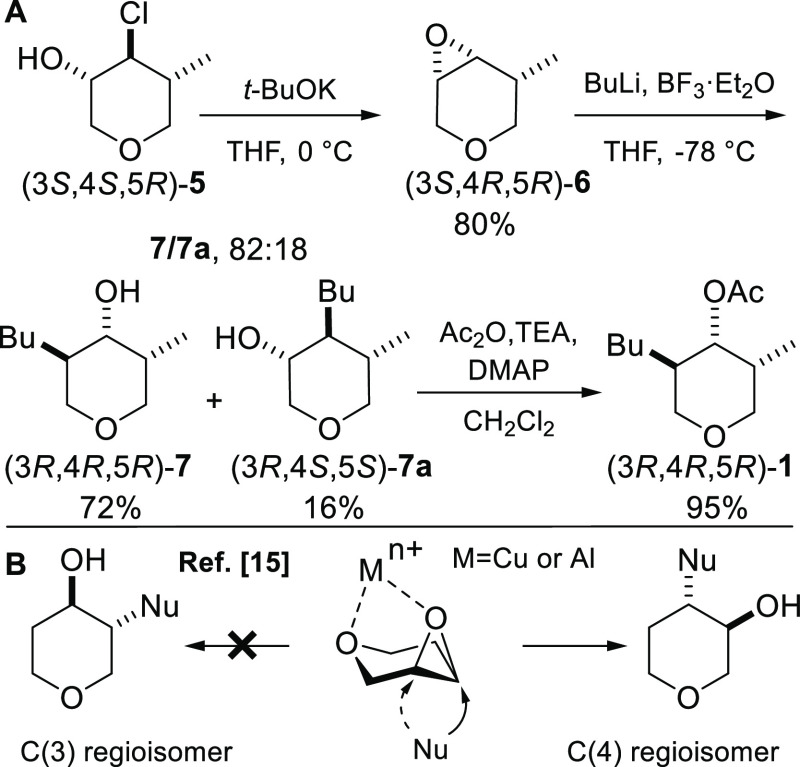
(A) Synthesis of Jessemal; (B) C(4) Regioselective Ring-Opening of an Unsubstituted Tetrahydropyranyl Epoxide with Carbon Nucleophiles Reported by Crotti et al.

Intrigued by the epoxide ring-opening regioselectivity, we decided to explore other nucleophiles (Scheme 2A). At first we tried the addition of Bu_2_CuLi, prepared in situ following the conditions described by Crotti (THF, 6 equiv *n*-BuLi, and 3 equiv CuI, −50 °C).^15^ But, surprisingly, we obtained more C(3) type regioisomer than the expected C(4) isomer (**7**/**7a**, 88:12 by GC-MS). In addition, together with the two regioisomers (60% yield), we isolated also the acyclic diol (2*R*,3*R*)-**9**. However, the optical purities of **7** and **7a** were very similar to those of the same alcohols obtained in the BF_3_ promoted addition of *n*-BuLi. The formation of **9** was likely due to the unreacted *n*-BuLi (residual of the Bu_2_CuLi preparation). Indeed, the treatment of **6** with *n*-BuLi (2.5 equiv, THF, −78 °C) in absence of Lewis acids gave the open-chain diol **9** in 85% yield.^17^

**Scheme 2:**
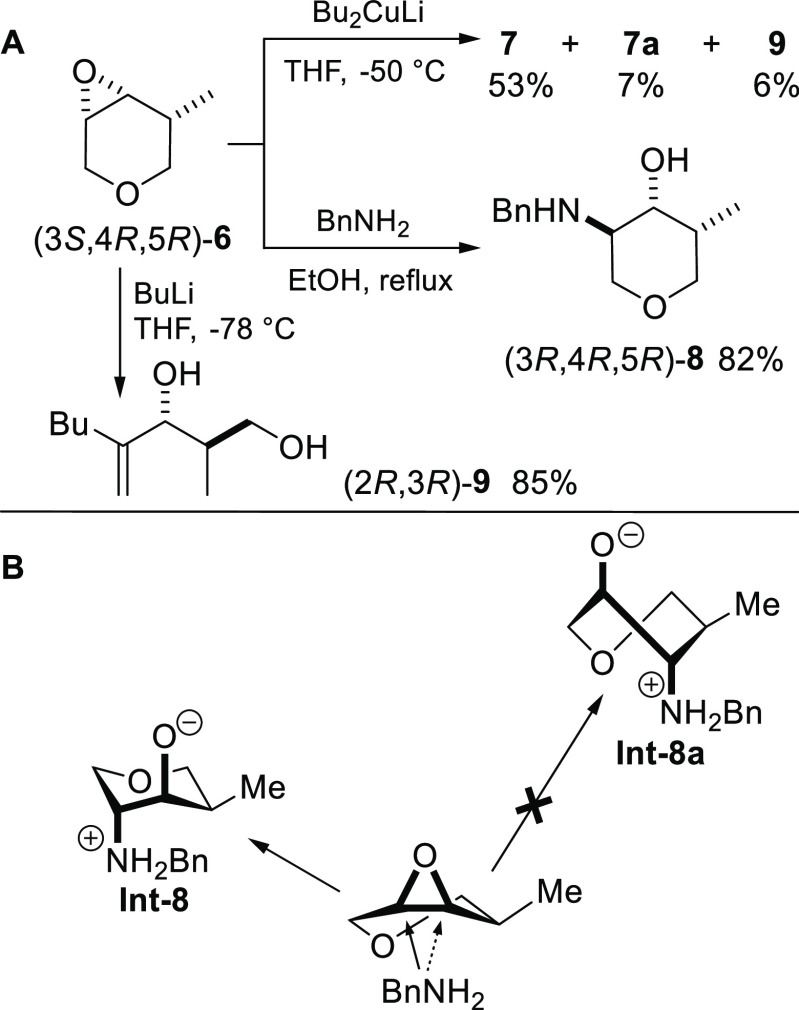
(A) Ring-Opening with Different Nucleophiles; (B) Fürst-Plattner Regiospecificity for the BnNH_2_ Addition

The reaction with BnNH_2_ in refluxing EtOH afforded the amino alcohol (3*R*,4*R*,5,*R*)-**8** in a high yield and with an excellent diastereomeric excess (*de* > 98% by ^1^H NMR). In this case, the epoxide ring-opening attack occurred exclusively at the C(3) position, in full agreement with the Fürst-Plattner rule,^18^ which establishes that the regioselectivity is controlled by the relative stability of the two possible regioisomeric products (being the C(3) type product in a chair-like conformation much more stable than the C(4) regioisomer in the twist-boat conformation, Scheme 2B). Certainly, the partial C(3) regioselectivity observed with the carbon nucleophiles (either with the organocuprate or the organolithium reagents) is not compatible with such rule, and also the explanation of Crotti in the case of BF_3_ catalyzed addition cannot be applied, since the boron atom cannot coordinate the two oxygen atoms of **6**.

Thus, the reaction mechanisms either with *n*-BuLi in the presence of BF_3_ and with BnNH_2_ were examined by density functional theory (DFT) computational chemistry (model chemistry: B3LYP/6-31+g(d,p) and M06-2X-D3/6-31+G(d,p), respectively; all details are in the Supporting Information).^19^

According to the mechanism proposed by Ganem,^16^ the computations showed that **6** together with BF_3_·Et_2_O and *n*-butyl lithium^20^ form two possible regioisomeric reactant states, **RS-C(3)** and **RS-C(4)**, which interconvert rapidly (Figure 2). One of the fluoride atoms plays a key role, since it coordinates the lithium in such a way to orient the incipient butyl nucleophile under the C(3) 0r the C(4) carbon of the epoxide. The energy paths relative to the formation of intermediates **Int-7** and **Int-7a** are shown in Figure 2. Since the reaction is highly exergonic, both reaction trajectories go through a typical reactant-like transition state, i.e., the **TS-C(3)** and **TS-C(4)**. Hence, by applying the Curtin–Hammett equation,^21^ the attack on the C(3) carbon is calculated to be preferred (**7**/**7a**, 72:28 vs experimental 82:18), being the ΔΔ*G*^‡^ = Δ*G*^‡^_**TS-C(4)**_ – Δ*G*^‡^_**TS-C(3)**_ = 0.94 kcal mol^–1^. Concerning the reaction with the amine, the C(3) Fürst-Plattner regiospecificity was confirmed by our computations, since the energy barrier related to the formation of zwitterion **Int-8** (Scheme 2B) was much lower than that of the C(4) type regioisomer (ΔΔ*G*^‡^=4.5 kcal mol^–1^, see Figure S4).

**Figure 2:**
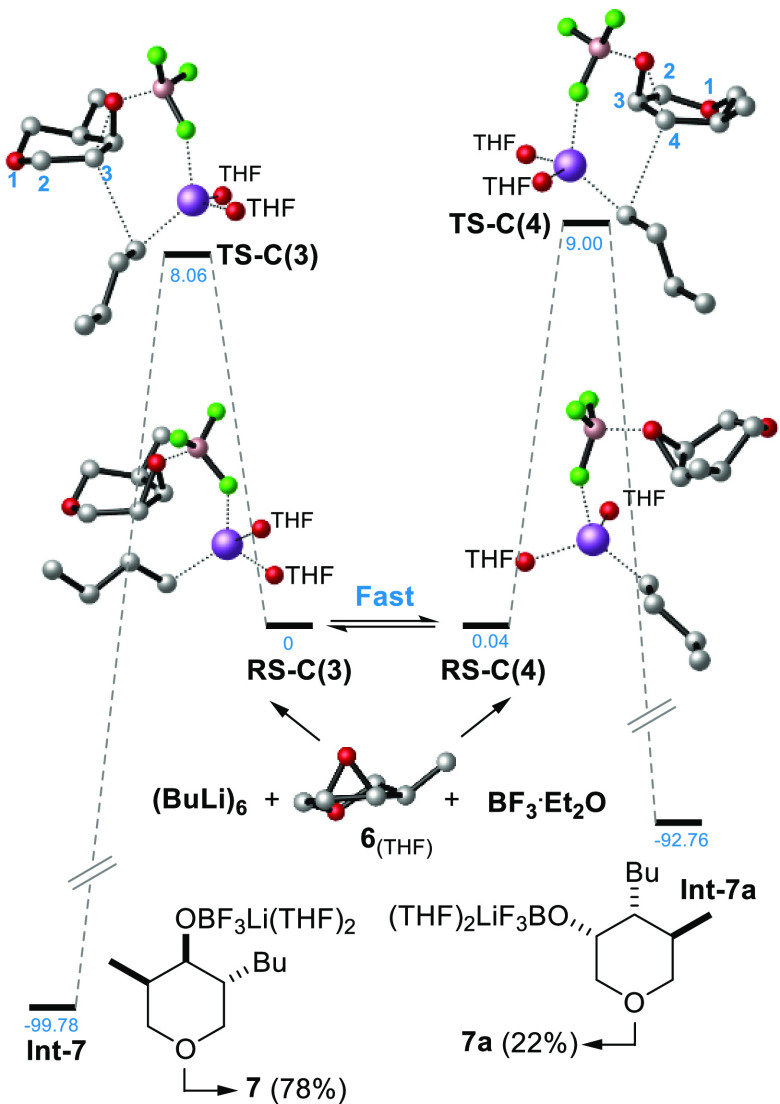
Energy paths for the BF_3_ promoted organolithium ring-opening at 195 K. For clarity, THF structures and H atoms are omitted.

In conclusion we have described the first stereospecific synthesis of the most pleasant stereoisomer of Jessemal fragrance in an overall yield of 18% starting from the commercially available dipropargyl ether. The key step consisted of the very challenging reduction of the α-chloro-tetrasubstituted enone **2**, achieved by combining ER and ADH enzymatic activities in a one-pot cascade process. Lastly, we tested the epoxide ring-opening of **6** with different nucleophiles. Noteworthy, we found that for this kind of substrate, the regioselectivity with carbon nucleophiles such as the organocuprates and organolithium reagents was different to that reported for the unsubstituted tetrahydropyranyl 3,4-epoxide homologue,^15^ broadening the knowledge of epoxide reactivity.

## Supporting Information Available

Preparation and characterization of products, extensive enzymatic screenings, optimization of individual and multienzymatic protocols; Copies of ^1^H and ^13^C NMR spectra; MS and single crystal diffraction data, energies and geometries of computed structures (PDF)
FAIR data, including the primary NMR FID files, for compounds (2*S*,4*S*,5*R*)-**5**, (3*R*,4*S*,5*R*)-**5**, **1**, **2**, **3**, **4**, **6**, **7**, **7a**, **8**, **9**, **10** (ZIP)

## Accession Codes

CCDC 2122799 contains the supplementary crystallographic data for this paper. These data can be obtained free of charge via www.ccdc.cam.ac.uk/data_request/cif, or by emailing data_request@ccdc.cam.ac.uk, or by contacting The Cambridge Crystallographic Data Centre, 12 Union Road, Cambridge CB2 1EZ, UK; fax: +44 1223 336033.

## References

[ref1] aBentley R. The Nose as a Stereochemist. Enantiomers and Odor. Chem. Rev. 2006, 106, 4099–4112. 10.1021/cr050049t. 16967929

[ref2] For an example of odorless stereoisomer, see: Brenna E.; Fuganti C.; Gatti F. G.; Malpezzi L.; Serra S. Synthesis and olfactory evaluation of all stereoisomers of the fragrance Nectaryl®. Tetrahedron: Asymmetry 2008, 19, 800–807. 10.1016/j.tetasy.2008.03.011.

[ref3] Zhang X.; Jing Y.; Ma L.; Zhou J.; Fang X.; Zhang X.; Yu Y. Occurrence and transport of synthetic musks in paired maternal blood, umbilical cord blood, and breast milk. Int. J. Hyg. Environ. Health 2015, 218, 99–106. 10.1016/j.ijheh.2014.08.005. 25256814

[ref4] Abate A.; Brenna E.; Fronza G.; Fuganti C.; Gatti F. G.; Maroncelli S. Enzyme-Mediated Preparation of the Enantiomerically Enriched Isomers of the Odorous Tetrahydropyranyl Acetates Jasmal® and Jessemal^®^, and Their Olfactory Evaluation. Chem. Biodiversity 2006, 3, 677–694. 10.1002/cbdv.200690070. 17193301

[ref5] Venturi S.; Brenna E.; Colombo D.; Fraaije M. W.; Gatti F. G.; Macchi P.; Monti D.; Trajkovic M.; Zamboni E. MultienzymaticStereoselective Reduction of Tetrasubstituted Cyclic Enones to Halohydrins with Three Contiguous Stereogenic Centers. ACS Catal. 2020, 10, 13050–13057. 10.1021/acscatal.0c04097.

[ref6] An C.; Shaw M. H.; Tharp A.; Verma D.; Li H.; Wang H.; Wang X. Enantioselective Enzymatic Reduction of Acrylic Acids. Org. Lett. 2020, 22, 8320–8325. 10.1021/acs.orglett.0c02959. 33048553

[ref7] aToogood H. S.; Scrutton N. S. Discovery, Characterization, Engineering, and Applications of Ene-Reductases for Industrial Biocatalysis. ACS Catal. 2018, 8, 3532–3549. 10.1021/acscatal.8b00624. 31157123 PMC6542678

[ref8] aBrenna E.; Malpezzi L.; Monti D.; Gatti F. G.; Parmeggiani F.; Sacchetti A. Synthesis of Robalzotan, Ebalzotan, and Rotigotine Precursors via the StereoselectiveMultienzymatic Cascade Reduction of α,β-Unsaturated Aldehydes. J. Org. Chem. 2013, 78, 4811–4822. 10.1021/jo4003097. 23611252

[ref9] Skinnemoen K.; Undheim K.; Ullenius C.; Kylin A.; Glaumann H. Synthesis of 2H-Pyran-3-(6H)-ones. Acta Chem. Scand. 1980, 34b, 295–297. 10.3891/acta.chem.scand.34b-0295.

[ref10] Parmeggiani F.; Brenna E.; Colombo D.; Gatti F. G.; Tentori F.; Tessaro D. A Study in Yellow: Investigations in the Stereoselectivity of Ene-Reductases. ChemBioChem 2022, 23, e202100445 10.1002/cbic.202100445. 34586700 PMC9292831

[ref11] Mathew S.; Trajkovic M.; Kumar H.; Nguyen Q.-T.; Fraaije M. W. Enantio- and RegioselectiveEne-Reductions using F_420_H_2_-Dependent Enzymes. Chem. Commun. 2018, 54, 11208–11211. 10.1039/C8CC04449J. 30230493

[ref12] Bechtold M.; Brenna E.; Femmer C.; Gatti F. G.; Panke S.; Parmeggiani F.; Sacchetti A. Biotechnological Development of a Practical Synthesis of Ethyl (*S*)-2-Ethoxy-3-(*p*-methoxyphenyl)propanoate (EEHP): Over 100-Fold Productivity Increase from Yeast Whole Cells to Recombinant Isolated Enzymes. Org. Process Res. Dev 2012, 16, 269–276. 10.1021/op200085k.

[ref13] aNguyen Q.-T.; Trinco G.; Binda C.; Mattevi A.; Fraaije M. W. Discovery and Characterization of an F_420_-Dependent Glucose-6-Phosphate Dehydrogenase (Rh-FGD1) from *Rhodococcus jostii* RHA1. Appl. Microbiol. Biotechnol. 2017, 101, 2831–2842. 10.1007/s00253-016-8038-y. 27966048 PMC5352752

[ref14] Seebach D.; Prelog V. The Unambiguous Specification of the Steric Course of Asymmetric Syntheses. Angew. Chem., Int. Ed. Engl. 1982, 21, 654–660. 10.1002/anie.198206541.

[ref15] Chini M.; Crotti P.; Gardelli C.; Macchia F. Regiochemical control of the ring opening of 1,2-epoxides by means of chelating processes. 6. Opening reactions of 3,4-epoxytetrahydropyran. Tetrahedron 1994, 50, 1261–1274. 10.1016/S0040-4020(01)80836-X.

[ref16] Eis M. J.; Wrobel J. E.; Ganem B. Mechanism and synthetic utility of boron trifluoro etherate promoted organolithium additions. J. Am. Chem. Soc. 1984, 106, 3693–3694. 10.1021/ja00324a060.

[ref17] Royals E. E.; Leffingwell J. C. Reactions of the Limonene 1,2-Oxides. I. The Stereospecific Reactions of the (+)-*cis*- and (+)-*trans*-Limonene 1,2-Oxides. J. Org. Chem. 1966, 31, 1937–1944. 10.1021/jo01344a062.

[ref18] Hodgson D. M.; Stent M. A. H.; Wilson F. X. Organolithium-induced synthesis of unsaturated diols from epoxides of dihydrofurans and dihydropyrans. Synthesis 2002, 10, 1445–1453. 10.1055/s-2002-33112.

[ref19] See the references in the SI.

[ref20] Kottke T.; Stalke D. Structures of Classical Reagents in Chemical Synthesis: (*n*BuLi)_6_, (*t*BuLi)_4_, and the Metastable (*t*BuLi·Et_2_O). Angew. Chem., Int. Ed. 1993, 32, 580–582. 10.1002/anie.199305801.

[ref21] Seeman J. I. Effect of conformational change on reactivity in organic chemistry. Evaluations, applications, and extensions of Curtin-Hammett Winstein-Holness kinetics. Chem. Rev. 1983, 83, 83–131. 10.1021/cr00054a001.

