# Supplementary material for: Chemoenzymatic Synthesis of the Most Pleasant Stereoisomer of Jessemal
Source: J Org Chem. 2022 Apr 20;87(9):6499–503. doi: 10.1021/acs.joc.2c00427 (PMC9087343; doi:10.1021/acs.joc.2c00427)
Supplement: Supplementary file 1 — jo2c00427_si_001.pdf [file jo2c00427_si_001.pdf]

Chemoenzymatic Synthesis of the Most Pleasant Stereoisomer of Jessemal®

Silvia Venturi,[†] Milos Trajkovic,[‡] Danilo Colombo,[†] Elisabetta Brenna,[†] Marco W. Fraaije,[‡] Francesco G. Gatti,[*,†] Piero Macchi,[†] and Emilio Zamboni[†]

†Dipartimento di Chimica, Materiali ed Ingegneria Chimica "G. Natta", Politecnico di Milano, P.zza Leonardo da Vinci 32, 20133 Milano, Italy

‡Molecular Enzymology Group, University of Groningen, Nijenborgh 4, 9747 AG Groningen, The Netherlands

*Corresponding author.

Address: Dipartimento di Chimica, Materiali ed Ingegneria Chimica "G: Natta", Politecnico di Milano . P.zza Leonardo da Vinci, 32, 20133, Milano, Italy. Telephone: +39 02 23993072. Fax: +39 02 23993080. E-mail: francesco.gatti@polomi.it

Contents

General Information S3

Enzymes and Strains S3

Synthesis of 4-Chloro-5-methyl-2*H*-pyran-3(6*H*)-one (4) S4
1,1'-Oxybis(propan-2-one) (**10**) S4
5-Methyl-2*H*-pyran-3(6*H*)-one (**2**) S4
1-Methyl-3,7-dioxabicyclo[4.1.0]heptan-5-one (**3**) S5
4-Chloro-5-methyl-2*H*-pyran-3(6*H*)-one (**4**) S5

Reduction mechanism and possible binding modes of the substrate into the ER catalytic site S6
Figure S1. Reduction mechanism and possible binding modes of 3-methylcyclohex-2-en-1-one into the ER catalytic site S6

Screening of the FDRs mediated reductions S7
Table S1. FDRs catalyzed reduction of **4**. S7

Screening of the OYEs mediated reductions S7
Table S2. OYEs catalyzed reduction of **4** S7

Screening of the FDR+ADH tandem reduction of 4 (one pot process) S8
Table S3. FDRs+ADH one pot reduction of **4** S8

Screening of the OYE2+ADH tandem reduction of 4 (cascade process) S8
Table S4. OYE2+ADH cascade reduction of **4**. S8

General procedure for the FDR-Mha+ADH tandem reduction of 4 on a preparative scale S9
(3*R*,4*S*,5*R*)-4-Chloro-5-methyltetrahydro-2*H*-pyran-3-ol [(3*R*,4*S*,5*R*)-**5**] S9
(3*S*,4*S*,5*R*)-4-Chloro-5-methyltetrahydro-2*H*-pyran-3-ol [(3*S*,4*S*,5*R*)-**5**] S9

General procedure for the OYE2+ADH tandem reduction of 2 on a preparative scale S10
(3*S*,4*R*,5*S*)-4-Chloro-5-methyltetrahydro-2*H*-pyran-3-ol [(3*S*,4*R*,5*S*)-**5**] S10
(3*R*,4*R*,5*S*)-4-Chloro-5-methyltetrahydro-2*H*-pyran-3-ol [(3*R*,4*R*,5*S*)-**5**] S10

Determination of the relative stereochemical configuration of Chlorohydrins 5 .... S11
Figure S2. The ^{1}H-NMR spectra expansion relative to the H(4) signal together with the conformational analysis .S11

Prelog-Seebach stereochemical specifications of the ADH catalyzed reduction of saturated ketone intermediate S12
Figure S3. ADH reduction of ketone intermediate applying the Prelog-Seebach stereochemical specifications S12

(3*S*,4*R*,5*R*)-5-Methyl-3,7-dioxabicyclo[4.1.0]heptane (6) S13

Procedure for the ring-opening with BuLi in presence of $BF_3 \cdot Et_2O$ S13
(3*R*,4*R*,5*R*)-3-Butyl-5-methyltetrahydro-2*H*-pyran-4-ol (**7**) S13
(3*R*,4*S*,5*S*)-4-Butyl-5-methyltetrahydro-2*H*-pyran-3-ol (**7a**) S13

Procedure for the ring-opening with Bu_2CuLi S14

(3*R*,4*R*,5*R*)-3-Butyl-5-methyltetrahydro-2*H*-pyran-4-yl acetate (1) S14

(3*R*,4*R*,5*R*)-3-(Benzylamino)-5-methyltetrahydro-2*H*-pyran-4-ol (8) S14

(2*R*,3*R*)-2-Methyl-4-methyleneoctane-1,3-diol (9) S15

DFT calculations S16
Table S5. Energies of ring-opening with $BnNH_2$: B3LYP/6-31+G(d,p), SCRF=PCM (solvent= EtOH). S16
Ergodic diagram of the ring-opening with $BnNH_2$ in EtOH S16
Figure S4. (a) DFT-computed energy profiles of the C(3) and C4) ring-opening of **6** in the half-chair conformation with $BnNH_2$ S16
Table S6. Energies of the ring-opening with BuLi in presence of $BF_3 \cdot Et_2O$ S22
Thermochemistry of the reactant state formation [RS-C(3) and RS-C(4)] at 195 K S22

Single Crystal X-ray diffraction S35
Table S7. Crystal data and structure refinement for **5**. S35
Table S8. Atomic coordinates (x 10^4) and equivalent isotropic displacement parameters (Å^{2}x 10^3) for compound **5**. S36
Table S9. Bond lengths [Å] and angles [°] for compound **5**. S36

Table S10. Anisotropic displacement parameters (Å^{2}x 10^3) for compound **5** S37
Figure S5. ORTEP Plot of compound **5** as determined from the X-ray diffraction experiment S38
Copies of ^{1}H and ^{13}C{^{1}H} NMR spectra **S39**
References **S56**

General Information

Chemicals and solvents were purchased from suppliers and used without further purification, while, where required, the solvents were dried over molecular sieves (4 Å). ^{1}H and ^{13}C NMR spectra were recorded on a 400 MHz spectrometer at room temperature, using TMS as an internal standard for ^{1}H and $CDCl_3$ for ^{13}C; chemical shifts δ are expressed in ppm relative to the TMS reference; all ^{13}C spectra are proton decoupled ($^{13}C\{^1H\}$).
High-resolution MS spectra were recorded with a Q-TOF mass spectrometer, equipped with an ESI source. The GC-MS analyses of all compounds were performed on a column with a low polarity stationary phase (30 m x 0.25 mm x 0.25 µm). Program temperature: 60 °C (1 min)/6 °C min^{-1}/150 °C (1 min)/12 °C min^{-1}/280 °C (5 min). TLC analyses were performed on precoated silica gel 60 F_{254} plates, and spots were visualized either by UV light (254 nm) or by spraying with phosphomolybdic acid reagent. All chromatographic separations were carried out on silica gel columns (230-400 mesh). Optical rotations were determined on a digital automatic polarimeter at 589 nm (sodium D line) at 25 °C and are given in deg cm^3 g^{-1} dm^{-1}.

Enzymes and Strains

OYE1 (UniProtKB accession number Q02899) from *Saccharomyces pastorianus*, OYE2 (UniProtKB accession number Q03558) and OYE3 (UniProtKB accession number P41816) from *Saccaromyces cerevisiae*, FDR-Mha from *Mycobacterium hassicum*, FDR-Rh1 and FDR-Rh2 from *Rhodococcus jostii*,[1] GDH from *Bacillus megaterium*[2] (UniProtKB accession D5DB49) and FGD from *Rhodococcus jostii*[3] were overexpressed in *E. coli* BL21 (DE3) strains harboring a specific plasmid, according to standard molecular biology techniques.[4] Protein concentrations were determined according to the Bradford test, using Bovine Serum Albumin (BSA) as a standard. EVO270, EVO440 from an unspecified source were purchased from evoxx GmbH in the form of freeze dried powder and used without further purifications. For EVO270 the activity is 15u/mg, test carried out on acetophenone as standard substrate. For EVO440 the activity is 1 u/mg, test carried out on cyclohexanone as standard substrate.

Synthesis of 4-Chloro-5-methyl-2*H*-pyran-3(6*H*)-one (4)

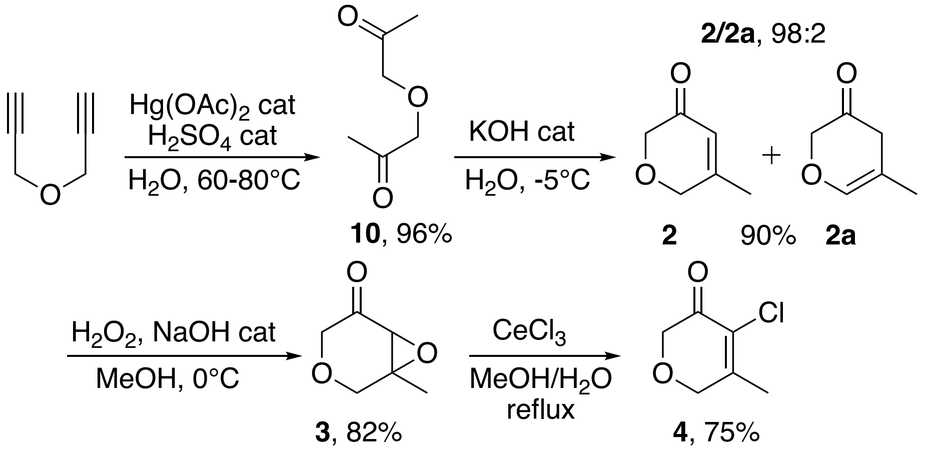

Scheme S1. Synthesis of substrate **4**.

The synthesis of **4** was accomplished in four steps starting from the commercially available dipropargyl ether modifying a known procedure,[5] and it is shown in Scheme S1. However, the alkyne hydration was catalysed with $Hg(OAc)_2$ (7% mol/mol) instead of the typical $HgSO_4$, allowing to achieve the diketone **10** in an almost quantitative yield (96% *vs* 57%). The next step was the base-catalysed intramolecular aldolic condensation, even in this case, the reported procedure was modified: KOH (2 M, 5.2 eq) was used instead of NaOH as Brønsted base. Indeed, by doing this, the detrimental formation of by-product **2a**, an isomer of **2**, was minimal (<2% by GC-MS). In addition, the reaction was conducted at lower temperature (-5°C *vs* rt), in such a way to limit the auto-condensation of **2** and improving significantly the yield (90% *vs* 64%). Remarkably, the synthesis is a column chromatography-free procedure.

1,1'-Oxybis(propan-2-one) (10)

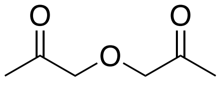

10

The commercially available dipropargyl ether (0.16 mol, 15.0 g) was added dropwise (over 1 h) to a stirred mixture of $Hg(OAc)_2$ (11 mmol, 3.5 g), and H_2SO_4 (4 mL, 98%) in H_2O (100 mL) at 60 °C (the temperature was kept constant by means of an oil bath). After 2 h at 60 °C the temperature was increased to 80 °C and the stirring continued for 2 h. The reaction mixture was extracted with $CHCl_3$ (30 mL x 10). The organic layer was dried over Na_2SO_4 and carefully concentrated under *vacuum* to afford **10**.[5]
Yield 96% (20.0 g) as a colorless oil; tr=7.61 min, 96% purity by GC-MS; ^{1}H NMR ($CDCl_3$, 400 MHz): δ=4.16 (s, 4H), 2.17 (s, 6H); ^{13}C{^{1}H} NMR ($CDCl_3$, 101 MHz): δ=205.70, 76.37, 26.37;[5] GC-MS: *m/z* (%): 130 (M^+, 5), 100 (12), 87 (65), 57 (100).

5-Methyl-2*H*-pyran-3(6*H*)-one (2)

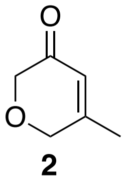

2

To a stirred solution of **10** (0.15 mol, 20.0 g) in H_2O (400 mL), KOH (400 mL, 2 M) was added dropwise at -5 °C. The cooling bath was removed after 15 min and the mixture allowed to reach room temperature. The reaction was extracted with $CHCl_3$ (50 mL x 15). The organic layer was dried over Na_2SO_4 and concentrated under *vacuum* affording **2**.[5]
Yield 88% (15.0 g) as a yellow oil; tr=6.95 min, 92% purity by GC-MS; ^{1}H NMR ($CDCl_3$, 400 MHz): δ=6.01 (m, *J*=1.5 Hz, 1H), 4.24 (dd, *J*=1.8, 0.9 Hz, 2H), 4.08 (d, *J*=0.7 Hz, 2H), 1,94 (q, *J*=0.9 Hz, 3H); ^{13}C{^{1}H} NMR ($CDCl_3$, 101 MHz): δ=194.44, 160.57, 124.11, 71.40, 68.37, 19.71;[5] GC-MS: *m/z* (%): 112 (M^+, 29), 82 (100), 67 (10), 54 (35).

1-Methyl-3,7-dioxabicyclo[4.1.0]heptan-5-one (3)

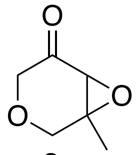

3

To a stirred solution of **2** (0.13 mol, 15.0 g) in MeOH (140 mL), H_2O_2 (35 mL, 35% w/w) was added at 0 °C, followed by the dropwise addition of a NaOH solution (2 mL, 2.5 M). The cooling bath was removed after 3 h and the reaction mixture was poured into cold brine (sat., 100 mL). The crude product was extracted with CH_2Cl_2 (50 mL x 5). The organic layer was dried over Na_2SO_4 and concentrated under *vacuum* affording **3**.[6]
Yield 82% (13.7 g) as a yellow oil; t_r=7.19 min, 95% purity by GC-MS; ^{1}H NMR ($CDCl_3$, 400 MHz): δ=4.25 (d, *J*=18.2 Hz, 1H), 4.00 (d, *J*=13.1 Hz, 1H), 3.84-3.67 (m, 2H), 3.24 (s, 1H), 1.43 (s, 3H); $^{13}C\{^1H\}$ NMR ($CDCl_3$, 101 MHz): δ=202.51, 71.78, 66.12, 60.25, 59.91, 17.34; GC-MS: *m/z* (%): 128 (M^+, 30), 99 (20), 82 (15), 71 (100), 58 (25); HRMS (ESI) calcd for $C_6H_8NaO_3{}^+$ $[M + Na]^+$ 151.0366, found 151.0368.

4-Chloro-5-methyl-2*H*-pyran-3(6*H*)-one (4)

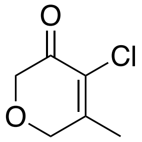

4

The epoxide **3** (0.08 mol, 10.0 g) was dissolved in MeOH/H_2O (400 mL, 3:1) and $CeCl_3$ (0.09 mol, 35.0 g) was added. The reaction mixture was refluxed for 16 h (the temperature was kept constant by means of an oil bath). The mixture was filtered on a pad of celite, which was washed with CH_2Cl_2 (50 mL x 2) and then was added H_2O (50 mL). The aqueous layer was extracted with CH_2Cl_2 (150 mL x 3). The organic layer was dried over Na_2SO_4 and concentrated under *vacuum* to give the crude product, which was purified by crystallization in pentane/Et_2O (8:2) at low temperature (-10 °C) affording **4**.[7]
Yield 75% (8.6 g) as a yellow oil; t_r=11.91 min, 98% purity by GC-MS; ^{1}H NMR ($CDCl_3$, 400 MHz): δ=4.41 (s, 2H), 4.27 (s, 2H), 2.05 (s, 3H); $^{13}C\{^1H\}$ NMR ($CDCl_3$, 101 MHz): δ=186.82, 154.92, 126.58, 72.48, 70.15, 17.41; GC-MS: m/z (%): 146 (M^+, 10), 116 (45), 111 (90), 88 (32), 53 (100); HRMS (ESI) calcd for $C_6H_7ClNaO_2{}^+$ $[M + Na]^+$ 169.0027, found 169.0025.

Reduction mechanism and possible binding modes of the substrate into the ER catalytic site

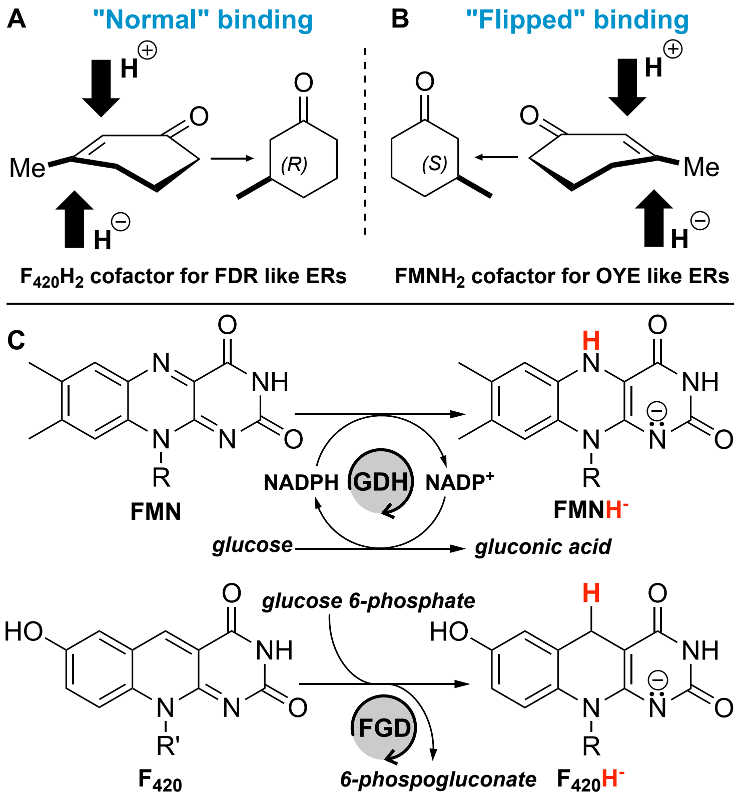

Figure S1. (A) "normal" binding for FDR-type ERs and (B) "flipped" binding for OYE-type ERs. (C) Cofactor recycling systems.

Screening of the FDRs mediated reductions

A solution of **4** in DMSO (33 µL, 30 mM) was added to a *tris*·HCl buffer (967 µL, 50 mM, pH 8.0) containing 10 mM glucose-6-phosphate, 20 µM of F_{420}, 0.1 µM of FGD-Rha1 and 25 µM FDR according to Table S1. The mixture was incubated for 3 h in an orbital shaker (150 rpm, 24 °C). The solution was extracted with EtOAc (250 µL x 2), centrifuging after each extraction (15000 *g*, 1.5 min). The combined organic solution was dried over Na_2SO_4, and analyzed by GC-MS. In Table S1 we report conversion and the percentage of the *trans*- and of the *cis*- ketones.
Cis-ketone: tr=8.19 min, GC-MS: *m/z* (%): 148 (M, 20), 90 (60), 75 (10), 55 (100).
Trans-ketone: tr=8.61 min, GC-MS: *m/z* (%): 148 (M, 20), 90 (60), 75 (15), 55 (100).

Table S1. FDRs catalyzed reduction of **4**.

**ER**	**Conversion** (%)[a]	*trans* (%)[a]	*cis* (%)[a]
FDR-Rha1	99	81	19
FDR-Rha2	99	79	21
FDR-Mha	99	78	22

a) By GC-MS

Screening of the OYEs mediated reductions

A solution of **4** in DMSO (10 µL, 500 mM) was added to a KP_i buffer solution (1.0 mL, 50 mM, pH 7.0) containing glucose (3.6 mg, 20 µmol), $NADP^+$ (0.1 µmol), GDH (5 U), and the OYE (80-120 µg mL^{-1}) according to Table S2. The mixture was incubated for 24 h in an orbital shaker (150 rpm, 30 °C). The solution was extracted with EtOAc (250 µL x 2), centrifuging after each extraction (15000 *g*, 1.5 min), and the combined organic solution was dried over Na_2SO_4 and analyzed by GC-MS.
Cis-ketone: tr=8.19 min, GC-MS: *m/z* (%): 148 (M, 20), 90 (60), 75 (10), 55 (100).
Trans-ketone: tr=8.61 min, GC-MS: *m/z* (%): 148 (M, 20), 90 (60), 75 (15), 55 (100).

Table S2. OYEs catalyzed reduction of **4**.

**ER**	**Conversion**[a] (%)	*trans*[a] (%)	*cis*[a] (%)
OYE1	95	75	25
OYE2	99	82	18
OYE3	96	78	22

a) By GC-MS

Screening of the FDR+ADH tandem reduction of 4 (one pot process)

A solution of **4** in DMSO (33 µL, 30 mM) was added to a *tris*·HCl buffer (967 µL, 50 mM, pH 8.0) containing 10 mM glucose-6-phosphate, 20 µM of F_{420}, 0.1 µM of FGD-Rha1 and 25 µM of FDR according to Table S3. The mixture was incubated for 3 h in an orbital shaker (150 rpm, 24 °C). After that pH was adjusted to 7.0 with 500 mM KH_2PO_4 (~100 µL) and were added 10 µM of $NADP^+$, ADH (200 µg), 10 µM of PTDH and 10 mM of sodium phosphite (final volume is 1.2 mL). The mixture was incubated for 24 h in an orbital shaker (150 rpm, 30 °C). The solution was extracted with EtOAc (2 x 250 µL), centrifuging after each extraction (15000 *g*, 1.5 min), and the combined organic solution was dried over Na_2SO_4.

Table S3. FDRs+ADH one pot reduction of **4**.

ER+ADH	Conversion (%)[a]	*trans,trans*-**5** (%)[a]	*cis,trans*-**5** (%)[a]
FDR-Rha1+EVO270	99	21	79
FDR-Rha1+EVO440	99	95	5
FDR-Rha2+EVO270	99	22	78
FDR-Rha2+EVO440	99	93	7
FDR-Mha+EVO270	99	20	80
FDR-Mha+EVO440	99	95	5

a) By GC-MS

Screening of the OYE2+ADH tandem reduction of 4 (cascade process)

A solution of **4** in DMSO (10 µL, 500 mM) was added to a KP_i buffer solution (1.0 mL, 50 mM, pH 7.0) containing glucose (3.6 mg, 20 µmol), $NADP^+$ (0.1 µmol), GDH (5 U), OYE2 (80-120 µg mL^{-1}) and an ADH (200 µg), according to Table S4. The mixture was incubated for 24 h in an orbital shaker (150 rpm, 30 °C). The solution was extracted with EtOAc (2 x 250 µL), centrifuging after each extraction (15000 *g*, 1.5 min), and the combined organic solutions were dried over Na_2SO_4.

Table S4. OYE2+ADH cascade reduction of **4**.

ER+ADH	Conversion (%)[a]	*trans,trans*-**5** (%)[a]	*cis,trans*-**5** (%)[a]
OYE2+EVO270	99	11	89
OYE2+EVO440	99	92	8

a) By GC-MS

The diastereomeric excesses of *trans*-ketone (Table S1 and S2) obtained in the screening with the ERs are always lower than those achieved either in the cascade process (OYE+ADH, Table S4) and in the one pot reduction (1)FDR+2)ADH, Table S3). We think that during the bioreduction of C=C double bond the *trans* saturated ketone undergo to a epimerization of the stereogenic center at α position affording the *cis* diastereoisomer. This detrimental side reaction can be mitigated when the biotransformation is carried out in presence of ADH, because as soon the *trans* ketone is formed it is promptly reduced to the not epimerizable alcohol (the beneficial effect of cascade process is generally higher of one pot process).

General procedure for the FDR-Mha+ADH tandem reduction of 4 on a preparative scale

A solution **4** (440 mg, 3.0 mmol) in DMSO (5 mL) was added to *tris*·HCl buffer (500 mL, 50 mM, pH 8.0) containing glucose-6-phosphate (10 mM), F_{420} (20 μM), FGD-Rha1 (0.1 μM) and FDR-Mha (25 μM). The mixture was incubated for 10 h in an orbital shaker (150 rpm, 24 °C). After the pH was adjusted to 7.0 with KH_2PO_4 (500 mM), then were added $NADP^+$ (7.5 mg), ADH (EVO270 or EVO440, 30-40 mg), PTDH (10 mM) and $Na_2HPO_3(H_2O)_5$ (1.1 g). The mixture was incubated for 24 h in an orbital shaker (150 rpm, 30 °C). To the reaction mixture was added NaCl (3.0 g), then it was extracted with Et_2O (6 x 30 mL), dried over Na_2SO_4. Since the product is very volatile, the solvent was removed by distillation without *vacuum*, to give the crude material.

(3*R*,4*S*,5*R*)-4-Chloro-5-methyltetrahydro-2*H*-pyran-3-ol [(3*R*,4*S*,5*R*)-5]

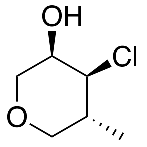

(3*R*,4*S*,5*R*)-**5**

With EVO270. The product was purified through column chromatography (*n*-pentane/Et_2O, 6:4).
Yield 69% (311 mg) as colorless liquid; tr=9.57 min, 99% purity by GC-MS; $[\alpha]_D$=-84.1° (*c* 0.45, $CHCl_3$, 25 °C); ^1H NMR ($CDCl_3$, 400 MHz): δ=4.05 (ddd, *J*=12.2, 2.9, 1.4 Hz, 1H, H (2_{eq})), 3.93 (ddd, *J*=11.7, 4.5, 1.4, 1H, H (6_{eq})), 3.88 (m, *J*=2.7, 1H, H(3)), 3.83 (dd, *J*=10.4, 2.9, 1H, H(4)), 3.51 (dt, *J*=12.2, 1.4 Hz, 1H, H(2_{ax})), 3.07 (dd, *J*=11.7, 10.3 Hz, 1H, H(6_{ax})), 2.40 (dd, *J*=4.9, 1.5 Hz, OH), 2.30 (m, 1H, H(5)), 1.01 (d, *J*=6.7 Hz, 3H, Me); $^{13}C\{^1H\}$ NMR ($CDCl_3$, 101 MHz): δ=72.96, 70.92, 68.57, 68.48, 35.01, 14.75; GC-MS: *m/z* (%):150 (M^+, 18), 97.1 (16), 78 (22), 71 (100), 60 (29), 55 (39); $[\alpha]_D$=-84.1° (*c* 0.45, $CHCl_3$); HRMS (ESI) calcd for $C_6H_{11}ClNaO_2{}^+$ $[M + Na]^+$ 173.0340, found 173.0339.

(3*S*,4*S*,5*R*)-4-Chloro-5-methyltetrahydro-2*H*-pyran-3-ol [(3*S*,4*S*,5*R*)-5]

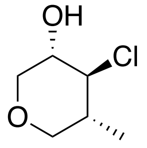

(3*S*,4*S*,5*R*)-**5**

With EVO440. The product was purified by crystallization (*n*-pentane/Et_2O, 9:1) at 0 °C.
Yield 62% (279 mg) as a white crystal; tr=8.20 min, 99% purity by GC-MS; $[\alpha]_D$=-17.3 ° (*c* 0.43, $CHCl_3$, 25 °C); ^1H NMR ($CDCl_3$, 400 MHz): δ=4.10 (ddd, *J*=11.3, 5.2, 1.3 Hz, 1H, H(2_{eq})), 3.94-3.83 (m, 1H, H(6_{eq})), 3.73 (m, 1H, H(3)), 3.49 (dd, *J*=10.8, 9.3 Hz, 1H, H(4)), 3.17 (dd, *J*=11.3, 10.1 Hz, 1H, H(2_{ax})), 3.05 (t, *J*=11.4 Hz, 1H, H(6_{ax})), 2.38 (d, *J*=2.8 Hz, OH), 2.13-1.89 (m, 1H, H(5)), 1.03 (d, *J*=6.6 Hz, 3H, Me); $^{13}C\{^1H\}$ NMR ($CDCl_3$ 101 MHz): δ=73.32, 72.13, 71.77, 71.29, 39.32, 14.04; GC-MS: *m/z* (%):150 (M^+, 18), 97.1 (16), 78 (22), 71 (100), 60 (29), 55 (39); HRMS (ESI) calcd for $C_6H_{11}ClNaO_2{}^+$ $[M + Na]^+$ 173.0340, found 173.0342.

General procedure for the OYE2+ADH tandem reduction of 2 on a preparative scale

A solution of **4** (440 mg, 3.0 mmol) in *i*-PrOH (0.250 mL) was added to a KP_i buffer solution (15 mL, 50 mM, pH 7.0) containing OYE2 (5 mL, 3 mg/mL), GDH (200 U), glucose (4.2 g), $NADP^+$ (15.0 mg) and ADH (EVO270 or EVO440, 30-40 mg). The reaction was monitored by TLC until complete conversion. Eventually, more enzymes were added to increase the conversion. To the reaction mixture was added NaCl (3.0 g), then it was extracted with Et_2O (30 mL x 3) and dried over Na_2SO_4. Since the product is very volatile, the solvent was removed by distillation without *vacuum* affording the crude material.

(3*S*,4*R*,5*S*)-4-Chloro-5-methyltetrahydro-2*H*-pyran-3-ol [(3*S*,4*R*,5*S*)-5]

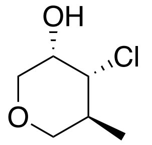

(3*S*,4*R*,5*S*)-**5**

With EVO270. The product was purified through column chromatography (*n*-pentane/Et_2O, 6:4).
Yield 71% (322 mg) as a colorless oil; the NMR spectroscopy data are consistent with its enantiomer; $[\alpha]_D$=+83.8° (*c* 0.53, $CHCl_3$, 25 °C); HRMS (ESI) calcd for $C_6H_{11}ClNaO_2{}^+$ $[M + Na]^+$ 173.0340, found 173.0337.

(3*R*,4*R*,5*S*)-4-Chloro-5-methyltetrahydro-2*H*-pyran-3-ol [(3*R*,4*R*,5*S*)-5]

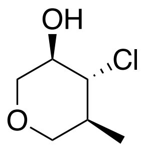

(3*R*,4*R*,5*S*)-**5**

With EVO440. The product was isolated by crystallization (*n*-pentane/Et_2O, 9:1) at 0 °C and a sample was submitted to X-ray diffraction.
Yield 65% (293 mg) as a white crystal; the NMR spectroscopy data are consistent with its enantiomer; $[\alpha]_D$=+16.7 ° (*c* 0.14, $CHCl_3$, 25 °C); HRMS (ESI) calcd for $C_6H_{11}ClNaO_2{}^+$ $[M + Na]^+$ 173.0340, found 173.0341.

Determination of the relative stereochemical configuration of Chlorohydrins 5

The ^{1}H-NMR spectrum of each diastereoisomer of **5** allowed to attribute the relative stereochemical configuration by measuring the coupling constants (J) of the proton signal H(4) (C*H*-Cl). First, all signals were unambiguously assigned by ^{1}H-^{1}H-COSY. In figure S2 we show the expansion of ^{1}H-spectra of (*trans*,*trans*)-**5** and (*cis*,*trans*)-**5** diastereoisomers ($CDCl_3$, 400 MHz). According to the Karplus plot and to the conformational analysis, to the diastereoisomer with J= 9.3 and 10.8 Hz of signal H(4) (δ=3.49 ppm) was assigned the *trans,trans* configuration (Figure S2a). This diastereoisomer adopts mainly the chair-like conformation in which the H(3), H(4) and H(5) hydrogens are all *axial*, and therefore H(4) is coupled with H(2) and H(5) by two typically large J_{anti} coupling constants. While, to the diastereoisomer with J= 10.4 and 2.8 Hz of H(4) signal (δ=3.83 ppm), was assigned the *cis*,*trans* configuration (Figure S2b). Indeed, in the most stable conformer, H(4) is coupled with H(3) by a small J_{guache} coupling constant and with H(5) by a large J_{anti} coupling constant.

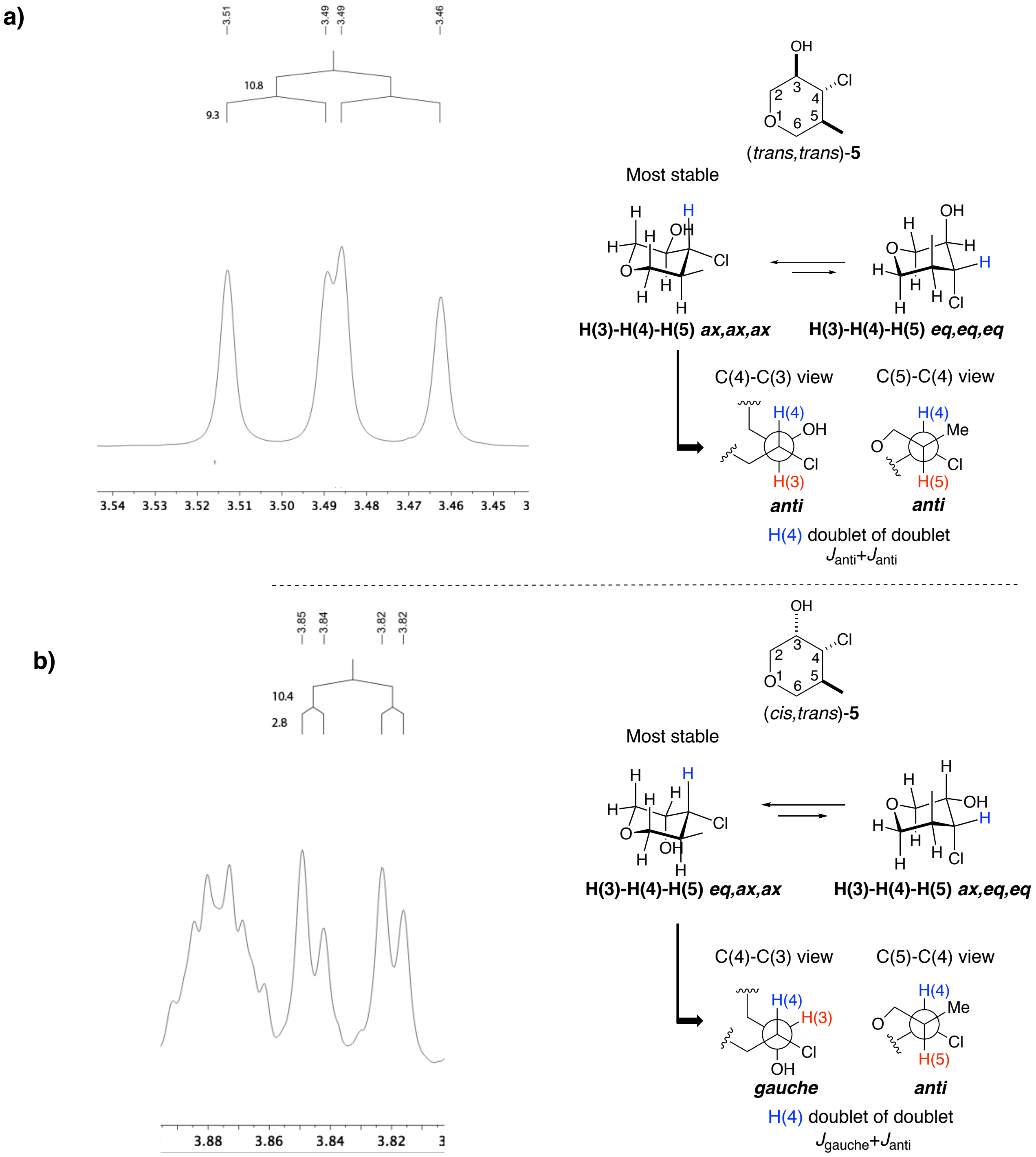

Figure S2. The ^{1}H-NMR spectra expansion relative to the H(4) signal together with the conformational analysis are shown for: a) *trans*,*trans* diastereoisomer; b) *cis*,*trans* diastereoisomer.

Prelog-Seebach stereochemical specifications of the ADH catalyzed reduction of saturated ketone intermediate

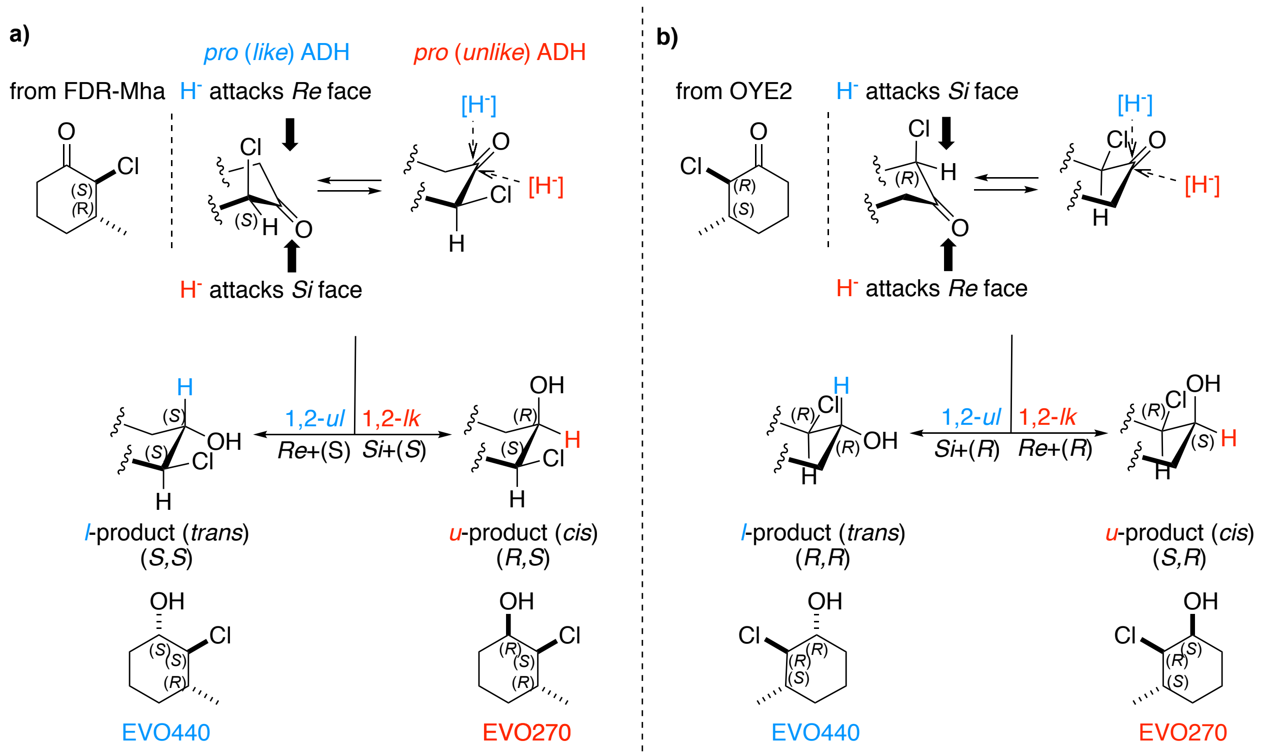

Figure S3. ADH reduction of ketone intermediate applying the Prelog-Seebach stereochemical specifications: a) reduction of ketone from FDR-Mha biotransformation and b) reduction of ketone from OYE2 biotransformation. EVO270 exhibits a *pro-like* stereospecficity, while EVO440 is *pro-unlike*.[8]

(3*S*,4*R*,5*R*)-5-Methyl-3,7-dioxabicyclo[4.1.0]heptane (6)

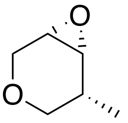

(3*S*,4*R*,5*R*)-**6**

To a stirred solution of (3*S*,4*S*,5*R*)-**5** (1.0 mmol, 150 mg) in THF (5 mL) was added *t*-BuOK (1.3 mmol, 145 mg) at 0 °C. Then, the reaction mixture was left to stir at room temperature until complete consumption of chlorohydrin (checked by TLC). The reaction mixture was quenched with H_2O (2 mL) and filtered on a celite pad, which was washed with Et_2O (10 mL x 3).
The aqueous layer was extracted with Et_2O (10 mL x 3), the combined organic layers was dried over Na_2SO_4 and the solvent was carefully removed at room pressure affording the crude product, which was purified by column chromatography (*n*-pentane/Et_2O, 8:2) to give the epoxide **6**.
Yield 80% (91 mg) as a colorless oil; tr=4.86 min, 97% purity by GC-MS; $[\alpha]_D$<-1° (*c* 0.19, $CHCl_3$, 25 °C); ^{1}H NMR ($CDCl_3$, 400 MHz): δ=4.07 (ddd, *J*=13.4, 3.7, 0.9 Hz, 1H), 3.82 (dt, *J*=13.4, 0.8, 1H), 3.49-3.43 (m, 1H), 3.27 (td, *J*=4.0, 0.7 Hz, 1H), 3.23 (m, 1H), 3.03 (dd, *J*=11.3, 9.8 Hz, 1H), 2.25-2.09 (m, 1H), 1.02 (d, *J*=6.9 Hz, 3H); $^{13}C\{^1H\}$ NMR ($CDCl_3$ 101 MHz): δ=66.75, 65.11, 54.38, 52.36, 29.93, 13.75; GC-MS: *m/z* (%): 114 (M^+, 5), 99 (15), 84 (66), 71 (100), 58 (15), 55 (80), 44 (48), 41 (93); HRMS (ESI) calcd for $C_6H_{10}NaO_2{}^+$ $[M + Na]^+$ 137.0573, found 137.0571.

Procedure for the ring-opening with BuLi in presence of $BF_3{\cdot}Et_2O$

To a flame-dried Schlenk tube equipped with a stir bar, under an atmosphere of N_2, loaded with THF (3 mL), $BF_3{\cdot}Et_2O$ (2.5 mmol, 360 mg) at -78 °C was added a solution of *n*-BuLi in *n*-hexane (1 mL, 2.5 M). Then, a solution of **6** (1.0 mmol, 114 mg) in THF (2 mL) was added dropwise. The reaction mixture was left to stir at -78 °C for 15 minutes, then, it was quenched with NH_4Cl (sat., 10 mL). The aqueous layer was extracted with Et_2O (10 mL x 3), the combined organic layer was washed with brine (15 mL) and dried over Na_2SO_4. The solvent was removed under *vacuum* affording the crude product, which was purified by column chromatography (*n*-hexane/EtOAc, 8:2) giving the alcohols **7** and **7a**.[9]

(3*R*,4*R*,5*R*)-3-Butyl-5-methyltetrahydro-2*H*-pyran-4-ol (7)

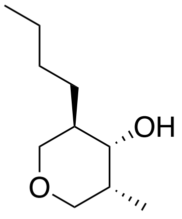

(3*R*,4*R*,5*R*)-**7**

Yield 72% (123 mg) as a colorless oil; tr=14.56 min, 99% purity by GC-MS; $[\alpha]_D$=-74.0° (*c* 0.42, $CHCl_3$, 25 °C); ^{1}H NMR (C_6D_6, 400 MHz): δ=3.87 (dd, *J*=11.3, 3.6 Hz, 1H), 3.55 (dd, *J*=11.1, 6.4 Hz, 1H), 3.35 (dd, *J*=11.2, 3.6 Hz, 1H), 3.19 (dt, *J*=6.0, 2.9 Hz, 1H), 3.13 (dd, *J*=11.4, 6.1 Hz, 1H), 1.67-1.59 (m, 1H), 1.43-1.35 (m, 2H), 1.28-1.05 (m, 5H), 0.9-0.83 (m, 6H); $^{13}C\{^1H\}$ NMR (C_6D_6 101 MHz): δ=73.27, 70.60, 68.49, 40.66, 33.97, 29.87, 29.13, 23.43, 14.25, 11.90;[10] GC-MS: *m/z* (%): 172 (M^+, 5), 154 (43), 139 (13), 131 (22), 111 (35), 97 (100), 89 (65), 69 (65), 55 (78).

(3*R*,4*S*,5*S*)-4-Butyl-5-methyltetrahydro-2*H*-pyran-3-ol (7a)

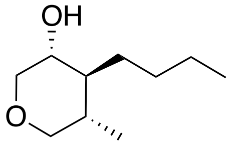

(3*R*,4*S*,5*S*)-**7a**

Yield 16% (27 mg) as a colorless oil; tr=13.79 min, 98% purity by GC-MS; $[\alpha]_D$=-7.6° (*c* 0.42, $CHCl_3$, 25 °C); ^{1}H NMR ($CDCl_3$, 400 MHz): δ=3.97 (ddd, *J*=10.7, 4.9, 0.9 Hz, 1H, H(2_{eq})), 3.75 (dd, *J*=11.2, 4.5 Hz, 1H, H(6_{eq})), 3.55 (td, *J*=10.5,

9.7, 4.7 Hz, 1H, H(3)), 3.06 (dd, *J*=10.7, 10.0 Hz, 1H, H(2_{ax})), 2.97 (t, *J*=11.1 Hz, 1H, H(6_{ax})), 1.72-1.60 (m, 2H, H(5)), 1.42-1.29 (m, 5H + OH), 1.08 (tt, *J*=10.3, 4.0 Hz, 1H, H(4)), 0.99-0.89 (m, 3H, Me), 0.84 (d, *J*=6.7 Hz, 3H, Me); $^{13}C\{^1H\}$ NMR ($CDCl_3$ 101 MHz): δ=74.35, 72.93, 68.97, 49.53, 33.64, 27.82, 27.43, 23.60, 14.21, 14.15; GC-MS: *m/z* (%): 172 (M^+, 5), 154 (8), 97 (100), 85 (40), 69 (65), 57 (100), 41 (87); HRMS (ESI) calcd for $C_{10}H_{20}NaO_2{}^+$ $[M + Na]^+$ 195.1356, found 195.1358.

Procedure for the ring-opening with Bu_2CuLi

To a flame-dried Schlenk tube equipped with a stir bar, under an atmosphere of N_2, was loaded with CuI (3 mmol, 570 mg) and THF (4.5 mL), then, a solution of *n*-BuLi in hexane (2.4 mL, 2.5 M) was added dropwise at -20 °C. After 15 minutes a solution of **6** (1.0 mmol, 114 mg), in THF (1.0 mL), was added dropwise at -50 °C.[11] The reaction mixture was left to reach 0 °C, then, it was quenched with NH_4Cl (sat., 10 mL). The aqueous layer was extracted with Et_2O (10 mL x 3), the combined organic layer was washed with brine (sat., 15 mL) and dried over Na_2SO_4. The solvent was removed under *vacuum* affording the crude product, which was purified by column chromatography (*n*-hexane/EtOAc, 85:15) to give the alcohols **7** and **7a**.
The NMR spectroscopy data are consistent with those of same compounds obtained with the ring-opening promoted by $BF_3{\cdot}Et_2O$, for **7** $[\alpha]_D$=-72.3° (*c* 0.41, $CHCl_3$, 25 °C).

(3*R*,4*R*,5*R*)-3-Butyl-5-methyltetrahydro-2*H*-pyran-4-yl acetate (1)

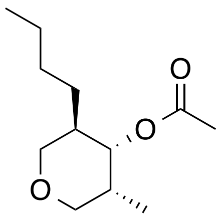

(3*R*,4*R*,5*R*)-**1**

To a solution of **7** (1.0 mmol, 172 mg) in CH_2Cl_2 (2.0 mL) Ac_2O (2.5 mmol, 250 mg), TEA (2.0 mmol, 200 mg) and DMAP (0.08 mmol, 10 mg) were added. The reaction was left to stir at room temperature until complete consumption of **7** (checked by TLC). To the mixture was added NH_4Cl (sat., 5 mL), the aqueous layer was extracted with CH_2Cl_2 (20 mL x 3). The combined organic layer was dried over Na_2SO_4 and the solvent was removed under *vacuum* to give the crude product, which was purified by column chromatography (*n*-hexane/ EtOAc, 9:1) to give the Jessemal®.
Yield 95% (203 mg) as a colorless oil; tr=17.14 min, 99% purity by GC-MS; $[\alpha]_D$=-59.1° (*c* 0.41, $CHCl_3$, 25 °C); ^1H NMR ($CDCl_3$, 400 MHz): δ=4.79 (dd, *J*=6.7, 3.9 Hz, 1H), 3.82 (dd, *J*=11.6, 3.7 Hz, 1H), 3.57 (dd, *J*=11.4, 4.0 Hz, 1H), 3.51 (dd, *J*=11.4, 6.8 Hz, 1H), 3.34 (dd, *J*=11.6, 6.0 Hz, 1H), 2.14-2.06 (m, 4H), 1.83-1.68 (m, 1H), 1.45-1.18 (m, 6H), 0.91-0.87 (m, 6H); $^{13}C\{^1H\}$ NMR ($CDCl_3$ 101 MHz): δ=170.72, 75.43, 70.67, 68.66, 37.63, 31.35, 29.33, 28.75, 22.99, 21.27, 14.09, 12.19;[10] GC-MS: *m/z* (%): 154 (50 $[M – 60]^+$), 111 (60), 97 (100), 82 (30), 69 (10) 55 (45).

(3*R*,4*R*,5*R*)-3-(Benzylamino)-5-methyltetrahydro-2*H*-pyran-4-ol (8)

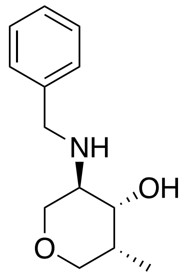

(3*R*,4*R*,5*R*)-**8**

To a stirred solution of **6** (1.0 mmol, 114 mg) in EtOH (2 mL) was added benzylamine (1.0 mmol, 107 mg). The mixture was refluxed for 16 h (the temperature was kept constant by means of an oil bath). Both solvent and the unreacted starting materials were removed under *vacuum* affording the amino alcohol **8** without the need of further purification procedures.

Yield 82% (180 mg) as a white solid; tr=24.08 min, 99% purity by GC-MS; $[\alpha]_D$=-80.3° (*c* 2.16, $CHCl_3$, 25 °C); ^{1}H NMR ($CDCl_3$, 400 MHz): δ=7.31-7.27 (m, 4H), 7.22-7.18 (m, 1H), 3.94 (dd, *J*=11.3, 3.8 Hz, 1H, H(2_{eq})), 3.84 (d, *J*=13.1 Hz, 1H, CH-Ph), 3.70 (d, *J*=13.1 Hz, 1H, CH-Ph), 3.60-3.54 (m, 1H, H(6_{eq})), 3.51 (dd, *J*=7.8, 4.3 Hz, 1H, H(4)), 3.45 (dd, *J*=11.4, 3.2 Hz, 1H, H(6_{ax})), 3.13 (dd, *J*=11.3, 7.7 Hz, 1H, H(2_{ax})), 2.66 (td, *J*=7.7, 3.9 Hz, 1H, H(3)), 2.02 (dtd, *J*=7.3, 4.4, 3.0 Hz, 1H, H(5)), 1.79 (s, OH), 0.95 (d, *J*=7.1 Hz, 3H, Me); ^{13}C{^{1}H} NMR ($CDCl_3$ 101 MHz): δ=140.44, 128.64, 128.16, 127.29, 73.04, 71.13, 68.98, 56.36, 51.72, 33.73, 11.46; GC-MS: *m/z* (%): 221 (M^+, 40), 148 (20), 120 (30), 91 (100), 65 (15); HRMS (ESI) calcd for $C_{13}H_{19}NNaO_2^+$ $[M + Na]^+$ 244.1308, found 244.1312.

(2*R*,3*R*)-2-Methyl-4-methyleneoctane-1,3-diol (9)

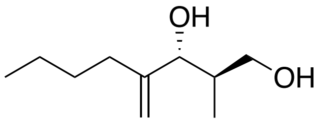

(2*R*,3*R*)-**9**

To a stirred solution of **6** (1.0 mmol, 114 mg) in THF (5 mL), under an atmosphere of N_2, was added dropwise a solution of *n*-BuLi in *n*-hexane (840 µL, 2.5 M) at -78 °C. The mixture was allowed to reach room temperature, then, it was quenched with NH_4Cl (sat., 10 mL). The aqueous layer was extracted with Et_2O (10 mL x 3), the combined organic layer was washed with brine (sat., 15 mL) and dried over Na_2SO_4. The solvent was removed under *vacuum* affording the crude product, which was purified by column chromatography (*n*-hexane/EtOAc, 8:2) to give the diol **9**.[12]
Yield 85% (146 mg) as a yellow oil; tr=16.10 min, 99% purity by GC-MS; $[\alpha]_D$=-4.9° (*c* 1.52, $CHCl_3$, 25 °C); ^{1}H NMR ($CDCl_3$, 400 MHz): δ=5.02 (t, *J*=1.2 Hz, 1H, =CH), 4.91 (q, *J*=1.6 Hz, 1H, =CH), 3.97 (d, *J*=8.0 Hz, 1H, H(3)), 3.73 (dd, *J*=10.8, 3.4 Hz, 1H, H(1)), 3.62 (dd, *J*=10.8, 7.3 Hz, 1H, H(1')), 3.15 (s, OH), 2.82 (s, OH), 2.18-2.05 (m, 1H, H(5)), 2.02-1.84 (m, 2H, H(5')+H(2)), 1.55-1.41 (m, 2H, H(6)), 1.41-1.30 (m, 2H, H(7)), 0.91 (t, *J*=7.3 Hz, 3H, H(8)), 0.81 (d, *J*=7.0 Hz, 3H,Me); ^{13}C{^{1}H} NMR ($CDCl_3$ 101 MHz): δ=150.81, 111.56, 82.69, 67.75, 37.62, 30.44, 30.28, 22.83, 14.13, 14.10; ; GC-MS: *m/z* (%): 172 (M^+, 10), 113 (30), 97 (25), 71 (100), 67 (20), 55 (30); HRMS (ESI) calcd for $C_{10}H_{20}NaO_2^+$ $[M + Na]^+$ 195.1356, found 195.1353.

DFT calculations

Density functional theory (DFT) calculations were performed with Gaussian 16.6.[13] Molecular geometry optimizations and frequency calculations were performed using the B3LYP functional,[14] augmented with Grimme's D3 empirical dispersion term,[15] using the 6-31+G(d,p) basis set[16] and SCRF=PCM (solvent= EtOH) only for the $BnNH_2$ ring-opening.[17] Frequency calculations confirmed the optimized structures as minima (zero imaginary frequencies) or transition state structures (one negative imaginary frequency). Intrinsic reaction coordinate (IRC) calculations were performed in order to connect the TS to the reactant states and the product states. Single point energies were calculated using M06-2X-D3/6-31+G(d,p),[18] and SCRF=PCM (solvent= THF), for the BF_3 promoted addition of BuLi.
3D renderings of optimized structures were generated using GaussView 6.0.16[12] and CYLview[19] were used to generate the final structures.

Table S5. Energies of ring-opening with $BnNH_2$: B3LYP/6-31+G(d,p), SCRF=PCM (solvent= EtOH).

Structure	E (au)	H (au)	S (au)	G[a] (au)
**$BnNH_2$**	-326.945905	-326.7917310	84.886	-326.832063
**halfchair-6**	-385.089433	-384.926020	82.966	-384.965440
**TS-C(3)-$BnNH_2$**	-712.000504	-711.681931	128.330	-711.742905
**TS-C(4) -$BnNH_2$**	-711.993596	-711,674712	128.330	-711.735686
**Int-C(3)-$BnNH_2$**	-712.027990	-711.706191	124.514	-711,765351
**Int -C(4)-$BnNH_2$**	-712.013960	-711.691811	124.837	-711.751125

a) at 298.15 K.

Ergodic diagram of the ring-opening with $BnNH_2$ in EtOH

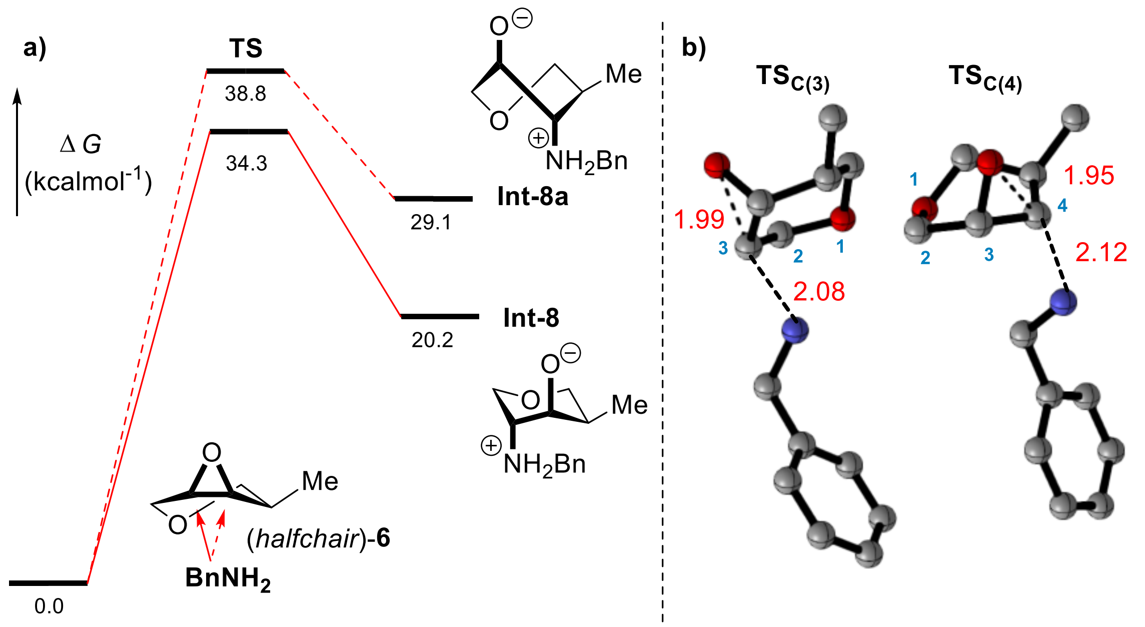

Figure S4. (a) DFT-computed energy profiles of the C(3) and C4) ring-opening of **6** in the half-chair conformation with $BnNH_2$: formation of both regioisomeric zwitterion intermediates **Int-8** and **Int-8a**; (b) the calculated structures of transition states **TS**$_{C(3)}$ and **TS**$_{C(4)}$. Hydrogen atoms are omitted for clarity; selected bond distances are labelled in Å.

BnNH₂

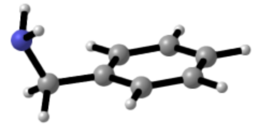

N	-2.79066000	0.00002000	0.65974100
H	-2.57116400	-0.81394200	1.23086700
C	-0.45274100	-0.00001500	-0.31538400
C	0.25309700	-1.20615600	-0.18162400
H	-0.27508200	-2.15056700	-0.29332300
C	0.25307400	1.20614200	-0.18164200
H	-0.27512200	2.15054200	-0.29335700
C	1.62510600	-1.20906100	0.08815700
H	2.15510700	-2.15254700	0.18520500
C	1.62508300	1.20907700	0.08813900
H	2.15506600	2.15257600	0.18517300
C	2.31534800	0.00001600	0.22556900
H	3.38180700	0.00002800	0.43172300
C	-1.95305000	-0.00003000	-0.55700800
H	-2.23016100	0.88059200	-1.14629100
H	-2.23015400	-0.88070300	-1.14621700
H	-2.57117500	0.81403800	1.23079200

halfchair-6

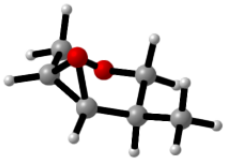

C	-1.86241200	0.31770300	0.09407300
C	-1.19033000	-0.99935500	-0.25015400
C	1.06402400	0.25308500	-0.45669800
C	0.28081500	1.33404800	0.30348300
H	-2.14610700	0.31493000	1.15815400
H	-2.76952200	0.43394400	-0.50456100
H	1.18094300	0.59387300	-1.49512900
H	0.25950800	1.10905900	1.38045700
H	0.75049900	2.31110600	0.16491300
H	-1.83242000	-1.76164400	-0.68935500
O	-1.05394500	1.45980300	-0.19478300
O	-0.26380000	-1.52397400	0.73416100
C	2.45459600	0.02600800	0.15185300
H	3.01886300	-0.71253500	-0.42719800
H	3.03268200	0.95629900	0.16230500
H	2.37767600	-0.33718000	1.18251200
C	0.25621100	-1.02877700	-0.52200100
H	0.65241400	-1.81076400	-1.17045900

TS-C(3)-BnNH₂

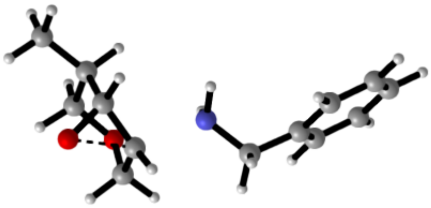

Imaginary frequency= -444.37

C	-1.93464600	1.39354600	-0.98134800
C	-1.72962400	-0.09296800	-1.15067100
C	-2.98192800	-0.47321800	1.02498400
C	-3.22377700	1.03996100	0.98285500
H	-2.80159300	1.71143500	-1.57433100
H	-1.06131500	1.94754000	-1.33303800
H	-2.11672000	-0.65389100	1.68075000
H	-4.14098500	1.26549100	0.41976200
H	-3.32556100	1.44755900	1.99242000
H	-1.33059600	-0.46489300	-2.08505900
O	-2.11715500	1.74211500	0.38951000
C	-4.19351700	-1.21618200	1.59780200
H	-3.99271200	-2.29130800	1.66681500
H	-4.44033900	-0.85717700	2.60339900
H	-5.07331400	-1.07791500	0.96079400
C	-2.60731100	-0.99446600	-0.37671200
H	-2.24410500	-2.03573000	-0.30077600
N	0.19349700	-0.16267800	-0.25914000
H	0.25383800	-1.08957500	0.15831400
C	2.69244600	-0.00485800	-0.47467700
C	3.37780500	-1.22674300	-0.39490100
H	2.95198500	-2.11295000	-0.85949100
C	3.26583400	1.13159500	0.11611900
H	2.75213200	2.08793300	0.05162500
C	4.60476900	-1.31432400	0.26925100
H	5.12486300	-2.26660500	0.31961900
C	4.49257100	1.04752800	0.78090000
H	4.92530900	1.93668500	1.23033700
C	5.16439500	-0.17688300	0.86052300
H	6.11928500	-0.24269600	1.37374800
C	1.34281000	0.07867600	-1.16113000
H	1.20808200	1.06862700	-1.60699500
H	1.28207300	-0.65839200	-1.96727700
O	-3.53681800	-0.77183900	-1.38680300
H	0.16802400	0.51243000	0.50374700

TS-C(4) -BnNH₂

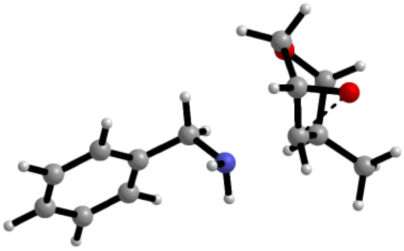

Imaginary frequency= -401.95

C	2.35809500	-1.71366200	0.80651000
C	2.39495200	-1.30909700	-0.67848100
C	2.32066300	1.16897900	0.02534500
C	2.97894600	0.55165100	1.27212400
H	1.48864100	-2.34368200	1.02364600
H	3.27032300	-2.29241500	1.01758800
H	1.39166800	1.64724300	0.35720200
H	2.94727700	1.26229300	2.10300800
H	4.02395500	0.29509000	1.05900400
H	1.88445900	-2.08722600	-1.27568200
O	2.26196100	-0.60402100	1.71135200
C	3.20172800	2.25870500	-0.60286300
H	3.41055000	3.05042100	0.12566400
H	2.70291600	2.71924200	-1.46239100
H	4.15102500	1.83492600	-0.94168100
O	3.64415300	-0.93406300	-1.15465900
C	-0.87253800	-0.13280600	0.35296700
H	-0.57759500	-1.12300800	0.70723100
H	-0.51682200	0.59690300	1.08426800
C	1.93755500	0.07366100	-0.95641500
H	1.87575800	0.33148700	-2.00641200
N	-0.14327200	0.11522300	-0.91511600
H	-0.39957500	-0.58238400	-1.61266100
C	-2.37937500	-0.04647000	0.21070600
C	-3.12533100	-1.17140400	-0.17422700
H	-2.62051500	-2.12007200	-0.34080200
C	-3.05128900	1.16422300	0.43805400
H	-2.48866300	2.04112300	0.74949200
C	-4.43698400	1.25252500	0.27481700
H	-4.94323700	2.19592000	0.45795800
C	-4.51059900	-1.08643200	-0.33874600
H	-5.07444000	-1.96671600	-0.63372000
C	-5.16980800	0.12721100	-0.11597600
H	-6.24673200	0.19360700	-0.23986500
H	-0.40109800	1.02286200	-1.30245900

Int-C(3)-BnNH$_2$

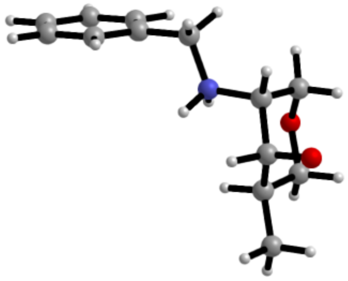

C	-1.80389200	1.51345500	-0.90026600
C	-1.37332300	0.06671200	-1.11092000
C	-2.68066300	-0.51386200	0.98743800
C	-2.98795600	0.98142000	1.09128000
H	-2.75756400	1.65588700	-1.42241800
H	-1.07944800	2.23787000	-1.28408300
H	-1.74895600	-0.69165500	1.55086400
H	-3.93897000	1.21431700	0.59246600

H	-3.04851200	1.30863100	2.13329200
H	-1.24422400	-0.16661400	-2.17005900
O	-1.94008200	1.77977300	0.49670300
C	-3.77846700	-1.35710800	1.63991700
H	-3.53070800	-2.42344900	1.58069400
H	-3.90192600	-1.09974300	2.69876700
H	-4.73604000	-1.20823600	1.13243600
C	-2.44447400	-0.91778800	-0.51242400
H	-1.93319500	-1.92194400	-0.47502300
N	-0.01042200	-0.12775500	-0.44924100
H	0.06320500	-1.10557500	-0.14654600
C	2.48386600	0.04164300	-0.53211300
C	3.14932300	-1.19357900	-0.51955800
H	2.74892000	-2.02962100	-1.08699500
C	3.02397400	1.11905700	0.18654000
H	2.52403100	2.08425400	0.17119900
C	4.33130800	-1.35192100	0.20831300
H	4.84043400	-2.31089400	0.20821400
C	4.20605700	0.96093700	0.91408400
H	4.61787600	1.80198000	1.46349800
C	4.86010600	-0.27526800	0.92724200
H	5.78110100	-0.39697300	1.48945000
C	1.19848800	0.20735400	-1.30321100
H	1.06267400	1.23407900	-1.64445000
H	1.15142400	-0.45962200	-2.16525700
O	-3.54828700	-0.89868400	-1.29026400
H	0.00369900	0.43657500	0.40920500

Int-C(4)-BnNH$_2$

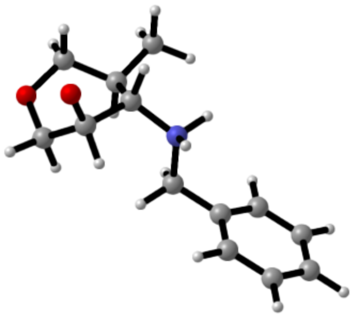

C	-2.69665700	-1.27468700	-1.06214500
C	-2.04970400	-1.39012800	0.33795900
C	-2.08592700	1.20487000	-0.07558000
C	-3.56326600	0.86429400	-0.32520800
H	-1.93338400	-1.04824400	-1.82662300
H	-3.15840700	-2.23216500	-1.31966100
H	-1.60497100	1.26202700	-1.05992300
H	-4.04999700	1.69388200	-0.84793800
H	-4.07157900	0.71896200	0.63922300
H	-1.14563700	-2.03683900	0.16047500
O	-3.74619400	-0.29379700	-1.14408600
C	-1.95215600	2.56865700	0.61870000
H	-2.38757500	3.35947200	-0.00061800
H	-0.90697400	2.84449500	0.80008900
H	-2.47061200	2.56854100	1.58414000
O	-2.87500000	-1.87409200	1.29866700
C	0.78653500	-0.04032800	-0.56023100
H	0.43884600	-0.95863000	-1.03321400
H	0.48711300	0.81090700	-1.16996700

C	-1.49656100	0.04110400	0.73656300
H	-1.76347000	0.16190600	1.78983600
N	0.03927300	0.07156100	0.75876900
H	0.33964100	-0.70267400	1.35980900
C	2.27887400	-0.05165300	-0.33737200
C	2.95559400	-1.25825800	-0.10220700
H	2.40579200	-2.19593000	-0.09556000
C	3.00592300	1.14800700	-0.36737300
H	2.49377300	2.08574600	-0.56713300
C	4.38661200	1.14333800	-0.15424200
H	4.94013900	2.07687800	-0.18406500
C	4.33612200	-1.26283700	0.11174400
H	4.85104400	-2.20204400	0.28912100
C	5.05289400	-0.06209300	0.08739300
H	6.12669000	-0.06674300	0.24865900
H	0.35450200	0.92091200	1.23730600

Table S6. Energies of the ring-opening with BuLi in presence of $BF_3{\cdot}Et_2O$: M06-2X-D3/6-31+G(d,p), SCRF=SMD (solvent= THF) single point energies calculated on the structures optimized with B3LYP augmented with Grimme's D3 empirical dispersion term in vacuum.

Structure	E (au)	H (au)	S (au)	G[a] (au)
**Bu_6Li_6**	-991.874072	-991.092378	262.215	-991.216964
**$BF_3{\cdot}Et_2O$**	-558.069276	-557.90783	95.802	-557.953349
**6**	-384.922915	-384.758369	82.193	-384.797422
**THF**	-232.358886	-232.235527	70.763	-232.269149
**Et_2O**	-233.556198	-233.412544	76.753	-233.449012
**RS-C(3)**	-1339.491712	-1338.928612	207.876	-1339.027381
**RS-C(4)**	-1339.490951	-1338.927672	210.686	-1339.027775
**TS-C(3)**	-1339.477400	-1338.916168	206.601	-1339.014331
**TS-C(4)**	-1339.476065	-1338.915560	203.718	-1339.012353
**Int-C(3)**	-1339.658548	-1339.090223	199.495	-1339.185009
**Int-C(4)**	-1339.646269	-1339.077992	202.638	-1339.174272

a) At 298.15 K.

Thermochemistry of the reactant state formation [RS-C(3) and RS-C(4)] at 195 K

1/6 Bu_6Li_6 + $BF_3{\cdot}Et_2O$ + (*halfchair*)-**6** + 2 THF ⟶ Et_2O + **RS-C(3)** ΔG_{195}= 1.62 kcal mol^{-1}

⇅

1/6 Bu_6Li_6 + $BF_3{\cdot}Et_2O$ + (*halfchair*)-**6** + 2THF ⟶ Et_2O + **RS-C(4)** ΔG_{195}= 1.66 kcal mol^{-1}

Bu_6Li_6

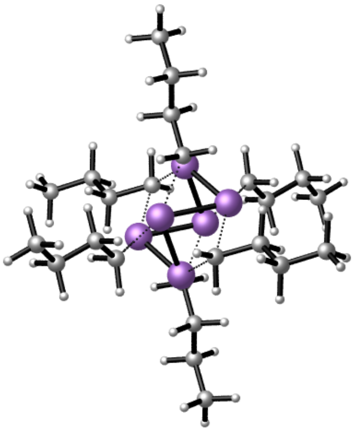

Li	-0.85274100	1.65020200	-0.18126000
Li	-0.85332400	-0.98195200	-1.33767500
Li	0.85633800	0.66770300	-1.51992700
Li	0.85663700	-1.65029600	0.18133300
Li	0.85679200	0.98190300	1.33807200
Li	-0.85281600	-0.66778500	1.51938300
C	-1.27506400	0.95211300	-2.15584900
H	-0.61409800	1.34778300	-2.95476000
H	-1.89173000	1.83913700	-1.90344500
C	-1.27434300	1.39121100	1.90290500
H	-1.89327200	0.72895200	2.54255100
H	-0.61351500	1.88239300	2.64703700
C	-1.27476500	-2.34353000	0.25385700
H	-1.89140900	-2.56896200	-0.64041300
H	-0.61329800	-3.23287500	0.31077200
C	1.27773200	-0.95253700	2.15625900
H	1.89600000	-1.83834400	1.90348600
H	0.61597400	-1.35035500	2.95347400
C	1.27731200	-1.39144100	-1.90321300
H	1.89759400	-0.72950600	-2.54189200
H	0.61601100	-1.88079300	-2.64814600
C	1.27845600	2.34322600	-0.25345300
H	0.61760400	3.23340000	-0.30417900
H	1.89936100	2.56475900	0.63886300
C	-2.24537200	-2.44578100	1.45522500
H	-1.71074000	-2.26954200	2.41156400
H	-3.00744400	-1.65004800	1.39162900
C	-2.97989800	-3.78687300	1.62347500
H	-2.23120700	-4.58632000	1.70639600
H	-3.54316800	-3.98980400	0.70281100
C	-3.92185700	-3.82306500	2.83261500
H	-4.42939500	-4.79002400	2.91952500
H	-3.37255500	-3.65031000	3.76635900
H	-4.69330100	-3.04714700	2.75498600
C	-2.24587600	-0.03722200	-2.84472900
H	-1.71103800	-0.95303100	-3.17138400
H	-3.00705000	-0.38087400	-2.12324800
C	-2.98199600	0.48803100	-4.08915400
H	-2.23428300	0.81667400	-4.82362300
H	-3.54530700	1.38642400	-3.80340800
C	-3.92429600	-0.54100500	-4.72457900
H	-4.43317100	-0.13238900	-5.60448600
H	-3.37502100	-1.43555900	-5.04321000

H	-4.69465600	-0.86257700	-4.01300900
C	-2.24222800	2.48543400	1.39140800
H	-1.70463800	3.22672500	0.76462300
H	-3.00306800	2.03552900	0.73049600
C	-2.97905000	3.29905600	2.46911600
H	-2.23176100	3.76766600	3.12370400
H	-3.54495700	2.60179100	3.10129500
C	-3.91814600	4.36715100	1.89651300
H	-4.42754000	4.92381400	2.69074400
H	-3.36622300	5.09086500	1.28407900
H	-4.68810800	3.91483400	1.25951100
C	2.24376800	2.44887300	-1.45876500
H	3.00499100	1.65172700	-1.40195500
H	1.70418600	2.27741000	-2.41311100
C	2.97990300	3.78935700	-1.62479300
H	3.54773100	3.98751500	-0.70589000
H	2.23222100	4.59041200	-1.70098200
C	3.91636900	3.82892700	-2.83808200
H	4.42516200	4.79537500	-2.92333300
H	4.68683700	3.05139400	-2.76719200
H	3.36249300	3.66095600	-3.76999300
C	2.24318000	-2.48777700	-1.39243300
H	1.70390900	-3.22894400	-0.76699600
H	3.00431100	-2.03989000	-0.73044600
C	2.97957000	-3.30113100	-2.47063900
H	2.23203800	-3.76779200	-3.12633700
H	3.54696900	-2.60392300	-3.10154400
C	3.91665000	-4.37135800	-1.89871700
H	3.36320200	-5.09500200	-1.28758100
H	4.42576000	-4.92779000	-2.69329200
H	4.68684900	-3.92100600	-1.26061000
C	2.24602800	0.03707500	2.84823900
H	3.00800700	0.38278900	2.12857400
H	1.70931200	0.95162500	3.17526100
C	2.98079600	-0.48901400	4.09311200
H	2.23229700	-0.81976300	4.82583300
H	3.54582000	-1.38621200	3.80699500
C	3.92060000	0.54037700	4.73164700
H	4.42853900	0.13116300	5.61181700
H	4.69172900	0.86405100	4.02186400
H	3.36955400	1.43370500	5.05065400

$BF_3 \cdot Et_2O$

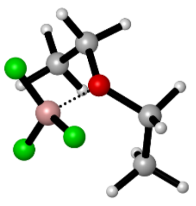

O	0.69273000	0.08270900	0.00000000
C	0.99210800	0.76277100	1.26191300
H	1.19657400	-0.04961900	1.95953400
H	1.91574700	1.32351400	1.09212000
C	0.99210800	0.76277100	-1.26191300
H	1.91574700	1.32351400	-1.09212000
H	1.19657400	-0.04961900	-1.95953400

C	-0.13934500	1.64719700	-1.76141700
H	-1.06576900	1.07530000	-1.84353500
H	-0.31338600	2.50398700	-1.10654400
H	0.12794000	2.02584100	-2.75408600
C	-0.13934500	1.64719700	1.76141700
H	-0.31338600	2.50398700	1.10654400
H	-1.06576900	1.07530000	1.84353500
H	0.12794000	2.02584100	2.75408600
B	-0.42357600	-1.15018000	0.00000000
F	-0.13934500	-1.82346400	1.15945600
F	-0.13934500	-1.82346400	-1.15945600
F	-1.65234700	-0.52956500	0.00000000

6

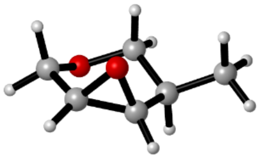

C	-1.86253700	0.30806200	0.09086300
C	-1.18150000	-1.00842900	-0.24542100
C	1.06069200	0.26260900	-0.46441300
C	0.26740600	1.32892200	0.30652700
H	-2.14698100	0.29793300	1.15584600
H	-2.76931600	0.42160300	-0.51046200
H	1.16995900	0.61283000	-1.50066400
H	0.24941700	1.08047600	1.37932300
H	0.73185600	2.31143600	0.18487000
H	-1.81917700	-1.78186200	-0.67603300
O	-1.06158500	1.45094000	-0.19539700
O	-0.24573300	-1.51049900	0.73404100
C	2.44686600	0.03703500	0.15182600
H	3.02451000	-0.68883200	-0.43074200
H	3.01921200	0.97085300	0.18624300
H	2.35450700	-0.34676100	1.17340600
C	0.26483300	-1.02674600	-0.52326900
H	0.67000100	-1.80991000	-1.16761500

THF

O	0.00057900	-1.20547500	-0.29565800
C	-1.13603000	-0.47060000	0.15884700
H	-2.00270300	-0.80800000	-0.41507400
H	-1.31553100	-0.68353700	1.22616600
C	1.13707100	-0.46924300	0.15711500
H	1.31987400	-0.68405800	1.22345100
H	2.00275300	-0.80405800	-0.41982000
C	0.77698000	1.01820200	-0.04919800
H	1.19610000	1.64912400	0.74045000
H	1.16550700	1.38046100	-1.00526800
C	-0.77869000	1.01664500	-0.05150800
H	-1.20172100	1.64986300	0.73420700
H	-1.16489400	1.37398500	-1.01037800

Et₂O

O	-0.60817900	-0.76154100	0.00000000
C	0.11224200	-0.58563900	1.21693400
H	-0.38637900	-1.23665700	1.94417900
H	1.14439700	-0.95360400	1.09930500
C	0.11224200	-0.58563900	-1.21693400
H	1.14439700	-0.95360400	-1.09930500
H	-0.38637900	-1.23665700	-1.94417900
C	0.11224200	0.85202800	-1.73865000
H	-0.91273700	1.23190200	-1.79388500
H	0.69286500	1.52220500	-1.09899000
H	0.54766500	0.88398600	-2.74472100
C	0.11224200	0.85202800	1.73865000
H	0.69286500	1.52220500	1.09899000
H	-0.91273700	1.23190200	1.79388500
H	0.54766500	0.88398600	2.74472100

RS-C(3)

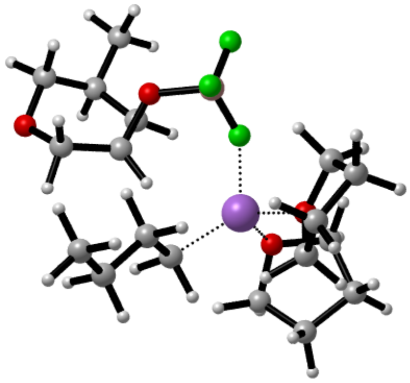

C	3.13519500	1.48753700	-0.75868900
C	2.20460300	0.32176300	-0.52443500
C	4.25012200	-1.26172600	-0.52916600
C	4.99375500	0.07056600	-0.32956700
H	3.16463300	2.10793400	0.14958300
H	2.73177800	2.09132000	-1.57497000
H	4.42195400	-1.58767700	-1.56457400
H	4.95683800	0.36764700	0.73000700
H	6.04119300	-0.03520800	-0.62258500
H	1.13855400	0.44867300	-0.78283900
O	4.44596300	1.09368400	-1.15621200
O	2.38955100	-0.31867700	0.83187500
C	4.72539100	-2.34521900	0.44627100
H	4.22722100	-3.29994900	0.24936900
H	5.80510800	-2.50181700	0.35367400
H	4.50304100	-2.05873600	1.47888300
C	2.76290900	-1.03326600	-0.41923200
H	2.09993500	-1.87336200	-0.61650500
B	1.16527700	-0.80021000	1.71461300
F	1.67393300	-1.79894700	2.50079100
F	0.18826300	-1.29038200	0.81796800
F	0.73128500	0.31734400	2.37689500
C	-0.65277000	1.35651100	-1.47119500
H	0.04049400	1.38910000	-2.33752600
H	-1.65021400	1.45635400	-1.95128800
O	-2.66300700	-0.22715500	0.79777800
O	-1.52608500	-1.97142200	-1.51894600

C	-3.57931400	0.86074500	0.52385600
H	-3.15072200	1.46325000	-0.27879600
H	-4.53771900	0.43805500	0.18842100
C	-2.59258700	-0.46535400	2.23212800
H	-2.76464300	-1.53200500	2.40926400
H	-1.58655300	-0.20365500	2.57013400
C	-2.01780100	-1.60311200	-2.82185100
H	-1.48146700	-0.70363700	-3.12729700
H	-1.80869600	-2.42147900	-3.52761900
C	-2.48975000	-2.89451400	-0.97979100
H	-2.37350000	-2.89006600	0.10549000
H	-2.26747500	-3.90142800	-1.36088900
Li	-1.07924100	-0.39922900	-0.38964400
C	-0.42039500	2.61927100	-0.62355500
H	-1.17648000	2.67637400	0.17884000
H	0.53777100	2.54851800	-0.07487000
C	-0.42067000	3.97213900	-1.36266000
H	0.34428300	3.94022000	-2.15264200
H	-1.38228100	4.08804900	-1.88280900
C	-0.17281900	5.17929300	-0.44795400
H	0.79556900	5.09388900	0.06182400
H	-0.17493700	6.12537600	-1.00258700
H	-0.94433700	5.24702300	0.32983200
C	-3.73106700	1.59864800	1.85100100
H	-4.66231800	2.16912000	1.91071600
H	-2.89088600	2.28692900	1.99343100
C	-3.65740600	0.44029100	2.85918200
H	-3.38085900	0.75970400	3.86759400
H	-4.62334300	-0.07518400	2.91669400
C	-3.86917600	-2.39536600	-1.46665200
H	-4.40130900	-1.88660700	-0.65948300
H	-4.49019000	-3.22789100	-1.81011700
C	-3.52584800	-1.40630400	-2.61597200
H	-4.09566700	-1.60024200	-3.52911500
H	-3.72038900	-0.37486500	-2.30854000

RS-C(4)

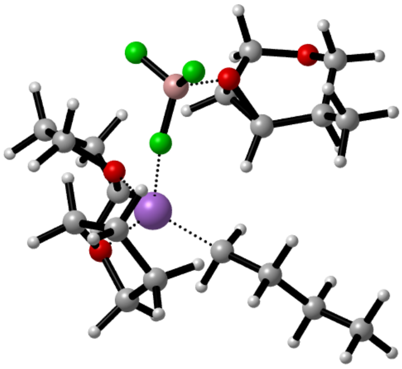

C	2.97585900	-2.25468300	0.32224900
C	1.73853800	-1.41369200	0.09405700
C	3.23735000	0.63719800	-0.33000100
C	4.30689400	-0.45660000	-0.48247400
H	3.09115100	-2.95397000	-0.52078100
H	2.84919100	-2.83542600	1.24012600
H	3.39948000	1.14288000	0.63175300
H	4.26254200	-0.89327400	-1.49217200
H	5.30413900	-0.03768000	-0.32960900

H	0.79278200	-1.75611300	0.50923300
O	4.14903600	-1.47610700	0.50669700
O	1.58543000	-0.96487200	-1.32023300
C	3.28741900	1.66254400	-1.46896800
H	2.55870800	2.46157200	-1.30392700
H	4.28107400	2.11769500	-1.53514900
H	3.06099600	1.18553200	-2.42819500
C	1.87229700	0.01608300	-0.20864100
H	1.01110700	0.64475900	0.03016000
B	0.18463200	-1.07083300	-2.06179800
F	0.39070800	-0.50805700	-3.28844300
F	-0.74299800	-0.34362600	-1.29499000
F	-0.10923000	-2.41686500	-2.06585300
C	-0.26860500	1.47015300	1.73661600
H	0.57279800	0.94146100	2.23721600
H	-0.99964300	1.61632200	2.56069800
O	-1.41816300	-1.63890500	1.21048000
O	-3.19421600	0.76658600	-0.09343700
C	-1.27103600	-1.84568200	2.62956800
H	-0.42586200	-1.23731000	2.95746700
H	-2.17621000	-1.50304300	3.15198200
C	-2.15035300	-2.75863400	0.64790500
H	-1.65961200	-3.01276400	-0.29421400
H	-3.17879500	-2.43980400	0.44071200
C	-3.33975000	2.19610300	0.04993500
H	-4.40302500	2.42909200	0.21107700
H	-2.75919600	2.49569200	0.92357900
C	-3.63576100	0.46818900	-1.43338400
H	-4.73318300	0.40143800	-1.43284400
H	-3.21613800	-0.50001100	-1.71088100
Li	-1.40173400	0.19866800	0.52131600
C	0.23580700	2.86381000	1.32482700
H	0.93901800	2.78904200	0.47294300
H	-0.60241500	3.46370700	0.92949900
C	0.93703300	3.70651800	2.40930300
H	0.24531900	3.83641200	3.25360600
H	1.78712500	3.13025500	2.80361900
C	1.42235700	5.07630500	1.91549200
H	0.58381600	5.67942700	1.54438300
H	1.91945500	5.64916500	2.70762600
H	2.13513200	4.96581300	1.08755800
C	-2.09153500	-3.88624300	1.70497700
H	-3.07469500	-4.03380200	2.16317600
H	-1.78124800	-4.84040900	1.27089200
C	-1.08955900	-3.35590300	2.75255100
H	-1.28718000	-3.72927400	3.76119200
H	-0.06300800	-3.62941300	2.48204100
C	-2.82618200	2.76557900	-1.27910100
H	-3.30644700	3.71573300	-1.52970700
H	-1.74853000	2.93727600	-1.21107100
C	-3.13774000	1.63992600	-2.30685700
H	-2.23833300	1.35510000	-2.85724800
H	-3.89949200	1.93957400	-3.03252700

TS-C(3)

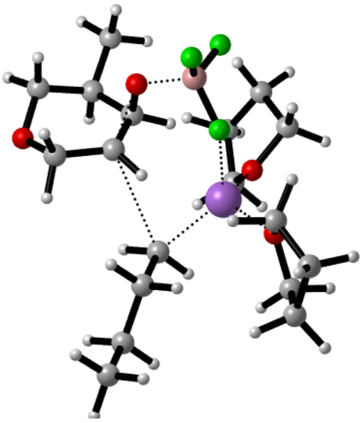

Imaginary frequency= -346.33

C	1.20602400	3.01856000	0.65909800
C	0.94621200	1.55534500	0.50881400
C	3.28819900	1.26960600	-0.48522800
C	3.49260800	2.66007400	0.13300100
H	1.28851800	3.26198900	1.73088200
H	0.33544500	3.54698300	0.25661100
H	3.14148400	1.40533500	-1.56551300
H	3.72354800	2.56787400	1.20543700
H	4.31932800	3.17944300	-0.35759900
H	-0.05407800	1.17407400	0.63134600
O	2.33894600	3.47943400	-0.06157600
O	1.98540200	0.56686300	1.49983100
C	4.48202500	0.34410900	-0.22733400
H	4.34832200	-0.62208200	-0.72301100
H	5.40755500	0.79163700	-0.60425200
H	4.59770800	0.15415700	0.84380300
C	2.00024400	0.66351600	0.03740600
H	1.69078100	-0.25831400	-0.44915600
B	1.36329700	-0.73317500	2.05560400
F	2.23220900	-1.76226500	1.71922800
F	0.11057500	-0.94389600	1.38540100
F	1.17767600	-0.55189400	3.39880900
C	-1.22076200	1.13280800	-1.38245100
H	-0.56553700	1.80441700	-1.96259100
H	-1.68792000	0.47720900	-2.14805700
O	-2.55848300	-1.43632600	0.28441300
O	0.20288400	-1.96562800	-1.41199700
C	-3.74536700	-1.44356700	-0.54953700
H	-3.58972200	-0.74459300	-1.37623300
H	-3.86889200	-2.45679200	-0.95236600
C	-2.92430100	-1.24592500	1.67567500
H	-2.29535800	-1.90360500	2.27887900
H	-2.71634900	-0.20515700	1.95519300
C	0.71630700	-1.69209000	-2.72842100
H	0.16709600	-2.30319800	-3.45859900
H	0.52757500	-0.63608500	-2.93197500
C	0.85461100	-3.16686100	-0.95540500
H	0.35712300	-4.03599900	-1.40972300
H	0.74968600	-3.20434100	0.12926200
Li	-0.87489400	-0.73382000	-0.38345200
C	-2.31818000	1.96085500	-0.70506600
H	-2.97891900	1.30527600	-0.11198700
H	-1.87311600	2.64596800	0.04095900

C	-3.21918800	2.81304600	-1.62244900
H	-2.58474600	3.50187200	-2.19737300
H	-3.69060700	2.14965300	-2.36179800
C	-4.29865100	3.60003500	-0.86746200
H	-3.84841100	4.28923100	-0.14175600
H	-4.92570700	4.19236500	-1.54416700
H	-4.95907200	2.92396400	-0.30860700
C	-4.90454700	-1.05029900	0.37072900
H	-5.85340100	-1.49327000	0.05556300
H	-5.02278300	0.03850800	0.38724800
C	-4.41868400	-1.55533000	1.73962000
H	-4.90992500	-1.06060800	2.58206600
H	-4.58235400	-2.63525500	1.83187000
C	2.30787400	-3.02264600	-1.43215300
H	2.75297100	-3.99181400	-1.67328800
H	2.90536800	-2.56692600	-0.63902800
C	2.20714200	-2.08662400	-2.67128200
H	2.83948200	-1.20260400	-2.54656200
H	2.51957500	-2.58190700	-3.59477400

TS-C(4)

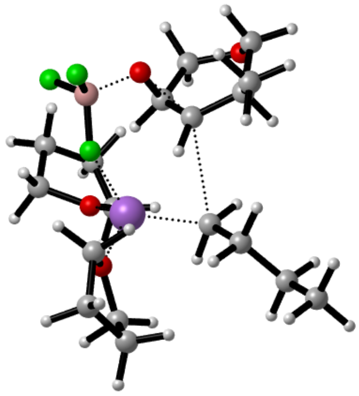

Imaginary frequency= -339.87

C	-3.22416700	1.25666600	-0.88181400
C	-2.03838600	0.62075800	-0.17098700
C	-1.09970000	2.95797600	0.29373100
C	-2.56322900	3.34893100	0.03445400
H	-4.12981200	1.04092900	-0.29458900
H	-3.33386500	0.81253300	-1.87455100
H	-0.50648800	3.27767000	-0.57810200
H	-3.17119500	3.14121800	0.92813000
H	-2.63655300	4.41372200	-0.19884800
H	-1.78310100	-0.37529100	-0.52152100
O	-3.07767800	2.65429800	-1.09854000
O	-2.13702200	0.69279100	1.29193700
C	-0.52858000	3.59449400	1.56642800
H	0.52771400	3.33622800	1.68823900
H	-0.60775600	4.68555900	1.52034200
H	-1.07169600	3.24140800	2.44859800
C	-0.95050200	1.47516500	0.28590700
H	-0.00405400	1.03147100	0.53673200
B	-1.64394000	-0.57153100	2.03252400
F	-1.46561900	-0.22566200	3.34497600
F	-0.40177300	-0.97650000	1.43347900
F	-2.59259400	-1.55841600	1.80943900
C	1.10484300	0.73710800	-1.58751000

H	0.45045500	1.33091300	-2.24895500
H	1.55936400	-0.02184100	-2.26031700
O	-0.46133600	-2.26970500	-1.23890800
O	2.30192700	-1.60457400	0.47032700
C	-0.93699400	-2.14121500	-2.59267700
H	-0.70105400	-1.12604000	-2.91838500
H	-0.39585300	-2.85608200	-3.22786000
C	-1.19592300	-3.35734300	-0.64219400
H	-1.09989500	-3.26292200	0.43971100
H	-0.75686900	-4.30883100	-0.97599900
C	3.51711800	-1.75084700	-0.30926400
H	3.62798100	-2.81239400	-0.56352500
H	3.40832800	-1.16983100	-1.22945300
C	2.62328800	-1.21629200	1.83064600
H	1.95296100	-1.75951500	2.49931500
H	2.43846200	-0.13995100	1.94348700
Li	0.66871300	-0.96860600	-0.36063000
C	2.22031000	1.63715500	-1.04272900
H	1.78597400	2.44967200	-0.43239300
H	2.85559800	1.07377600	-0.33667500
C	3.15660400	2.29575100	-2.07626600
H	3.61820200	1.50433300	-2.68427500
H	2.54855500	2.89219100	-2.77068100
C	4.24864400	3.17240300	-1.44883200
H	4.88302500	2.58550800	-0.77160300
H	4.90038100	3.62413500	-2.20590500
H	3.80881500	3.98692700	-0.85919900
C	-2.62969300	-3.17417400	-1.15623800
H	-3.15808600	-4.12786100	-1.23862800
H	-3.18502200	-2.54202800	-0.45902000
C	-2.44536300	-2.47066400	-2.53136400
H	-2.73922400	-3.11161900	-3.36723500
H	-3.05154000	-1.56203500	-2.59180800
C	4.65358600	-1.25779500	0.59147300
H	5.59943200	-1.76453200	0.38068600
H	4.80389500	-0.18117200	0.45723200
C	4.10440700	-1.54858200	1.99823300
H	4.57941000	-0.94946500	2.78004900
H	4.23223900	-2.60752900	2.25071900

Int-7

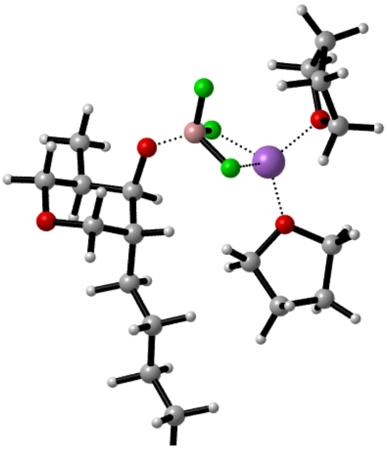

C	-2.70564700	-1.86245900	-1.50139600
C	-2.06546900	-0.82023700	-0.57208400
C	-2.12365300	-2.61496700	1.22764300

C	-2.77489100	-3.52553500	0.17912700
H	-1.91513500	-2.41154400	-2.03384100
H	-3.35994700	-1.38906400	-2.23989500
H	-2.93223800	-2.07549200	1.73999400
H	-1.99723800	-4.11362300	-0.33326100
H	-3.48159200	-4.21672700	0.64879800
H	-1.34057500	-0.24918700	-1.16595700
O	-3.53039300	-2.77697800	-0.77699200
O	-0.13467300	-2.21176700	-0.07148300
C	-1.32531800	-3.41793100	2.25743100
H	-0.87575500	-2.75400300	3.00441900
H	-1.96735500	-4.13554600	2.78140300
H	-0.51254600	-3.96408200	1.77024800
C	-1.25479500	-1.55993600	0.51788800
H	-0.90789800	-0.83476700	1.27007700
B	1.11037100	-1.57680600	-0.07039600
F	1.54447000	-1.14818500	1.28000000
F	1.09736800	-0.25742100	-0.75283200
F	2.11856400	-2.34278700	-0.63101800
C	-3.10627200	0.14731100	0.02460800
H	-4.01345200	-0.40975400	0.28750400
H	-2.72305300	0.56422600	0.96753900
O	1.15489500	2.18678800	1.10114200
O	3.86291000	0.64375300	0.14774500
C	1.70097600	3.53066600	1.06417700
H	2.34363400	3.66253300	1.93916000
H	2.31096600	3.63073900	0.15752900
C	-0.28127500	2.21902600	0.86654800
H	-0.51975100	1.40810300	0.17922500
H	-0.79086900	2.04727100	1.82234200
C	4.68767200	-0.51683800	0.49051000
H	5.37244200	-0.21696000	1.28959200
H	4.01632500	-1.30040900	0.85112700
C	4.00274200	0.93663500	-1.26786400
H	4.74021200	1.74217100	-1.38860000
H	3.02953400	1.27435300	-1.63485800
Li	2.03364900	0.55002000	0.69057700
C	-3.46815200	1.30816700	-0.91077500
H	-2.54984800	1.83732700	-1.20656200
H	-3.90100800	0.91555000	-1.84110100
C	-4.44299400	2.31276300	-0.28581100
H	-5.36921100	1.79301400	-0.00617300
H	-4.01388700	2.69283700	0.65305900
C	-4.76994400	3.48895900	-1.21265300
H	-5.22883200	3.13892600	-2.14480800
H	-5.46454600	4.19350300	-0.74226700
H	-3.86154400	4.04254700	-1.48135900
C	0.48785800	4.46476000	1.03906800
H	0.70424300	5.41075100	0.53544600
H	0.15677400	4.68871400	2.05934900
C	-0.56526800	3.61206200	0.31168100
H	-1.59285000	3.93224800	0.50233800
H	-0.39710200	3.63090400	-0.77099000
C	4.47172100	-0.37126400	-1.89614600
H	4.97786500	-0.21692600	-2.85344900
H	3.61742000	-1.03787200	-2.03986400
C	5.40117700	-0.92679300	-0.80366700
H	5.52640900	-2.01050500	-0.86787500
H	6.39126200	-0.46044000	-0.86639400

Int-7a

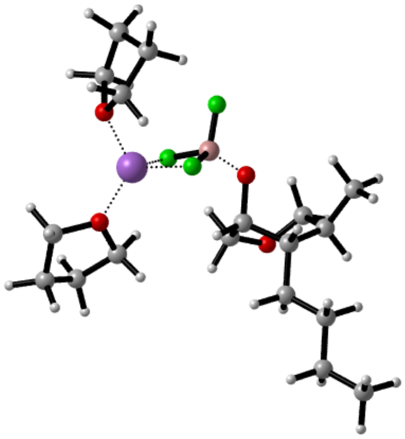

C	-1.47741800	0.20793000	-1.89777900
C	-1.29575000	-0.34672300	-0.46827900
C	-3.35299000	-1.80932000	-0.76742200
C	-2.83462900	-1.70903100	-2.21567900
H	-0.55396700	0.02012200	-2.46268800
H	-1.65923800	1.28866900	-1.86247200
H	-4.42409200	-1.57120000	-0.79288700
H	-1.91145900	-2.29377400	-2.33244500
H	-3.57873200	-2.08982100	-2.92160500
H	-0.84546900	0.43813900	0.15575300
O	-2.60073700	-0.34626600	-2.58378900
O	-0.45022000	-1.49505300	-0.46905700
C	-3.18873900	-3.23838000	-0.23597400
H	-3.64794800	-3.34154100	0.75397000
H	-3.66503500	-3.96817200	-0.90301200
H	-2.12688700	-3.48747500	-0.14949900
C	-2.65201300	-0.74479000	0.13464200
H	-2.43011400	-1.19891600	1.10902500
B	0.91074000	-1.34309800	-0.18939800
F	1.60911700	-2.53820300	-0.18918700
F	1.16307500	-0.64554700	1.09021600
F	1.59679300	-0.40767100	-1.12061100
C	-3.53295800	0.49789900	0.37497700
H	-3.91171100	0.86384200	-0.58802200
H	-2.91498800	1.30474900	0.79724500
O	2.10030700	2.35949300	0.07026100
O	4.13701900	-0.21933600	0.72135100
C	0.97826100	2.76620100	-0.76365000
H	0.81998800	1.98503400	-1.50913100
H	0.08698600	2.84248600	-0.12839700
C	2.97331400	3.48919300	0.32789000
H	3.91404800	3.32700000	-0.21259600
H	3.18184400	3.52347600	1.40124400
C	4.20094100	-0.91669500	1.99712400
H	5.05324100	-0.52076500	2.56539100
H	3.27238500	-0.71110000	2.53582600
C	4.71464600	-1.06047900	-0.32746600
H	5.47866500	-0.47236100	-0.84505900
H	3.91176900	-1.32014800	-1.02349700
Li	2.41964200	0.50329200	0.29623100
C	-4.70656300	0.24518800	1.32953700
H	-5.32395600	-0.58218400	0.95345000

H	-4.31461300	-0.08644700	2.30227000
C	-5.59749000	1.47649300	1.53445000
H	-4.98420500	2.30853100	1.90883000
H	-5.99137000	1.80126800	0.56137000
C	-6.76038300	1.22213500	2.50026900
H	-6.39224100	0.92493900	3.48969100
H	-7.38123000	2.11572400	2.62881800
H	-7.40674900	0.41590800	2.13303900
C	2.22012400	4.71594900	-0.19325800
H	1.56436800	5.12161800	0.58531300
H	2.89725100	5.51080200	-0.51730500
C	1.38380800	4.12102700	-1.33861000
H	0.51913400	4.73414400	-1.60615000
H	1.99953900	3.98597000	-2.23493800
C	4.38279900	-2.38780600	1.63727000
H	4.84059400	-2.96093000	2.44862500
H	3.41479100	-2.82822200	1.38368900
C	5.27098700	-2.29697000	0.38517200
H	5.20955900	-3.18806900	-0.24480400
H	6.31971200	-2.14453300	0.66608000

Single Crystal X-ray diffraction

A crystal of compound **5** ($C_6H_{11}ClO_2$) was mounted in air at ambient conditions. All measurements were made on a *Rigaku Oxford Diffraction Synergy* area-detector diffractomete[20] using mirror optics monochromated Cu *K*α radiation (λ = 1.54184 Å). The unit cell constants and an orientation matrix for data collection were obtained from a least-squares refinement of the setting angles of reflections in the range 2 < θ < 76°. A total of 2904 frames were collected using ω scans, with x seconds exposure time, a rotation angle of 0.5° per frame, a crystal-detector distance of 35.0 mm, at T = 173(2) K.

Data reduction was performed using the *CrysAlisPro*[19] program. The intensities were corrected for Lorentz and polarization effects, and an absorption correction based on the multi-scan method using SCALE3 ABSPACK in *CrysAlisPro*[19] was applied. Data collection and refinement parameters are given in *Table 1.*

The structure was solved by direct methods using SHELXT,[21] which revealed the positions of all non-hydrogen atoms of the title compound. The non-hydrogen atoms were refined anisotropically. All H-atoms were placed in geometrically calculated positions and refined using a riding model where each H-atom was assigned a fixed isotropic displacement parameter with a value equal to 1.2Ueq of its parent atom.

Refinement of the structure was carried out on F^2 using full-matrix least-squares procedures, which minimized the function $\Sigma w(F_o^2 - F_c^2)^2$. The weighting scheme was based on counting statistics and included a factor to downweight the intense reflections. The absolute structure configuration was determined with the Flack parameter[22] (0.00(2)) and confirmed with the Parsons's selected quotients[23] (0.00(1)). Data have been deposited at the Cambridge Crystallographic Data Center, with Deposition Number CCDC-2122799.

All calculations were performed using the SHELXL-2014/7 program.[24]

Table S7. Crystal data and structure refinement for **5**.

Empirical formula	C6 H11 Cl O2	
Formula weight	150.60	
Temperature	173(2) K	
Wavelength	1.54184 Å	
Crystal system	Orthorhombic	
Space group	P 21 21 21	
Unit cell dimensions	a = 7.83960(10) Å	α= 90°.
	b = 7.95970(10) Å	β= 90°.
	c = 11.8452(2) Å	γ = 90°.
Volume	739.151(18) Å^3	
Z	4	
Density (calculated)	1.353 Mg/m^3	
Absorption coefficient	4.002 mm^{-1}	
F(000)	320	
Crystal size	0.13 x 0.33 x 0.36 mm^3	
Theta range for data collection	6.701 to 76.774°.	
Index ranges	-9<=h<=9, -9<=k<=10, -14<=l<=14	
Reflections collected	6544	
Independent reflections	1498 [R(int) = 0.0308]	
Completeness to theta = 67.684°	99.6 %	
Refinement method	Full-matrix least-squares on F^2	
Data / restraints / parameters	1498 / 0 / 84	
Goodness-of-fit on F^2	1.087	
Final R indices [I>2sigma(I)]	R1 = 0.0258, wR2 = 0.0718	
R indices (all data)	R1 = 0.0259, wR2 = 0.0719	
Absolute structure parameter	0.001(8)	
Largest diff. peak and hole	0.167 and -0.276 e.Å^{-3}	

Table S8. Atomic coordinates (x 10^4) and equivalent isotropic displacement parameters ($Å^2$x 10^3) for compound **5**. U_{eq} is defined as one third of the trace of the orthogonalized U^{ij} tensor.

	x	y	z	U(eq)
C(1)	5082(2)	4727(3)	5130(2)	26(1)
C(2)	6980(2)	4902(3)	5359(2)	28(1)
C(3)	7374(3)	4018(3)	6471(2)	36(1)
C(4)	4603(3)	4514(3)	7172(2)	34(1)
C(5)	4031(2)	5429(3)	6104(2)	28(1)
C(6)	2109(3)	5188(3)	5945(2)	37(1)
O(1)	8004(2)	4084(2)	4539(1)	35(1)
O(2)	6392(2)	4706(2)	7370(1)	40(1)
Cl(1)	4574(1)	5804(1)	3828(1)	39(1)

Table S9. Bond lengths [Å] and angles [°] for compound **5**.

C(1)-C(2)	1.519(3)
C(1)-C(5)	1.524(3)
C(1)-Cl(1)	1.810(2)
C(1)-H(1)	1.0000
C(2)-O(1)	1.418(2)
C(2)-C(3)	1.525(3)
C(2)-H(2)	1.0000
C(3)-O(2)	1.423(3)
C(3)-H(3A)	0.9900
C(3)-H(3B)	0.9900
C(4)-O(2)	1.430(3)
C(4)-C(5)	1.527(3)
C(4)-H(4A)	0.9900
C(4)-H(4B)	0.9900
C(5)-C(6)	1.531(3)
C(5)-H(5)	1.0000
C(6)-H(6A)	0.9800
C(6)-H(6B)	0.9800
C(6)-H(6C)	0.9800
O(1)-H(1A)	0.8400
C(2)-C(1)-C(5)	111.16(16)
C(2)-C(1)-Cl(1)	108.90(14)
C(5)-C(1)-Cl(1)	110.67(14)
C(2)-C(1)-H(1)	108.7
C(5)-C(1)-H(1)	108.7
Cl(1)-C(1)-H(1)	108.7
O(1)-C(2)-C(1)	112.95(17)
O(1)-C(2)-C(3)	105.38(16)
C(1)-C(2)-C(3)	108.06(16)
O(1)-C(2)-H(2)	110.1
C(1)-C(2)-H(2)	110.1
C(3)-C(2)-H(2)	110.1
O(2)-C(3)-C(2)	111.04(18)
O(2)-C(3)-H(3A)	109.4
C(2)-C(3)-H(3A)	109.4
O(2)-C(3)-H(3B)	109.4
C(2)-C(3)-H(3B)	109.4
H(3A)-C(3)-H(3B)	108.0
O(2)-C(4)-C(5)	111.91(18)
O(2)-C(4)-H(4A)	109.2

C(5)-C(4)-H(4A)	109.2
O(2)-C(4)-H(4B)	109.2
C(5)-C(4)-H(4B)	109.2
H(4A)-C(4)-H(4B)	107.9
C(1)-C(5)-C(4)	107.05(16)
C(1)-C(5)-C(6)	113.12(18)
C(4)-C(5)-C(6)	109.37(17)
C(1)-C(5)-H(5)	109.1
C(4)-C(5)-H(5)	109.1
C(6)-C(5)-H(5)	109.1
C(5)-C(6)-H(6A)	109.5
C(5)-C(6)-H(6B)	109.5
H(6A)-C(6)-H(6B)	109.5
C(5)-C(6)-H(6C)	109.5
H(6A)-C(6)-H(6C)	109.5
H(6B)-C(6)-H(6C)	109.5
C(2)-O(1)-H(1A)	109.5
C(3)-O(2)-C(4)	111.49(16)

Symmetry transformations used to generate equivalent atoms:

Table S10. Anisotropic displacement parameters ($Å^2$x 10^3) for compound **5**. The anisotropic displacement factor exponent takes the form: $-2\pi^2[h^2a^{*2}U^{11} + ... + 2 h k a^* b^* U^{12}]$

	U^{11}	U^{22}	U^{33}	U^{23}	U^{13}	U^{12}
C(1)	25(1)	30(1)	21(1)	0(1)	-2(1)	-2(1)
C(2)	22(1)	37(1)	25(1)	-2(1)	1(1)	-1(1)
C(3)	25(1)	57(1)	25(1)	1(1)	0(1)	4(1)
C(4)	26(1)	50(1)	24(1)	-1(1)	3(1)	2(1)
C(5)	24(1)	32(1)	28(1)	-2(1)	2(1)	0(1)
C(6)	24(1)	49(1)	37(1)	1(1)	3(1)	4(1)
O(1)	29(1)	49(1)	27(1)	4(1)	7(1)	9(1)
O(2)	28(1)	67(1)	24(1)	-7(1)	-3(1)	0(1)
Cl(1)	32(1)	59(1)	26(1)	8(1)	-2(1)	4(1)

Figure S5. ORTEP Plot [25] of compound **5** as determined from the X-ray diffraction experiment. Ellipsoids are drawn at 90% probability level.

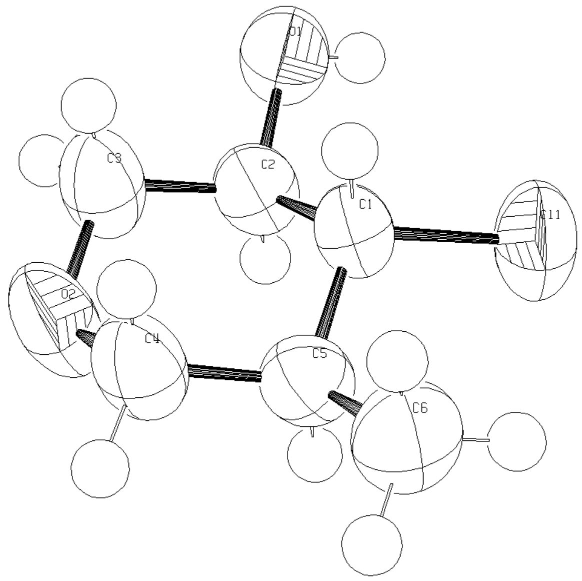

Copies of ^{1}H and $^{13}C\{^{1}H\}$ NMR spectra

1,1'-Oxybis(propan-2-one) (10)

^1H NMR ($CDCl_3$, 400 MHz)

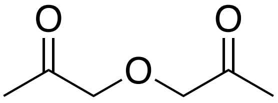

10

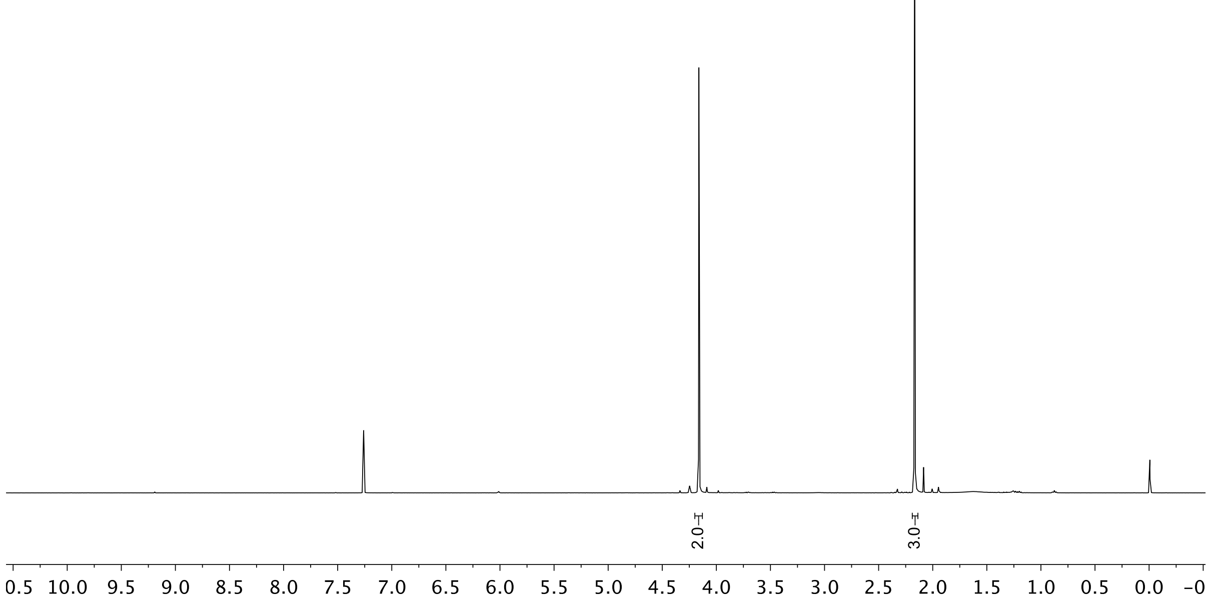

$^{13}C\{^1H\}$ NMR ($CDCl_3$, 101 MHz)

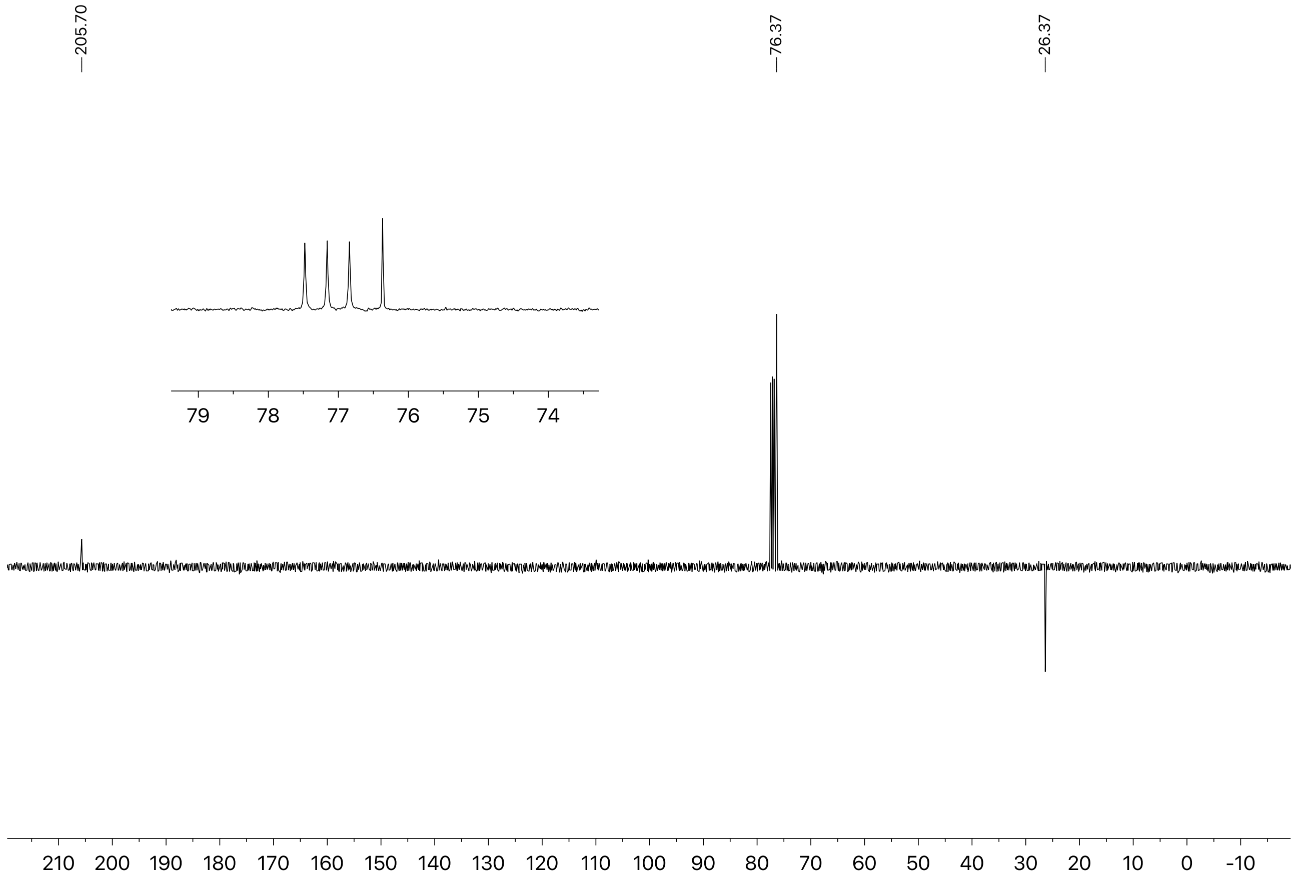

5-Methyl-2*H*-pyran-3(6*H*)-one (2)

^{1}H NMR ($CDCl_3$, 400 MHz)

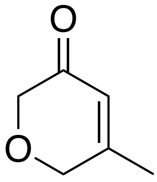

2

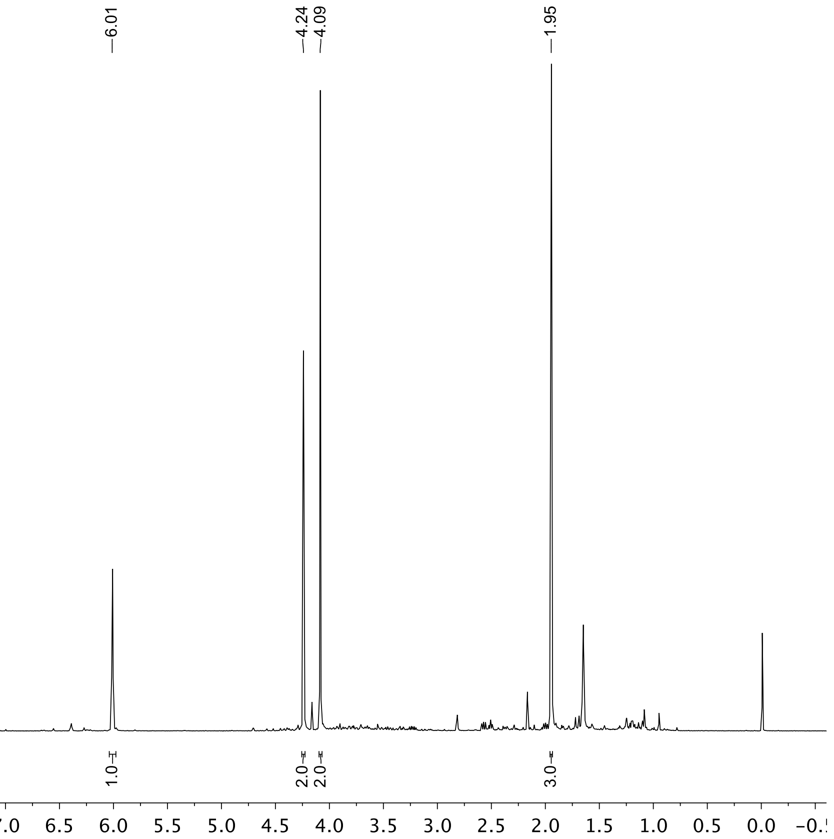

^{13}C{^{1}H} NMR ($CDCl_3$, 101 MHz)

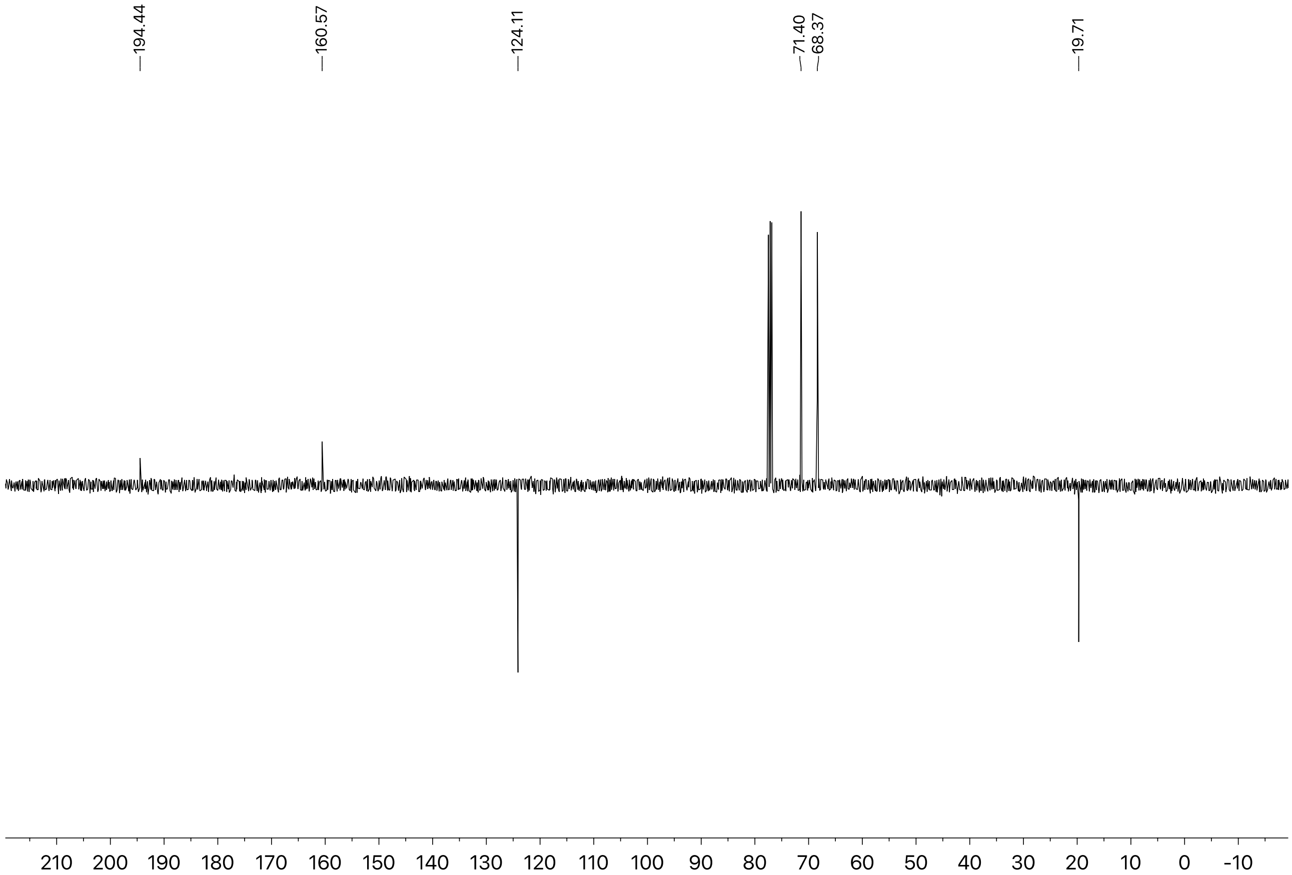

1-Methyl-3,7-dioxabicyclo[4.1.0]heptan-5-one (3)

^{1}H NMR ($CDCl_3$, 400 MHz)

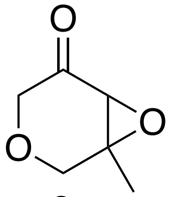

3

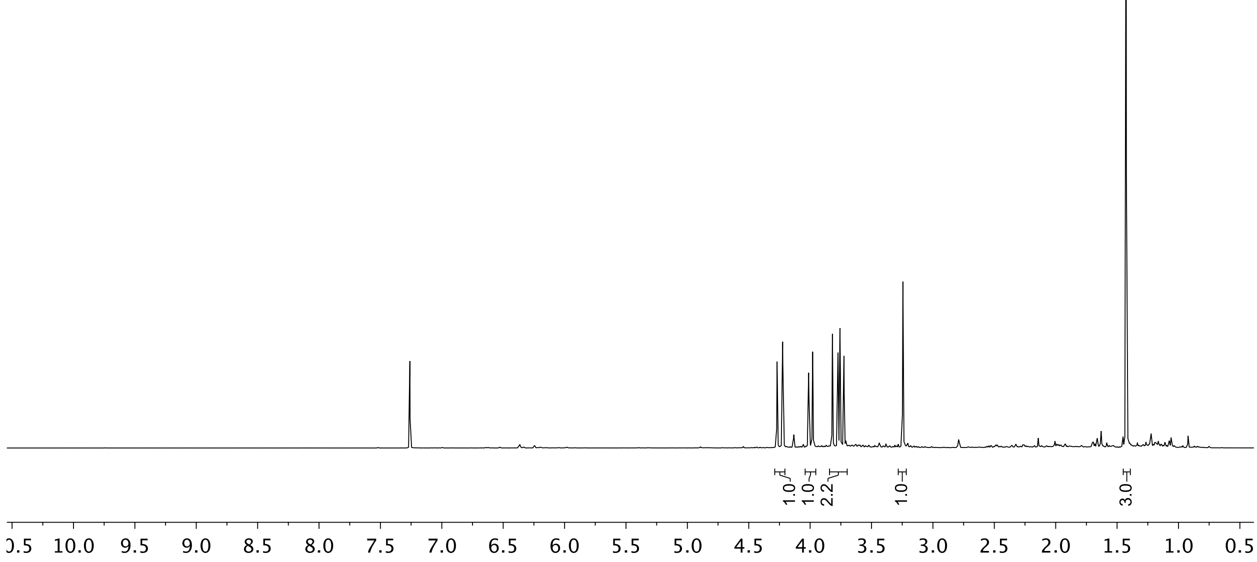

$^{13}C\{^1H\}$ NMR ($CDCl_3$, 101 MHz)

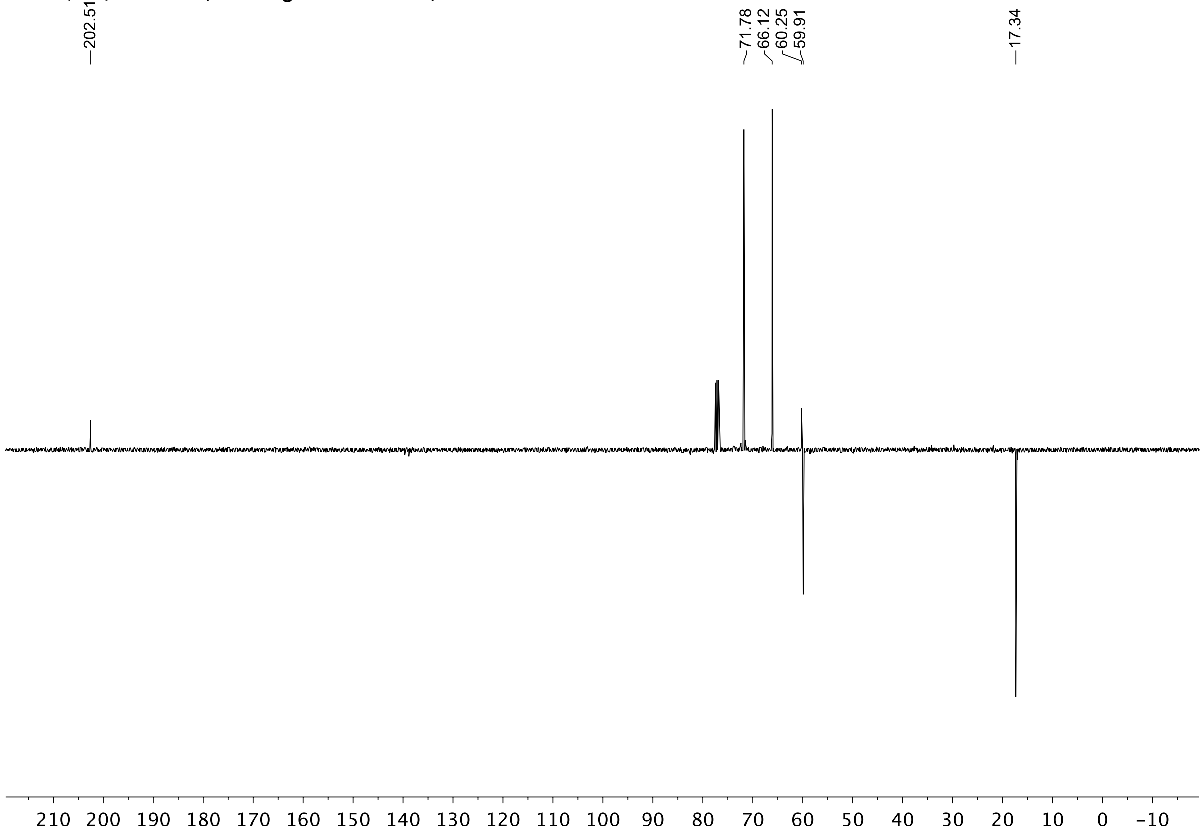

4-Chloro-5-methyl-2*H*-pyran-3(6*H*)-one (4)

^{1}H NMR ($CDCl_3$, 400 MHz)

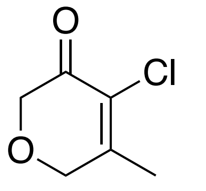

4

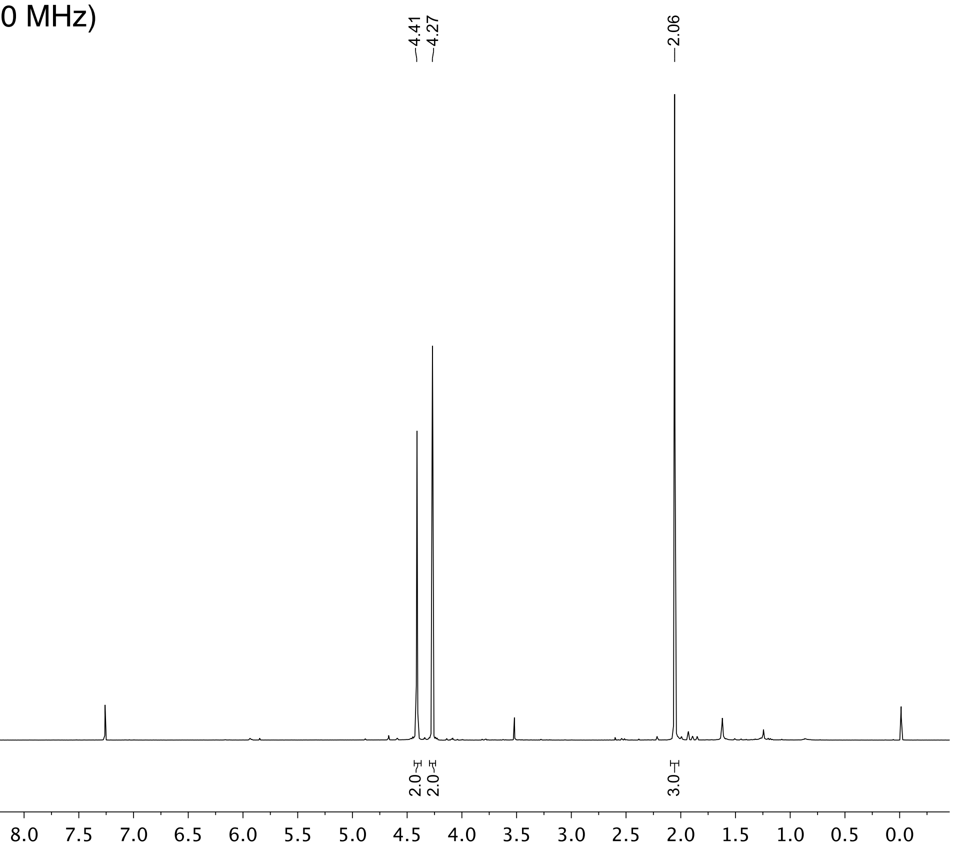

$^{13}C\{^{1}H\}$ NMR ($CDCl_3$, 101 MHz)

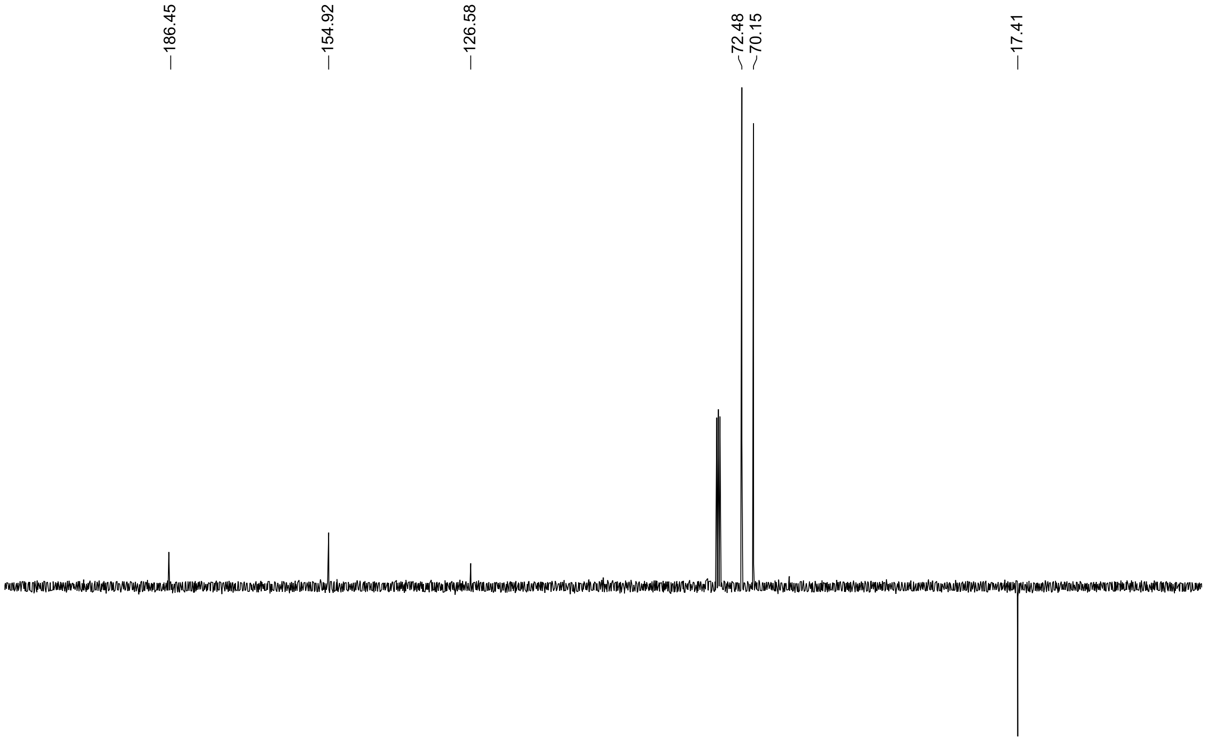

(3*S*,4*S*,5*R*)-4-Chloro-5-methyltetrahydro-2*H*-pyran-3-ol (*trans*,*trans*-5)

^{1}H NMR ($CDCl_3$, 400 MHz)

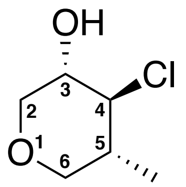

(3*S*,4*S*,5*R*)-**5**

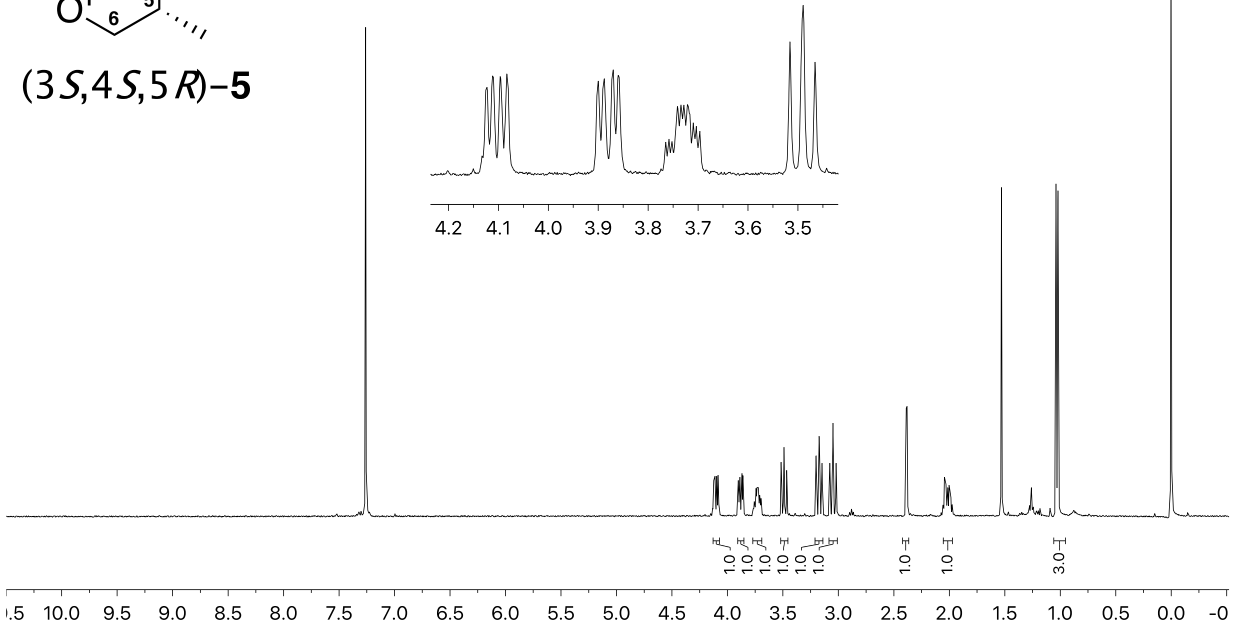

^{1}H-^{1}H COSY NMR ($CDCl_3$, 400 MHz)

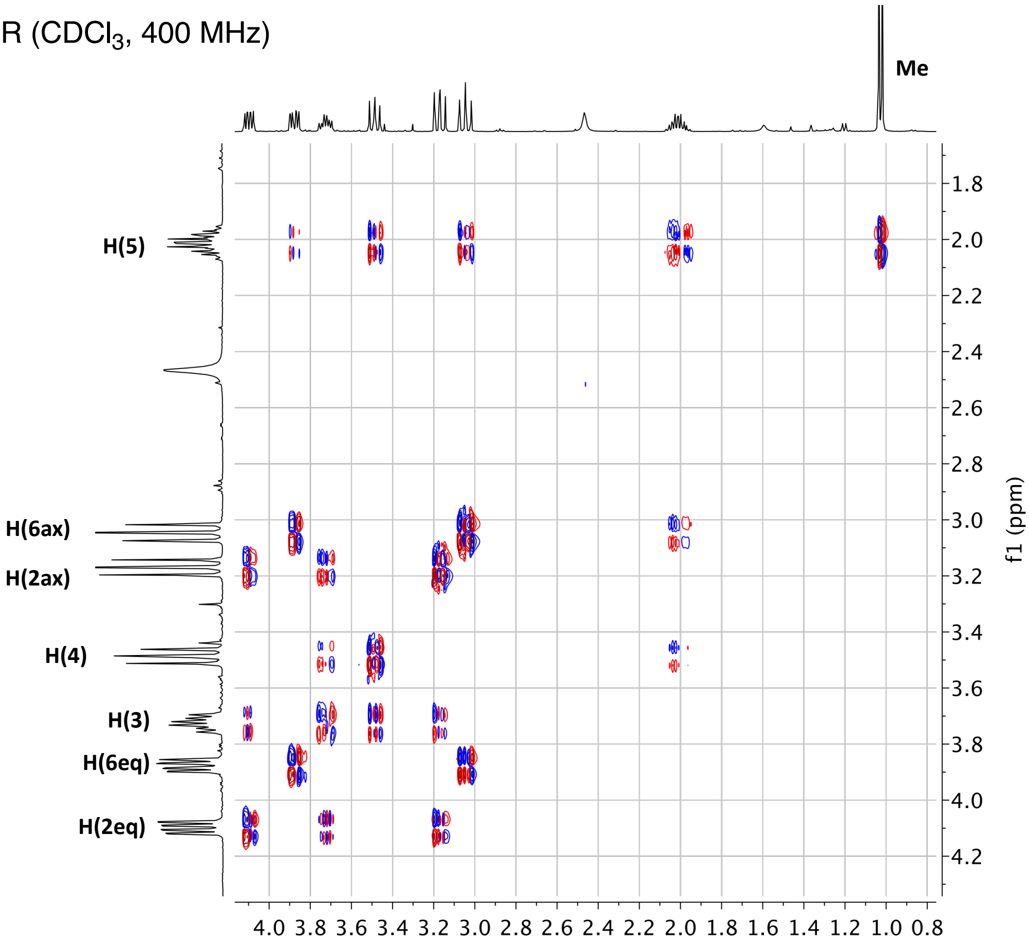

$^{13}C\{^1H\}$ NMR ($CDCl_3$, 101 MHz)

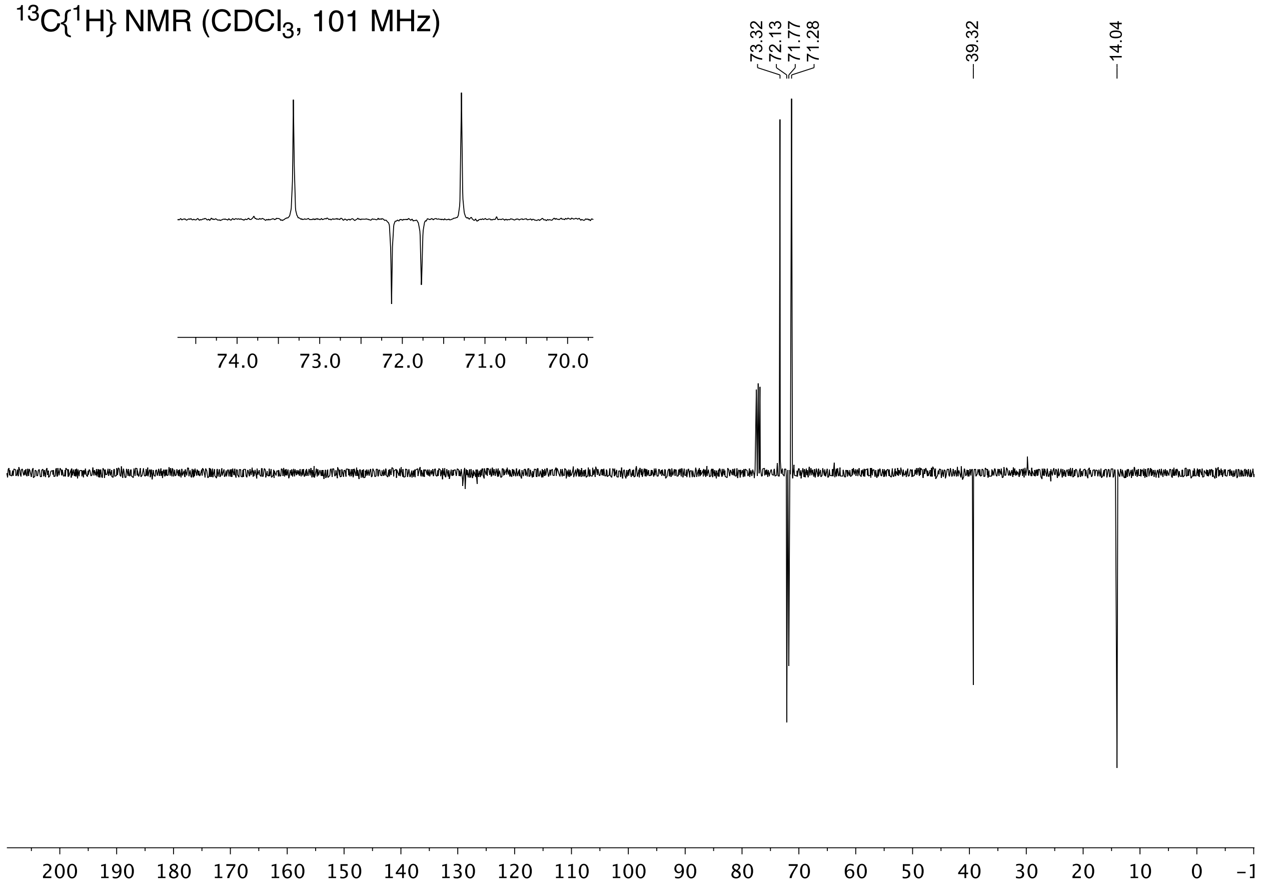

(3*R*,4*S*,5*R*)-4-Chloro-5-methyltetrahydro-2*H*-pyran-3-ol (*cis,trans*-5)

^{1}H NMR ($CDCl_3$, 400 MHz)

(3*R*,4*S*,5*R*)-**5**

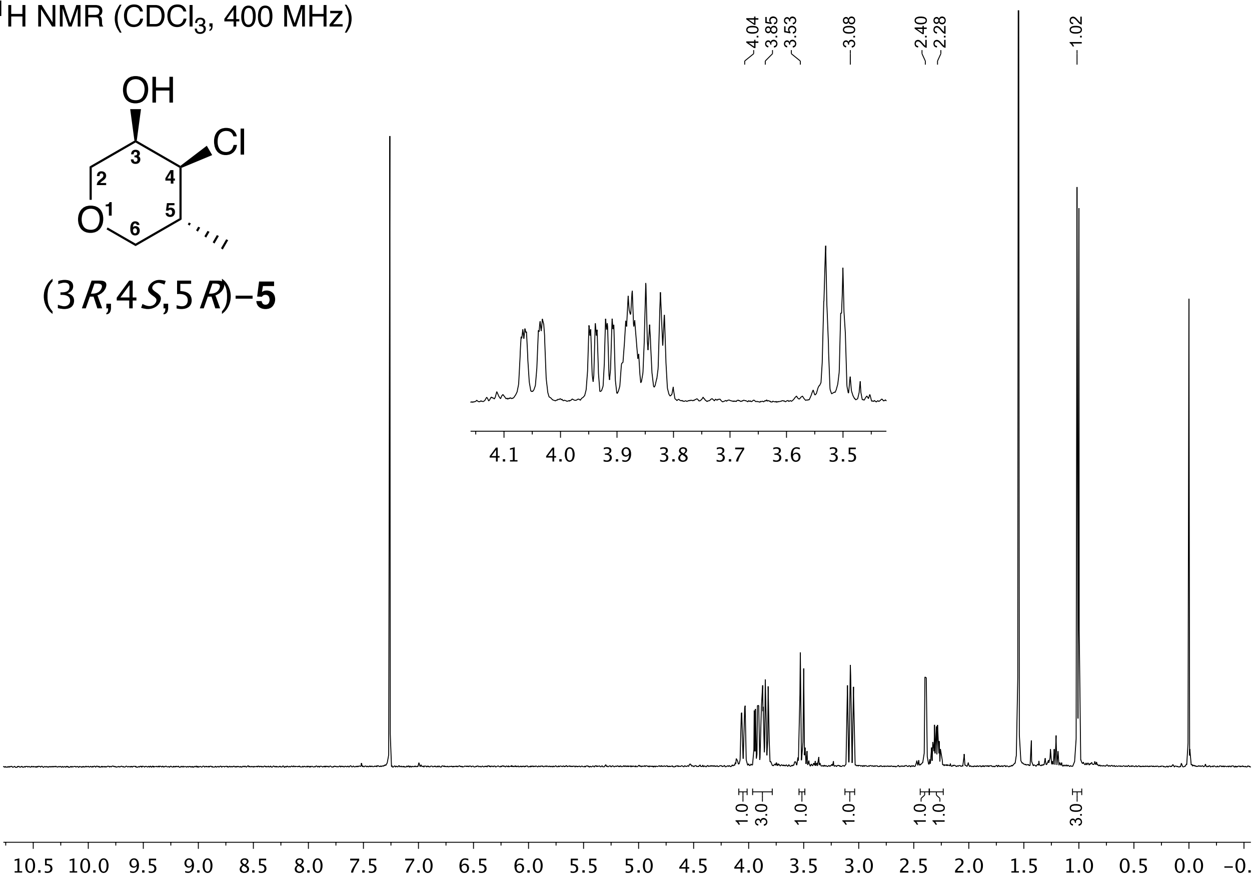

^{1}H-^{1}H COSY NMR ($CDCl_3$, 400 MHz)

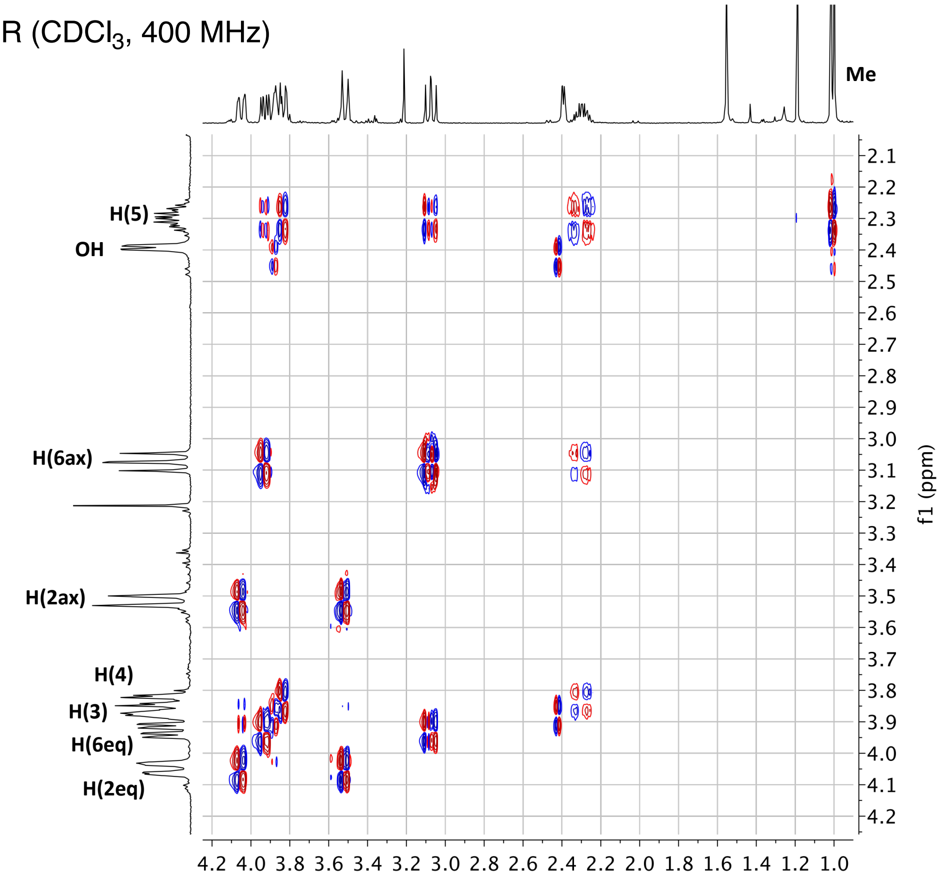

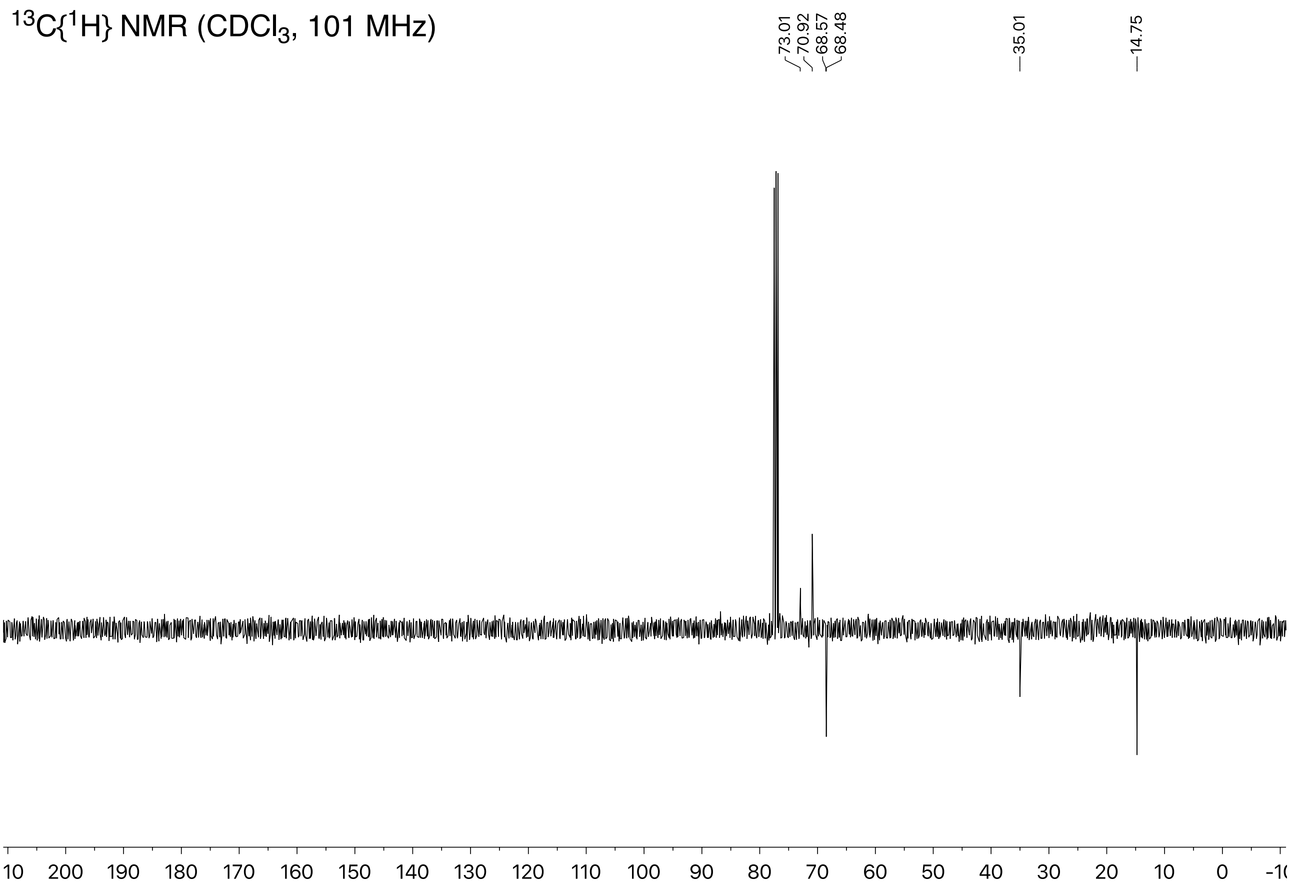
$^{13}C\{^1H\}$ NMR ($CDCl_3$, 101 MHz)
73.01
70.92
68.57
68.48
35.01
14.75
10
200
190
180
170
160
150
140
130
120
110
100
90
80
70
60
50
40
30
20
10
0

(3*S*,4*R*,5*R*)-5-Methyl-3,7-dioxabicyclo[4.1.0]heptane (6)

^{1}H NMR ($CDCl_3$, 400 MHz)

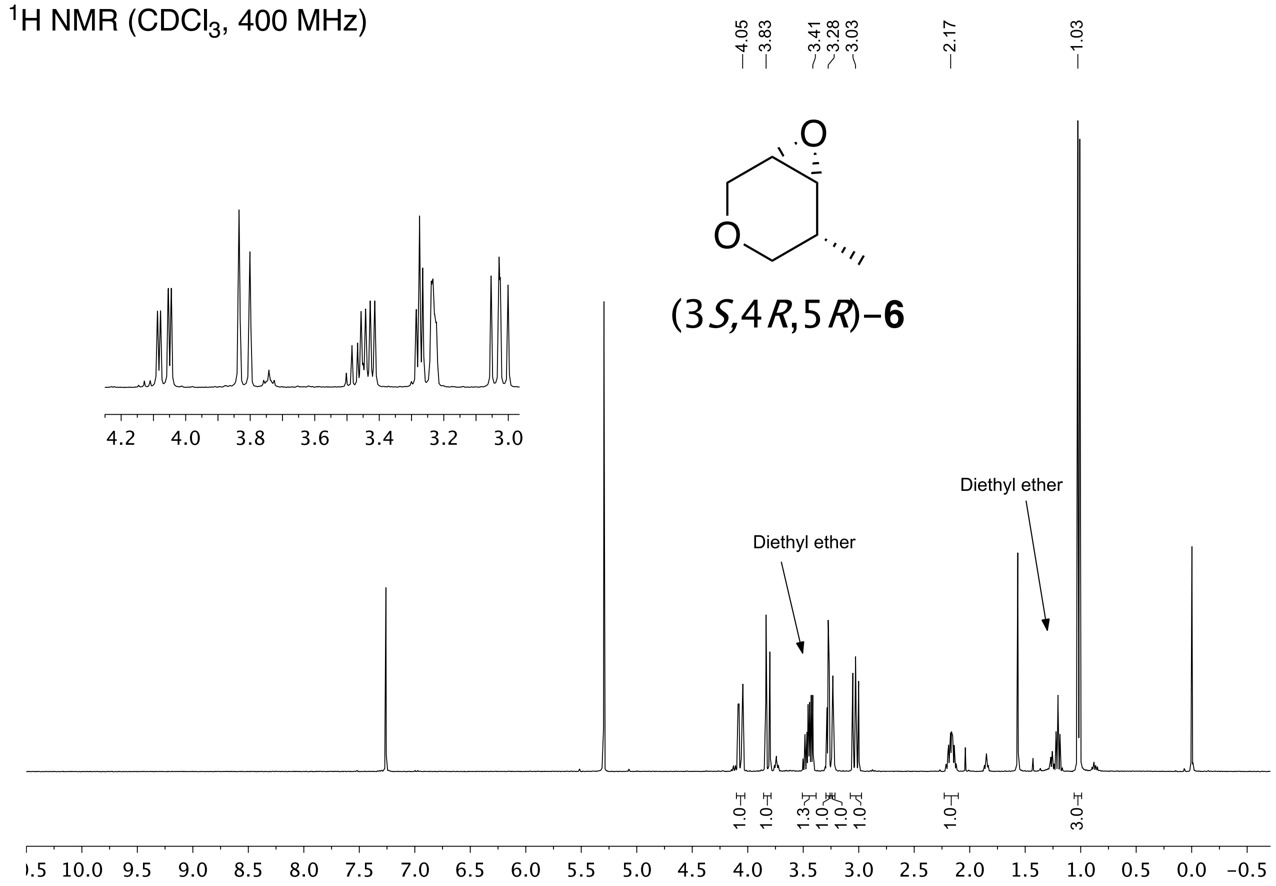

^{13}C{^{1}H} NMR ($CDCl_3$, 101 MHz)

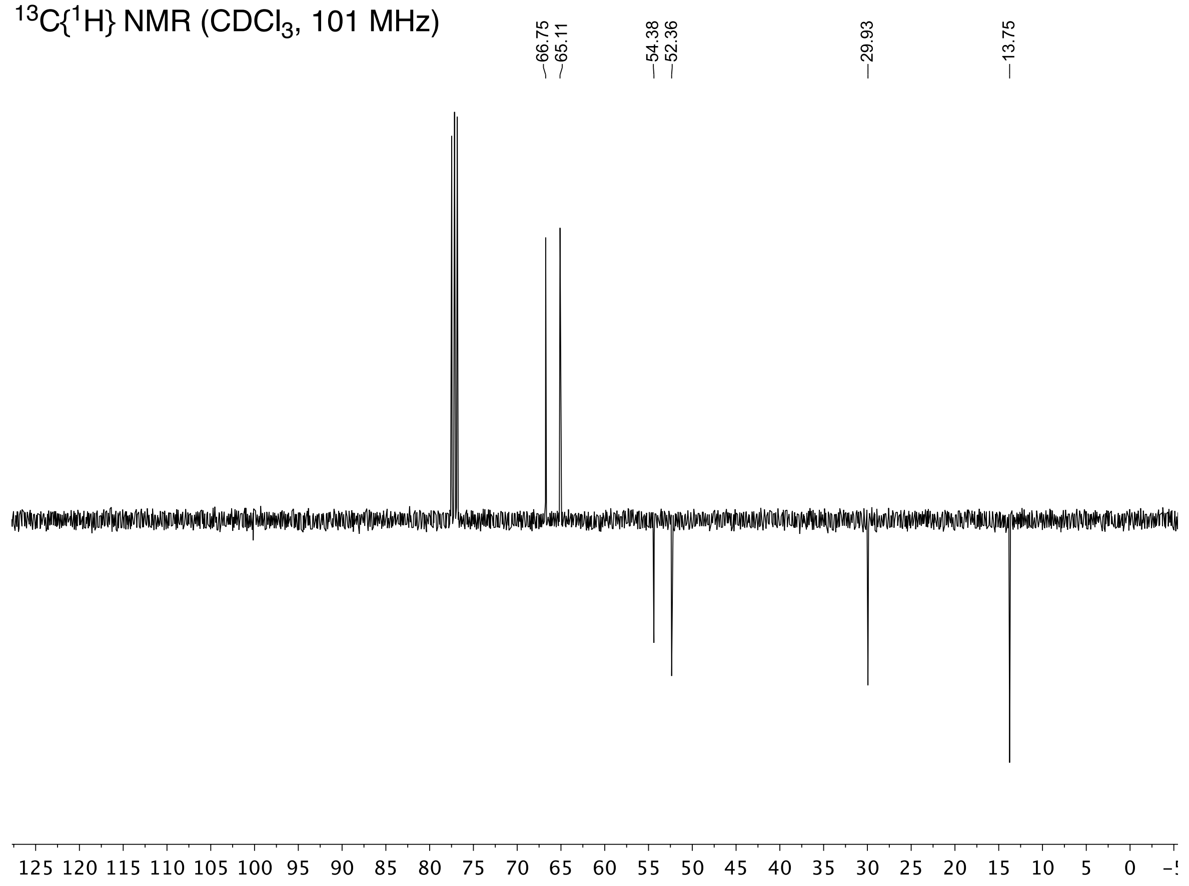

(3*R*,4*R*,5*R*)-3-Butyl-5-methyltetrahydro-2*H*-pyran-4-ol (7)

^{1}H NMR (C_6D_6, 400 MHz)

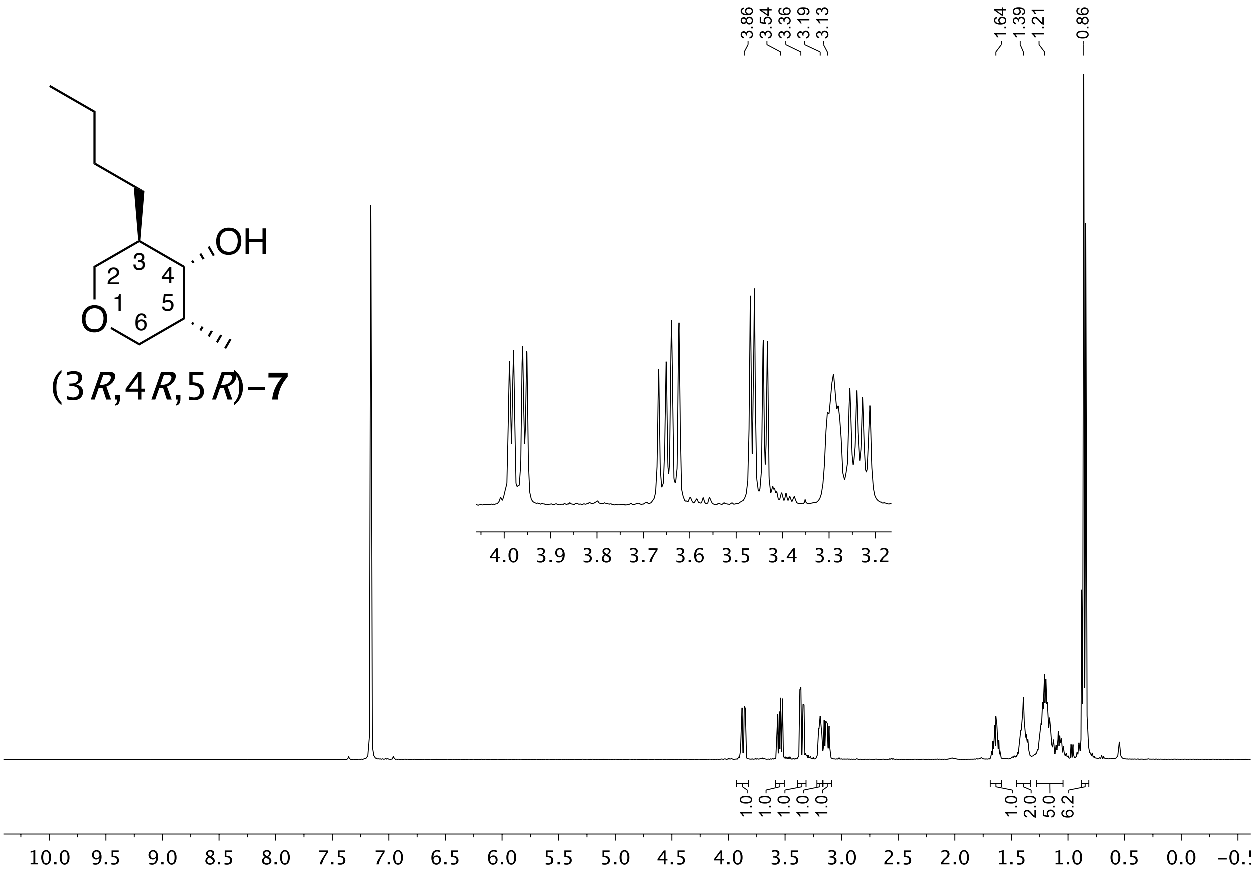

^{1}H-^{1}H COSY NMR (C_6D_6, 400 MHz)

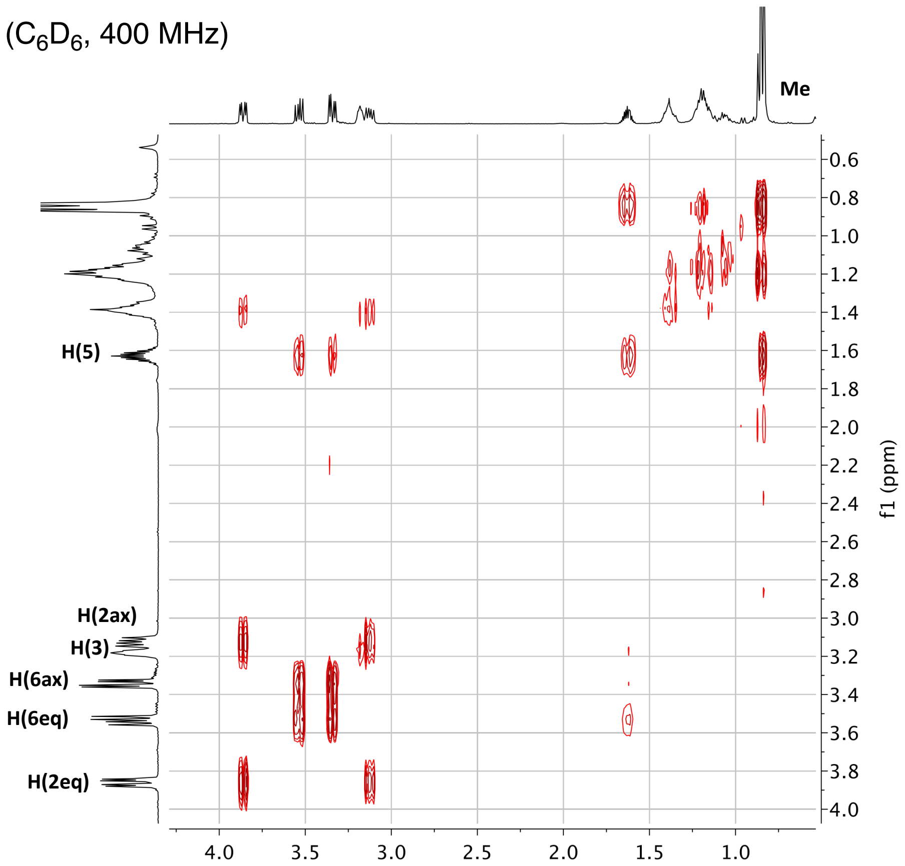

$^{13}C\{^{1}H\}$ NMR (C_6D_6, 101 MHz)

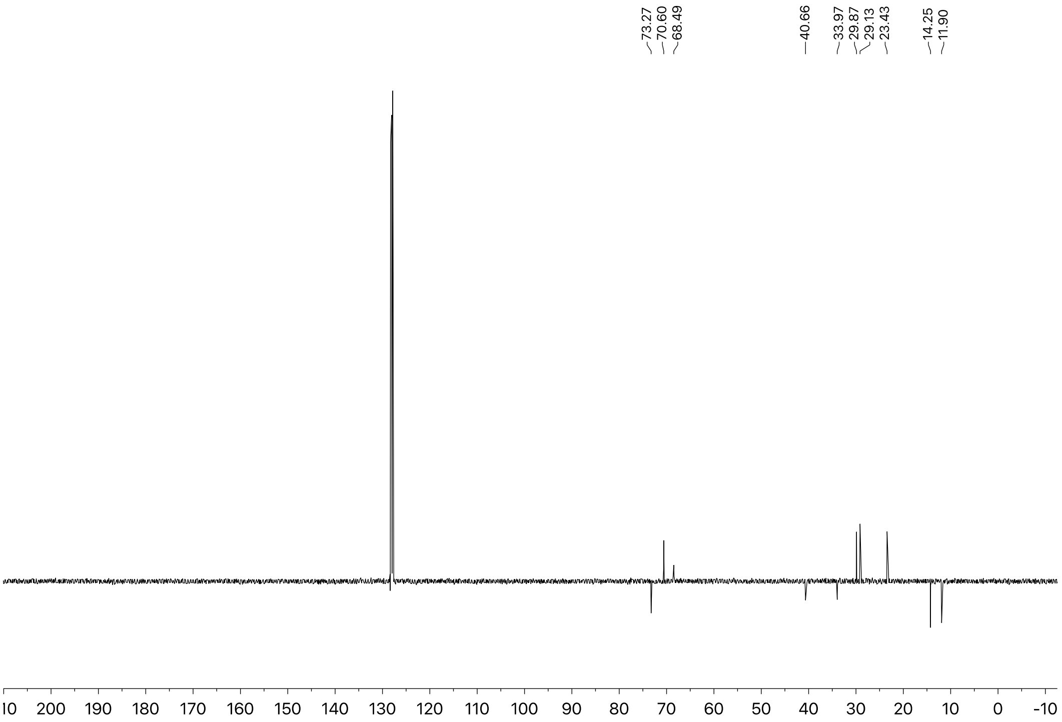

(3*R*,4*S*,5*S*)-4-Butyl-5-methyltetrahydro-2*H*-pyran-3-ol (7a)

^{1}H NMR ($CDCl_3$, 400 MHz)

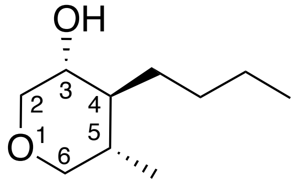

(3*R*,4*S*,5*S*)-**7a**

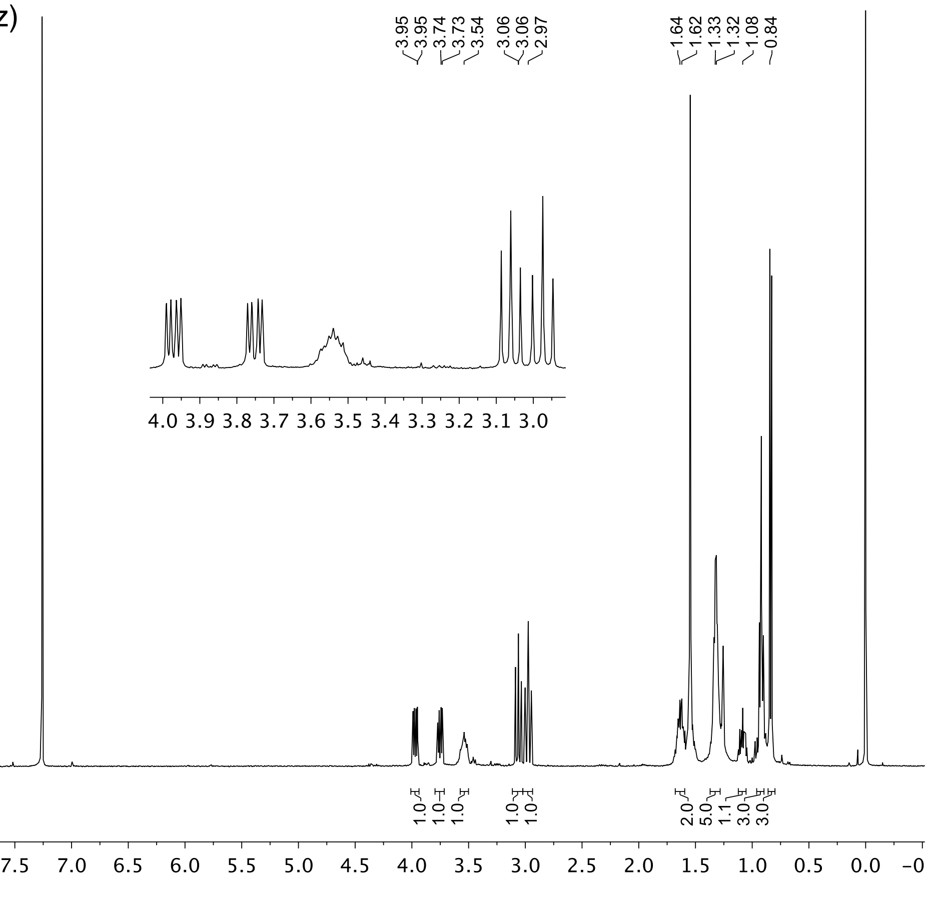

^{1}H-^{1}H COSY NMR ($CDCl_3$, 400 MHz)

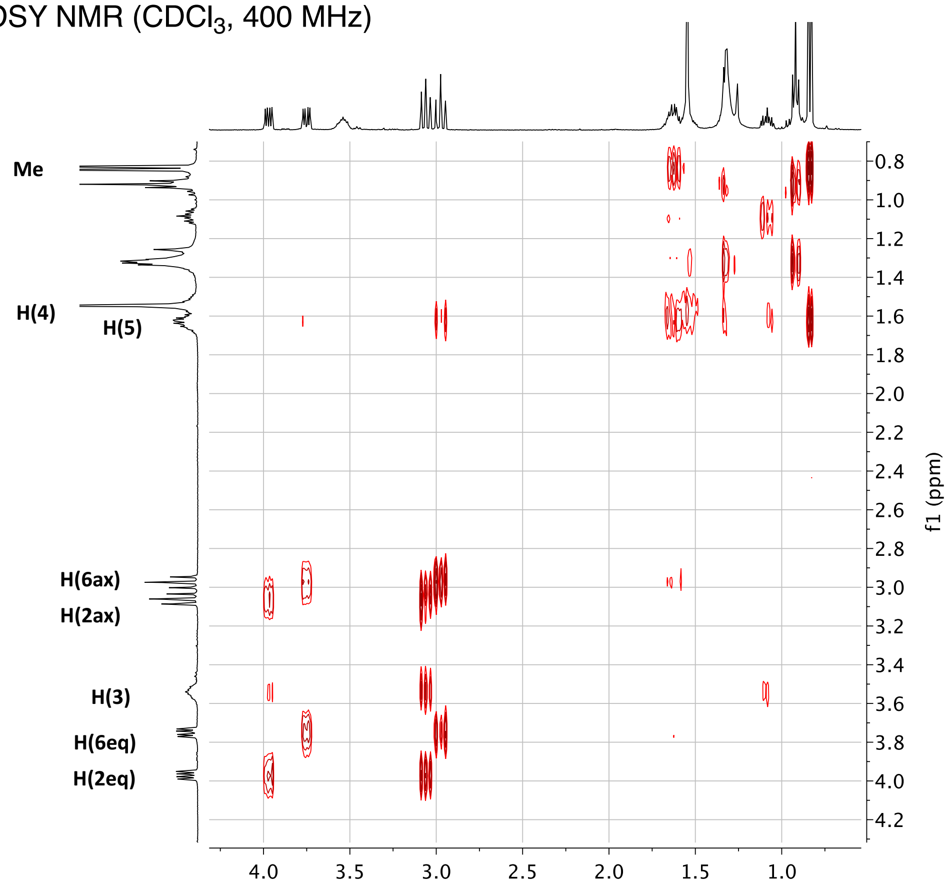

$^{13}C\{^1H\}$ NMR ($CDCl_3$, 101 MHz)

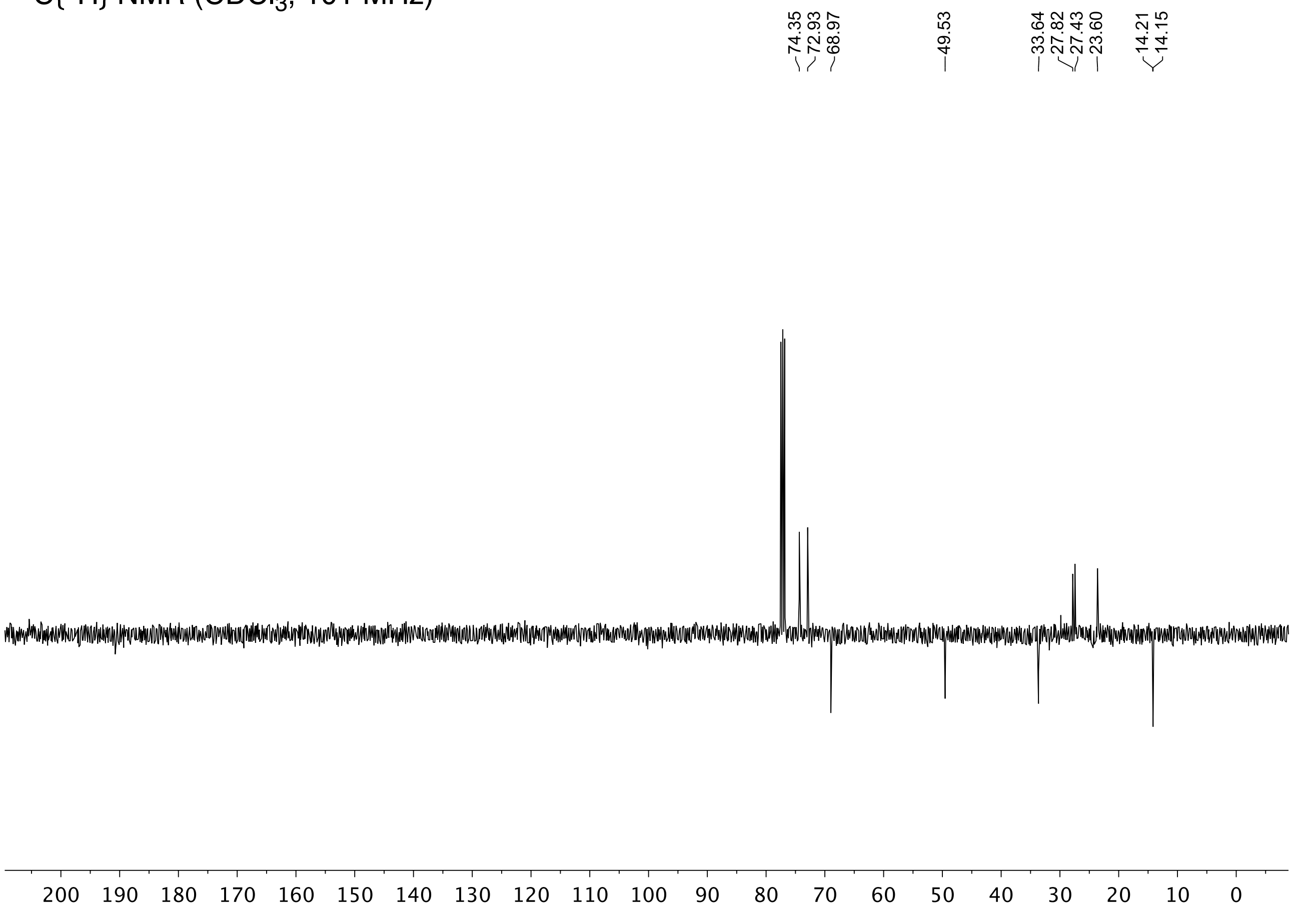

(3*R*,4*R*,5*R*)-3-Butyl-5-methyltetrahydro-2*H*-pyran-4-yl acetate (1)

^{1}H NMR ($CDCl_3$, 400 MHz)

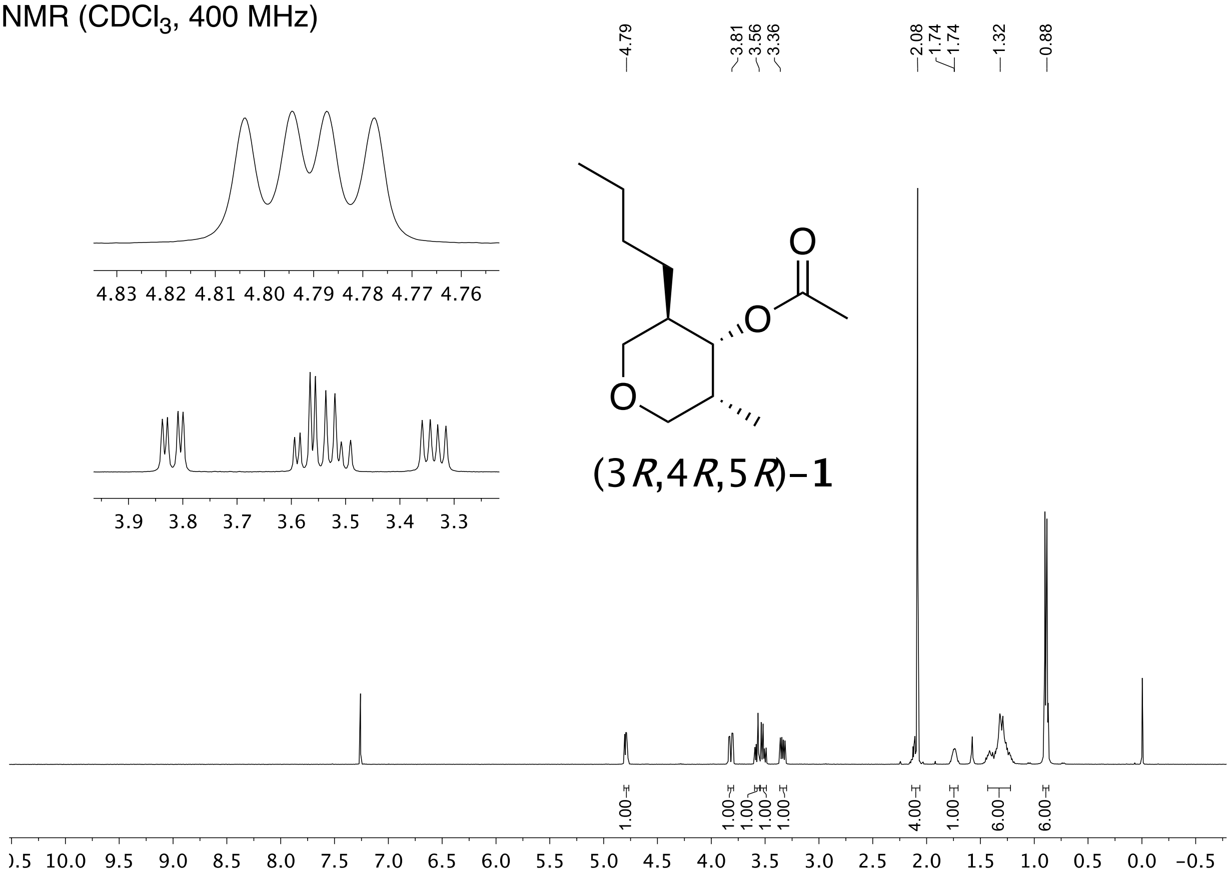

^{13}C{^{1}H} NMR ($CDCl_3$, 101 MHz)

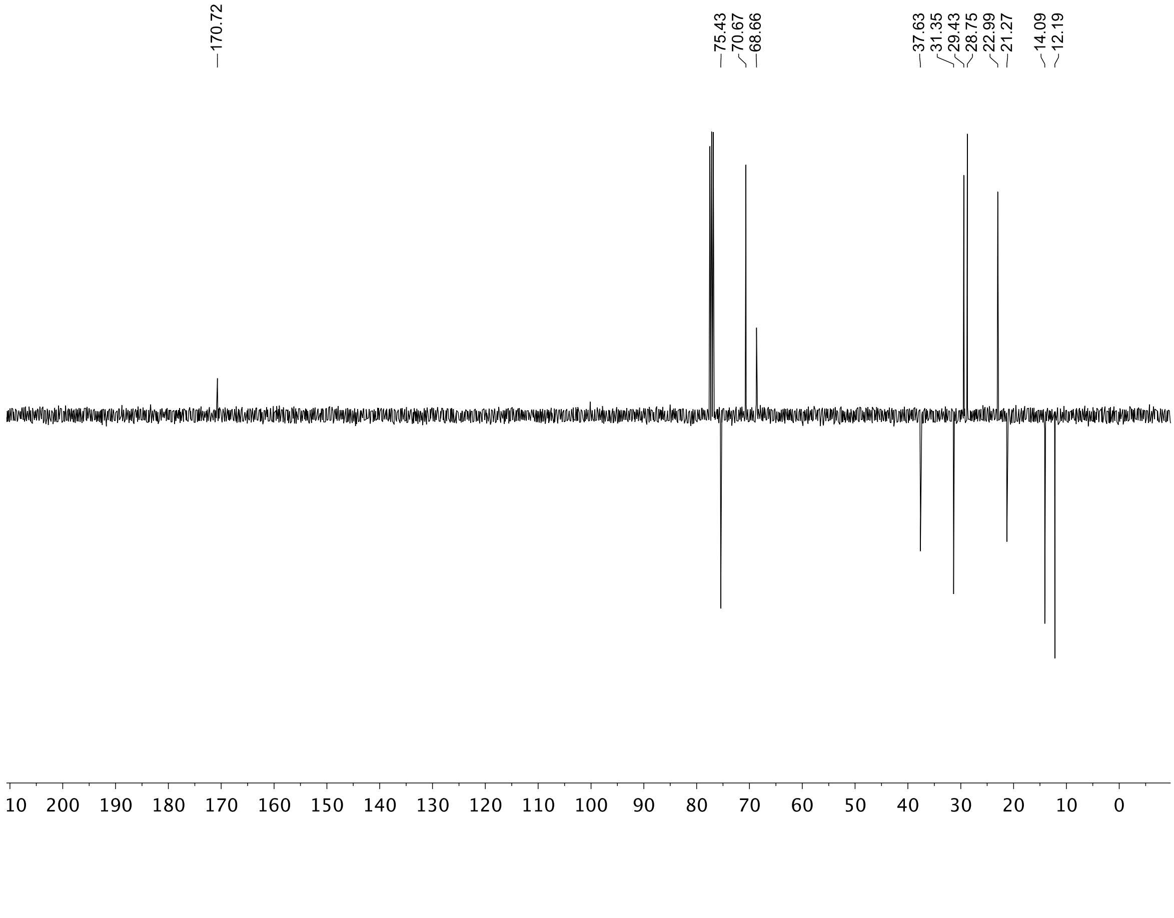

(3*R*,4*R*,5*R*)-3-(Benzylamino)-5-methyltetrahydro-2*H*-pyran-4-ol (8)

^1H NMR ($CDCl_3$, 400 MHz)

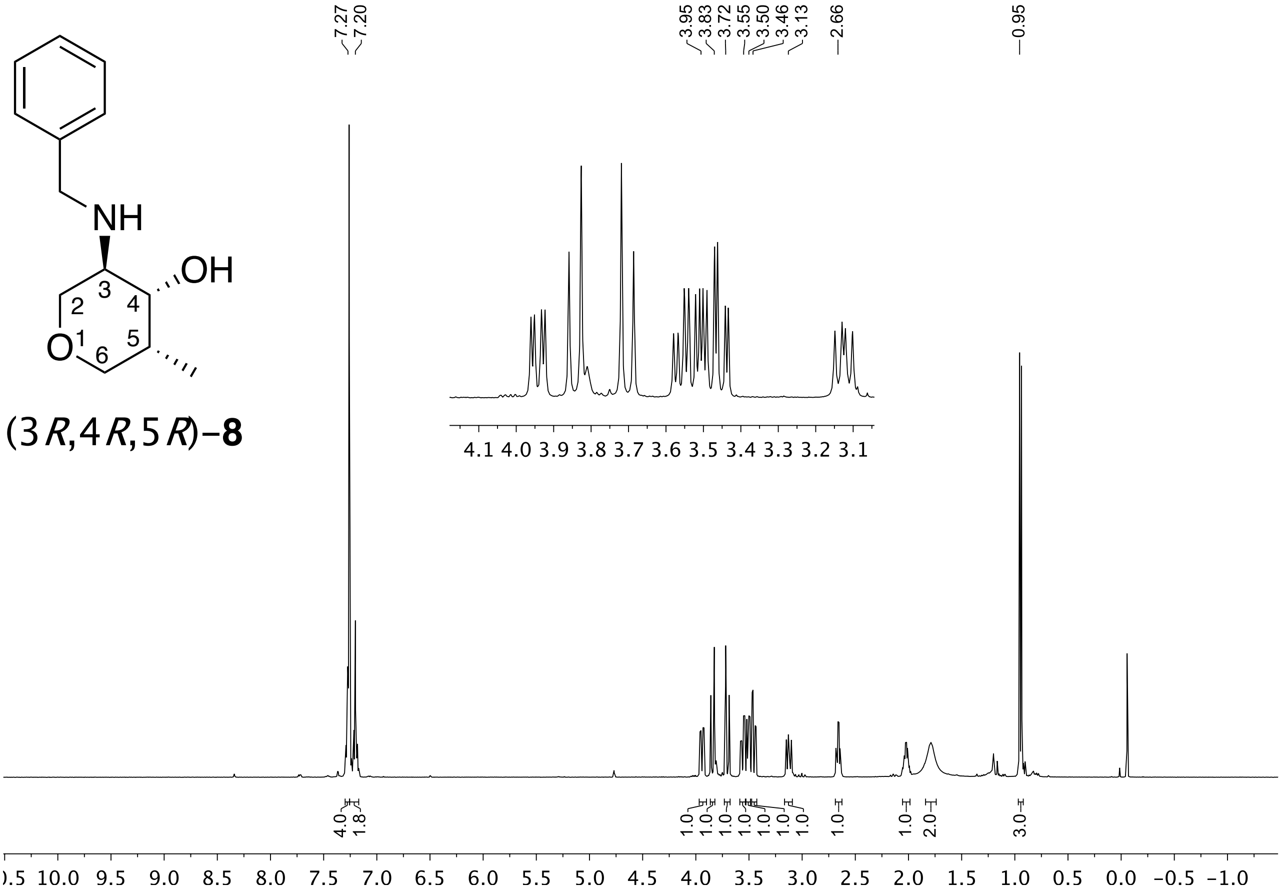

^1H-^1H COSY NMR ($CDCl_3$, 400 MHz)

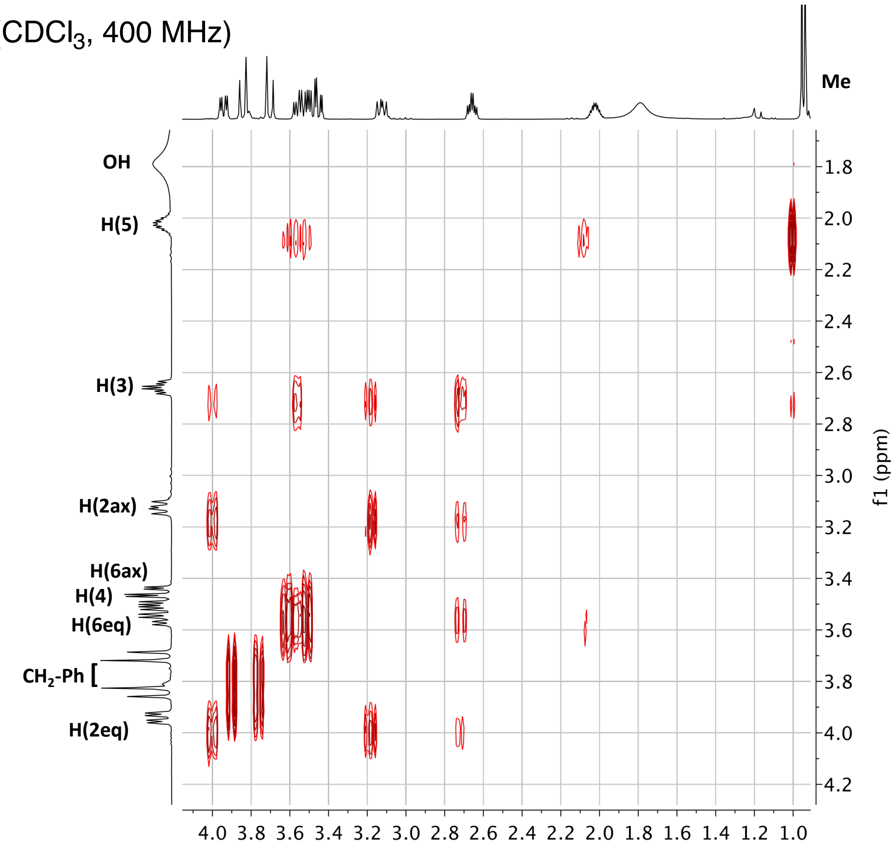

$^{13}C\{^{1}H\}$ NMR ($CDCl_3$, 101 MHz)

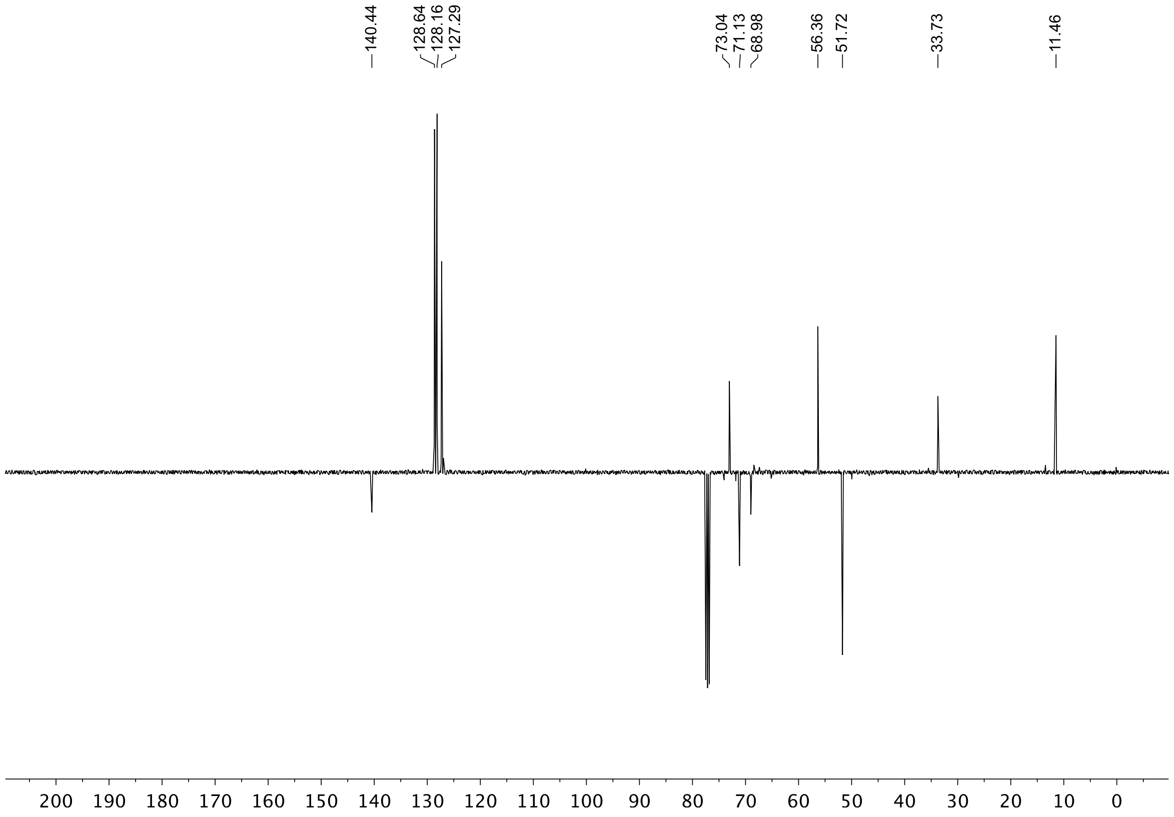

(2*R*,3*R*)-2-Methyl-4-methyleneoctane-1,3-diol (9)

^{1}H NMR ($CDCl_3$, 400 MHz)

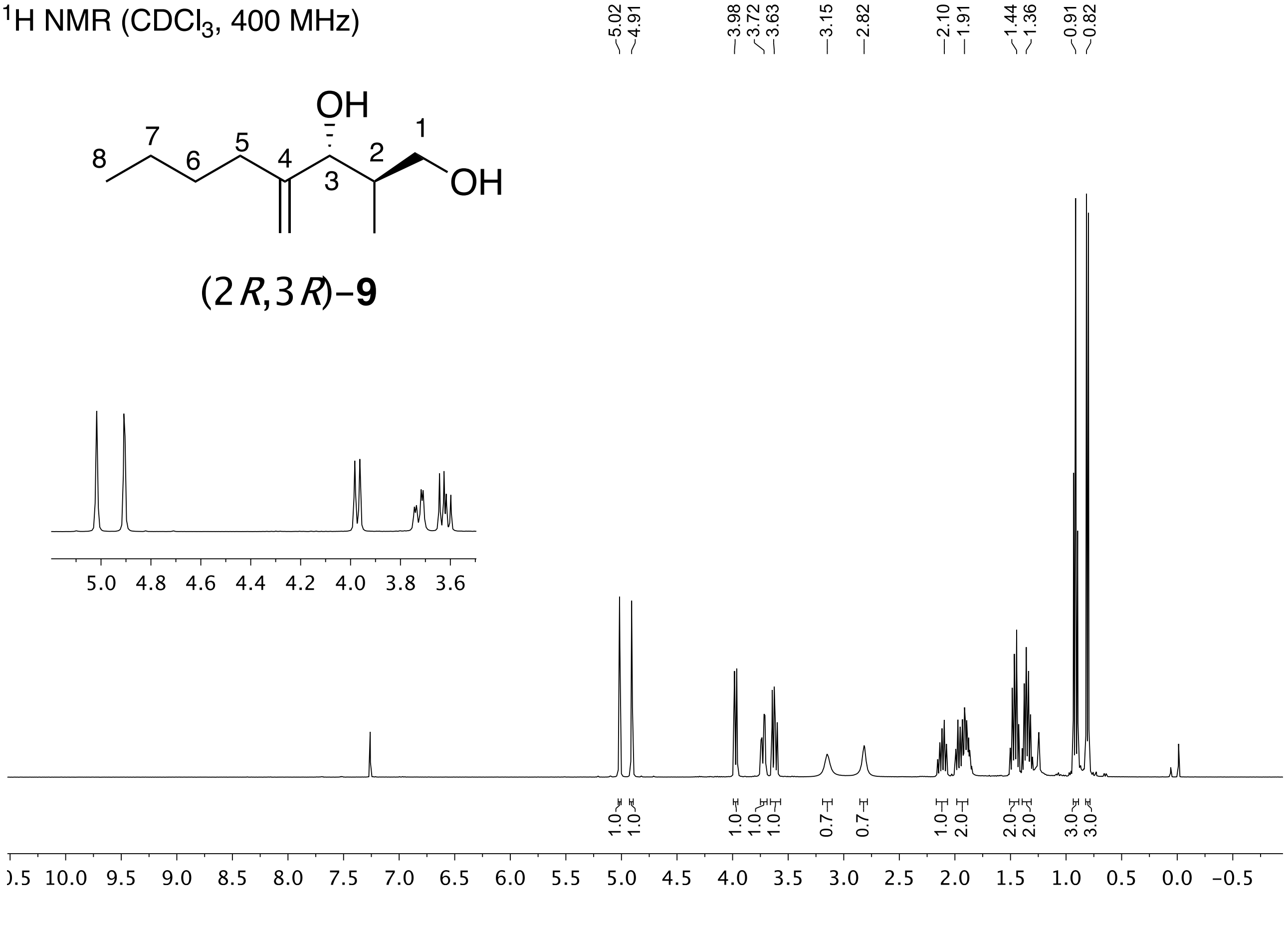

^{1}H-^{1}H COSY NMR ($CDCl_3$, 400 MHz)

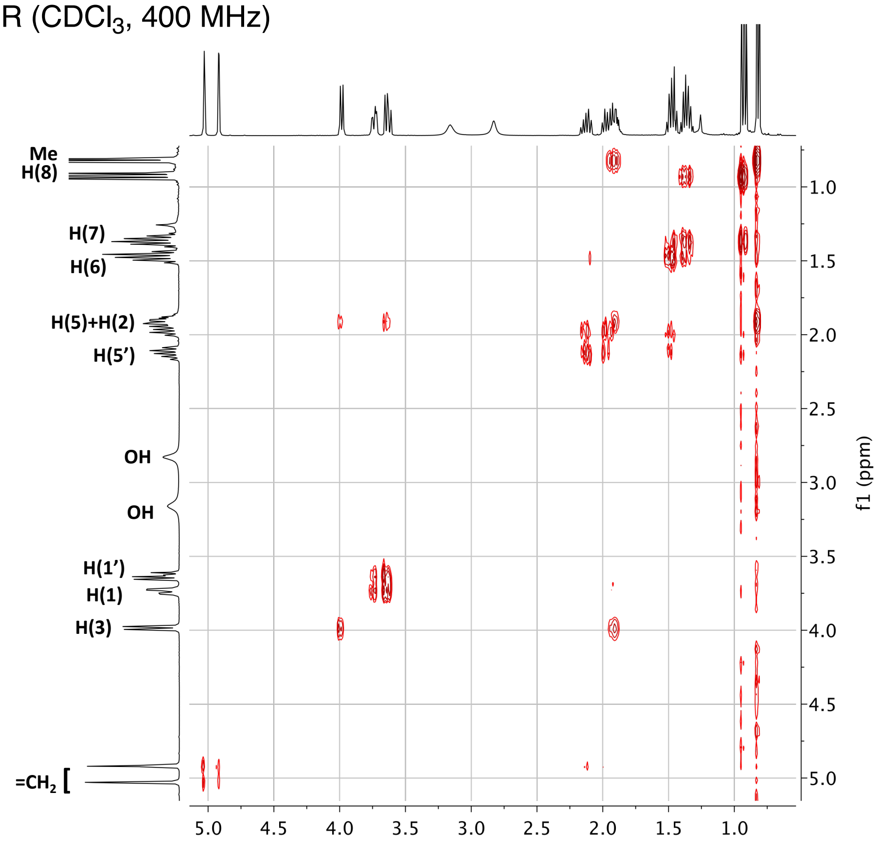

^{13}C{^{1}H} NMR ($CDCl_3$, 101 MHz)

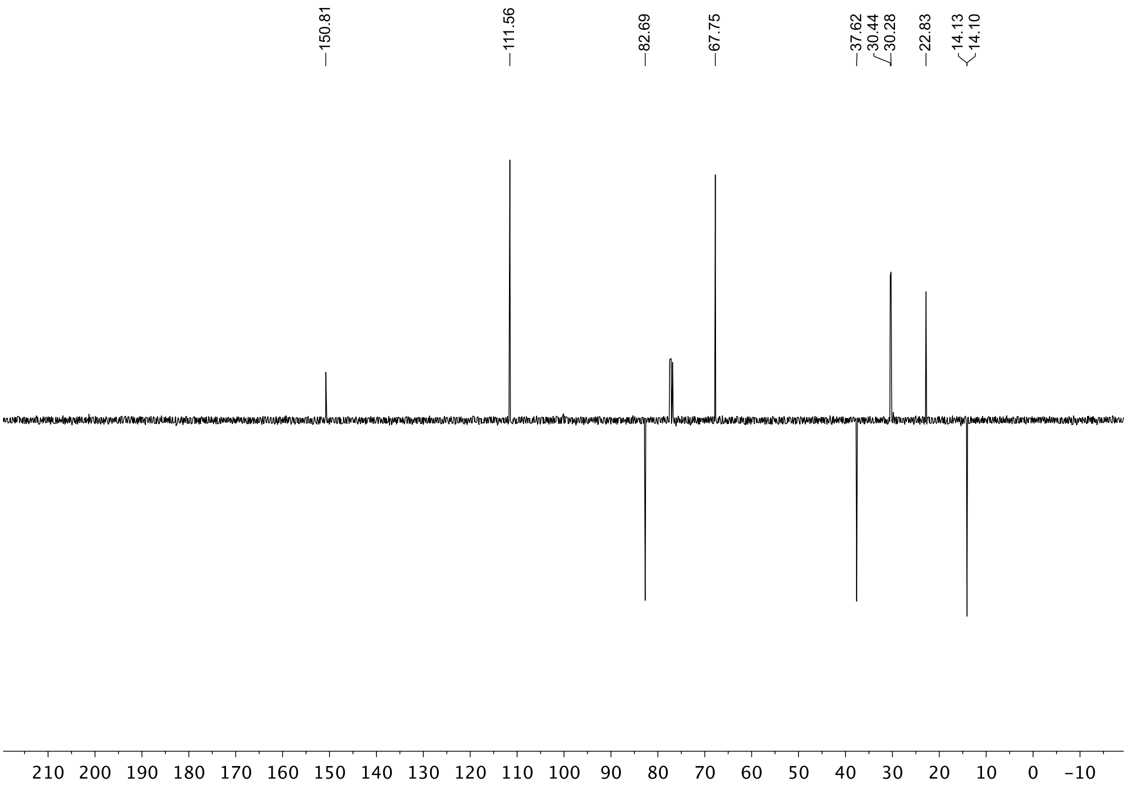

References

[1] Mathew, S.; Trajkovic, M.; Kumar, H.; Nguyen, Q.-T.; Fraaije, M. W. *Chem. Commun.* **2018**, *54*, 11208−11211.
[2] Bechtold, M.; Brenna, E.; Femmer, C.; Gatti, F. G.; Panke, S.; Parmeggiani, F.; Sacchetti, A. *Org. Process. Res. Dev.* **2012**, *16*, 269-276.
[3] Nguyen, Q.-T.; Trinco, G.; Binda, C.; Mattevi, A.; Fraaije, M. W. *Appl. Microbiol. Biotechnol.* **2017**, *101*, 2831−2842.
[4] Beyer, N.; Kulig, J., K.; Bartsch, A.; Hayes, M., A.; Janssen, D., B.; Fraaije, M., W. *Appl. Microbiol. Biotechnol.* **2017**, *101*, 2319-2331.
[5] Skinnemoen, K.; Undheim, K. *Acta Chem. Scand.* **1980**, *34*, 295-297.
[6] Juliá, S.; Guixer, J.; Masana, J.; Rocas, J.; Colonna, S.; Annuziata, R.; Molinari, H. *J. Chem. Soc., Perkin Trans* **1982**, *1*, 1317.
[7] Montalban, A. G.; Wittenberg, L.; McKillop, A. *Tetrahedron Lett.* **1999**, *40*, 5893-5896.
[8] Seebach, D.; Prelog, V. *Angew. Chem., Int. Ed. Engl.* **1982**, *21*, 654–660.
[9] Eis, M. J.; Wrobel, J. E.; Ganem, B. *J. Am. Chem. Soc.* **1984**, *106*, 3693-3694.
[10] Abate, A.; Brenna, E.; Fronza, G.; Fuganti, C.; Gatti, F. G.; Maroncelli, S. *Chem. Biodiversity* **2006**, *3*, 677-694.
[11] Chini, M.; Crotti, P.; Gardelli, C.; Macchia, F. *Tetrahedron Lett.* **1994**, *50*, 1261-1274.
[12] Hodgson, D. M.; Stent, M. A. H.; Wilson, F. X. *Synthesis* **2002**, *10*, 1445-1453.
[13] Gaussian 16, Revision C.01, M. J. Frisch, G. W. Trucks, H. B. Schlegel, G. E. Scuseria, M. A. Robb, J. R. Cheeseman, G. Scalmani, V. Barone, G. A. Petersson, H. Nakatsuji, X. Li, M. Caricato, A. V. Marenich, J. Bloino, B. G. Janesko, R. Gomperts, B. Mennucci, H. P. Hratchian, J. V. Ortiz, A. F. Izmaylov, J. L. Sonnenberg, D. Williams-Young, F. Ding, F. Lipparini, F. Egidi, J. Goings, B. Peng, A. Petrone, T. Henderson, D. Ranasinghe, V. G. Zakrzewski, J. Gao, N. Rega, G. Zheng, W. Liang, M. Hada, M. Ehara, K. Toyota, R. Fukuda, J. Hasegawa, M. Ishida, T. Nakajima, Y. Honda, O. Kitao, H. Nakai, T. Vreven, K. Throssell, J. A., Jr. Montgomery, J. E. Peralta, F. Ogliaro, M. J. Bearpark, J. J. Heyd, E. N. Brothers, K. N. Kudin, V. N. Staroverov, T. A. Keith, R. Kobayashi, J. Normand, K. Raghavachari, A. P. Rendell, J. C. Burant, S. S. Iyengar, J. Tomasi, M. Cossi, J. M. Millam, M. Klene, C. Adamo, R. Cammi, J. W. Ochterski, R. L. Martin, K. Morokuma, O. Farkas, J. B. Foresman and D. J. Fox, Gaussian, Inc., Wallingford CT, 2016.
[14] (a) Becke, A. D. *J. Chem. Phys.* **1993**, *98*, 5648–5652; (b) Lee, C.; Yang, W.; Parr, R. G. *Phys. Rev. Condens. Matter Mater. Phys.* **1988**, *37*, 785–789 .
[15] Grimme, S.; Antony, J.; Ehrlich, S.; Krieg, H.; *J. Chem. Phys.* **2010**, *132*, 154104.
[16] Head-Gordon, M.; People, J. A.; Frisch, M. J. *Chem. Phys. Lett.* **1988**, *153*, 503–506.
[17] Miertus, S.; Scrocco, E.; Tomasi, J. *Chem. Phys.* **1981**, *55*, 117−129.
[18] Zhao, Y.; Truhlar, D. G.; *Theor. Chem. Acc.,* **2008**, *120*, 215-241.
[19] CYLview, 1.0b; Legault, C. Y., Université de Sherbrooke, 2009 (http://www.cylview.org).
[20] Oxford Diffraction (2010). *CrysAlisPro* (Version 1.171.34.44). Oxford Diffraction Ltd., Yarnton, Oxfordshire, UK.
[21] Sheldrick, G. M. *Acta Cryst.* **2015**, *A71*, 3-8. Integrated space-group and crystal-structure determination.
[22] Flack, H. D.; Bernardinelli, G. *Acta Cryst.*, **1999**, *A55*, 908–915. Absolute structure and absolute configuration.
[23] Parsons,S.; Flack, H. D., Wagner, T. *Acta Cryst.,* **2013**, *B69*. 249-259. Use of intensity quotients and differences in absolute structure refinement.
[24] Sheldrick, G. M. *Acta Cryst.* **2015**, *C71*, 3-8. Crystal structure refinement with SHELXL.
[25] M. N. Burnett & C. K. Johnson (1996) ORTEP-III: Oak Ridge Thermal Ellipsoid Plot Program for Crystal Structure Illustrations, Oak Ridge National Laboratory Report ORNL-6895.
